# Supplementary material for: Cellular Differentiation of Human Monocytes Is Regulated by Time-Dependent Interleukin-4 Signaling and the Transcriptional Regulator NCOR2
Source: Immunity. 2017 Dec 19;47(6):1051–1066.e12. doi: 10.1016/j.immuni.2017.11.024 (PMC5772172; doi:10.1016/j.immuni.2017.11.024)
Supplement: Document S1. Figures S1–S6 [file mmc1.pdf]

Immunity, Volume *47*

Supplemental Information

Cellular Differentiation of Human Monocytes Is Regulated by Time-Dependent Interleukin-4 Signaling and the Transcriptional Regulator NCOR2

Jil Sander, Susanne V. Schmidt, Branko Cirovic, Naomi McGovern, Olympia Papantonopoulou, Anna-Lena Hardt, Anna C. Aschenbrenner, Christoph Kreer, Thomas Quast, Alexander M. Xu, Lisa M. Schmidleithner, Heidi Theis, Lan Do Thi Huong, Hermi Rizal Bin Sumatoh, Mario A.R. Lauterbach, Jonas Schulte-Schrepping, Patrick Günther, Jia Xue, Kevin Baßler, Thomas Ulas, Kathrin Klee, Natalie Katzmarski, Stefanie Herresthal, Wolfgang Krebs, Bianca Martin, Eicke Latz, Kristian Händler, Michael Kraut, Waldemar Kolanus, Marc Beyer, Christine S. Falk, Bettina Wiegmann, Sven Burgdorf, Nicholas A. Melosh, Evan W. Newell, Florent Ginhoux, Andreas Schlitzer, and Joachim L. Schultze

Supplementary figure 1

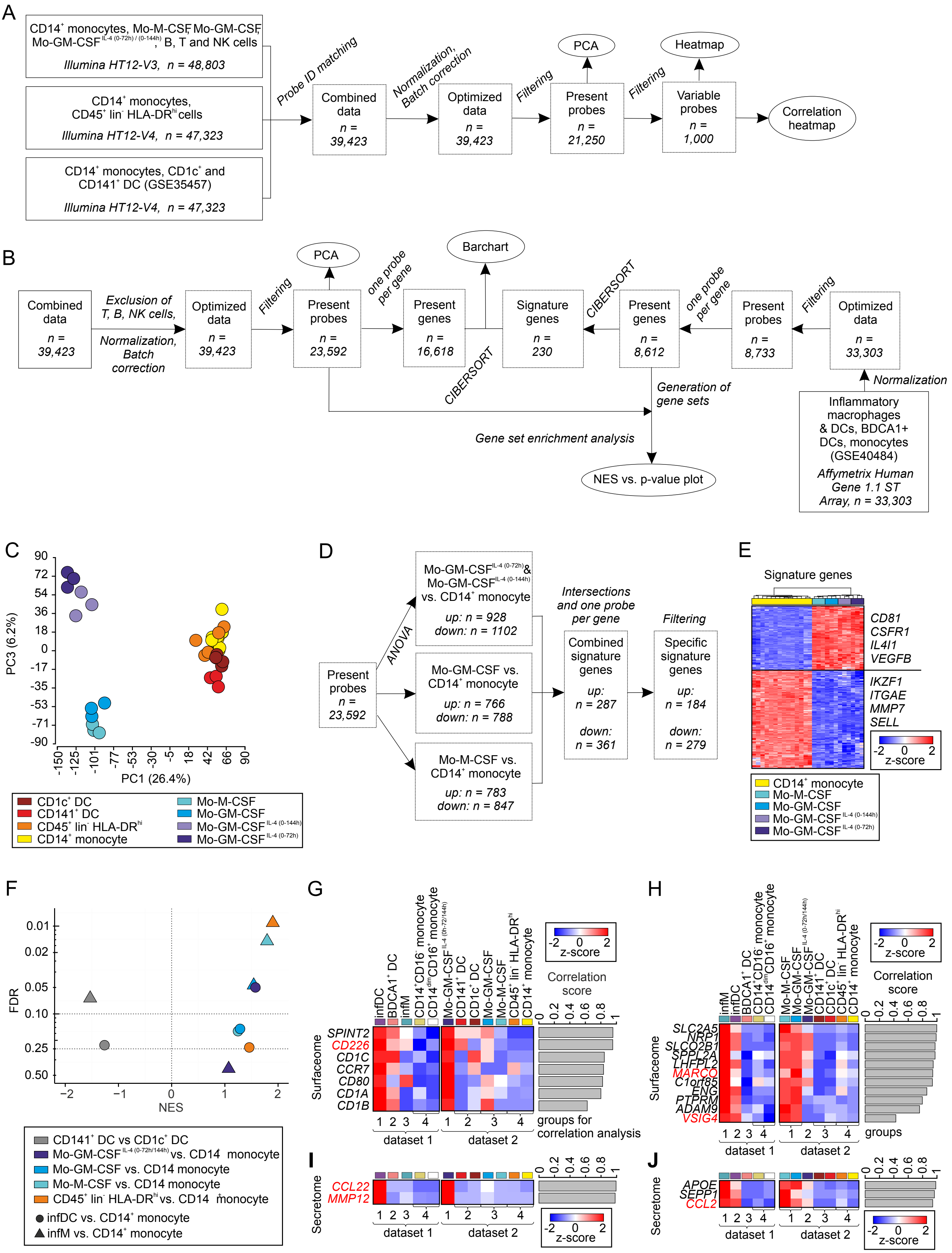

Figure S1 related to Figure 1

Relationship of *in vitro* activated monocyte-derived cells. (A-B) Analysis workflows for Figure 1 and S1 for datasets (**A**) with T, B and NK cells or (**B**) without. (**C**) PCA based on 23,952 present probes. Displayed are PCs 1 versus 3. (**D**) Workflow to identify specifically expressed genes in monocyte-derived cells compared to monocytes, or vice versa. (**E**) Heatmap of specifically expressed genes in monocyte-derived cells compared to monocytes, or vice versa. Z-transformed log_2-expression values scaled to a minimum of -2 (blue) and a maximum of 2 (red). Important genes of each group are depicted next to the heatmap. (**F**) Scatterplot showing the normalized enrichment score (NES, x-axis) versus the false discovery rate (FDR, y-axis) obtained after GSEA. Gene sets were comprised of specifically expressed genes in infM or infDCs compared to $CD14^+$ monocytes, respectively. Enrichments were estimated for pairwise comparisons of monocyte-derived cells versus $CD14^+$ Monocytes, and for the comparison of $CD141^+$ versus $CD1c^+$ DCs. (**G-J**) Heatmaps of genes encoding (**G, H**) surface markers or (**I, J**) secreted molecules specifically expressed in (**G, I**) infDCs compared to infM, $BDCA1^+$ DCs and monocytes (left, dataset 1), as well as in Mo-$GMCSF^{IL4 (0\text{-}72h)}$ cells compared to all other investigated monocyte-derived cells, $CD14^+$ monocytes and DCs (right, dataset 2), or in (**H, J**) infM compared to infDCs, $BDCA1^+$ DCs and monocytes (left, dataset 1), as well as in Mo-GMCSF and Mo-MCSF cells compared to Mo-$GMCSF^{IL4(0\text{-}72h)}$ cells, $CD14^+$ monocytes and DCs (right, dataset 2). Pearson correlation values were calculated between the indicated group patterns of dataset 1 versus dataset 2 and displayed as a barplot next to the heatmaps. Genes encoding (**G, H**) surface markers or (**I, J**) secreted molecules are displayed, with a correlation of at least 0.4 between the corresponding two datasets. Genes further analyzed in Figure 1 **I-K** are highlighted in red. Z-transformed log_2-expression values were and scaled to a minimum of -2 (blue) and a maximum of 2 (red).

Supplementary figure 2

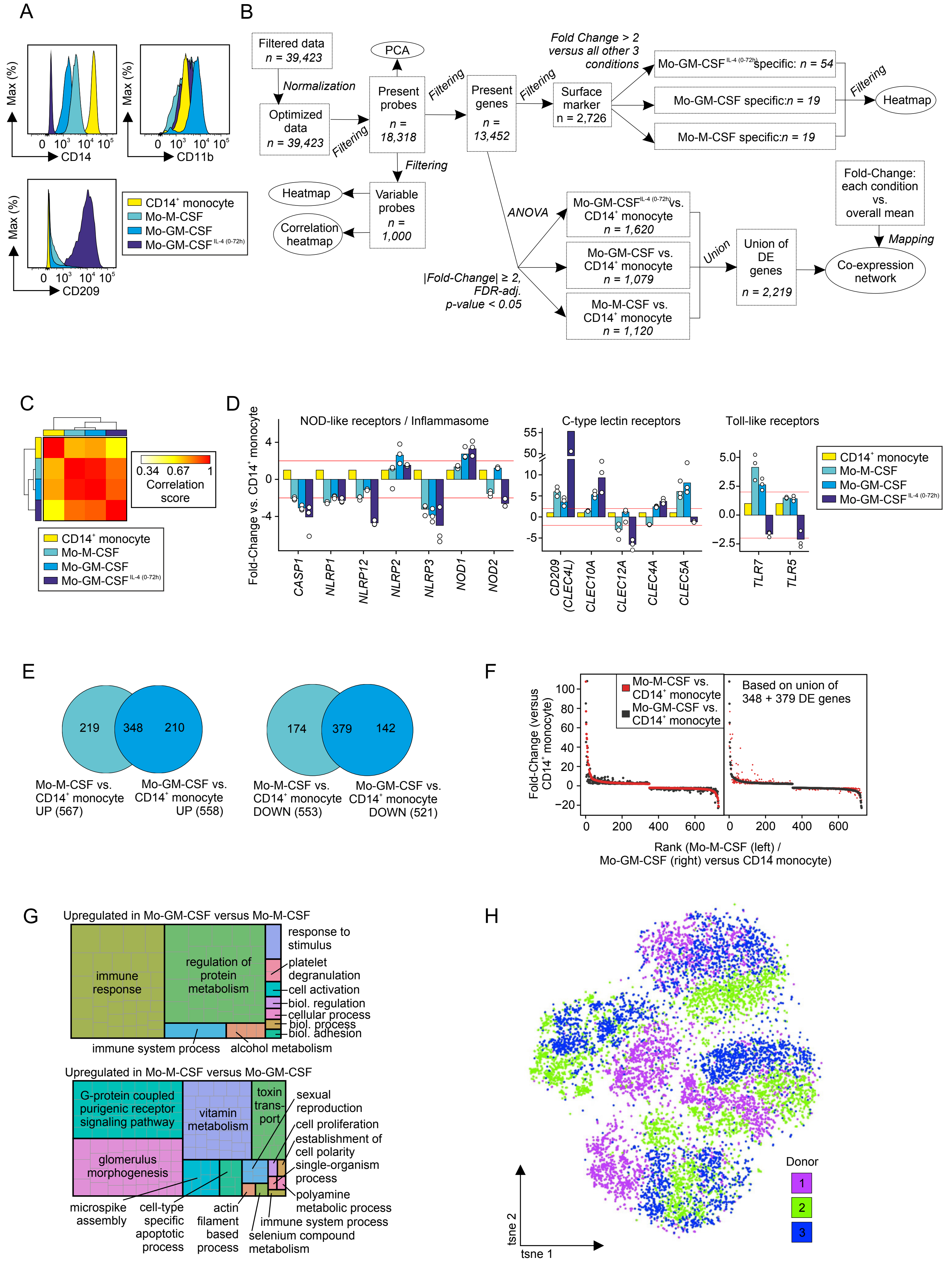

Figure S2 related to Figure 2

Mo-GMCSF$^{IL4(0-72h)}$ are most distinct from Mo-MCSF and Mo-GMCSF. (A) Histograms show relative expression of CD14, CD11b and CD209 on CD14^{+} monocytes, Mo-MCSF, Mo-GMCSF and Mo-GMCSF$^{IL4(0-72h)}$ analysed by flow cytometry. Representative data from 4 different donors is shown. (**B**) Workflow for the major bioinformatic analyses in Figure 2 and S2. (**C**) Heatmap visualizing pearson correlation values (pearson correlation coefficient matrix, PCCM) calculated pairwise between all cell types on the basis of the top 1000 most variable probes. The color scaling ranges from white (low correlation) via yellow to red (high correlation). (**D**) Barplots showing the Fold-Changes for all three monocyte-derived cell types compared to CD14^{+} monocytes for genes encoding NOD-like receptors or being linked to the inflammasome (top), as well as for genes encoding C-type lectin (bottom left) or Toll-like (bottom right) receptors. Additionally, the Fold-Changes for all replicates of a single cell type compared to the mean expression of the CD14^{+} monocyte replicates are displayed as dots. (**E**) Venn Diagrams of genes being significantly up- (Fold-Change > 2 and FDR-adjusted p-value < 0.05, top) or down-regulated (Fold-Change < -2 and FDR-adjusted p-value < 0.05, bottom) in whether Mo-MCSF (left) or Mo-GMCSF (right) compared to CD14^{+} monocytes. (**F**) Rank plots of genes being significantly up- or downregulated for both Mo-GMCSF and Mo-MCSF compared to CD14^{+} monocytes (union of both overlapping regions demonstrated in **E**). Displayed are the ranks (x-axis) of the Fold-Changes compared to CD14^{+} monocytes (y-axis), whether sorted according to Mo-MCSF (top) or Mo-GMCSF (bottom). (**G**) Tree plot summarizing significantly enriched (Enrichment p-value < 0.05) biological processes identified by Gene Ontology Enrichment Analysis (GOEA) for genes being significantly up- or downregulated (Fold-Change > 2 or < -2 and FDR-adjusted p-value < 0.05) directly between Mo-GMCSF and Mo-MCSF. (**H**) *t*-SNE composite dimensions (tsne1 and 2) of CD14^{+} monocytes, Mo-MCSF, Mo-GMCSF and Mo-GMCSF$^{IL4(0-144h)}$ analysed by mass cytometry and colored according to donor identity (n=3).

Supplementary figure 3

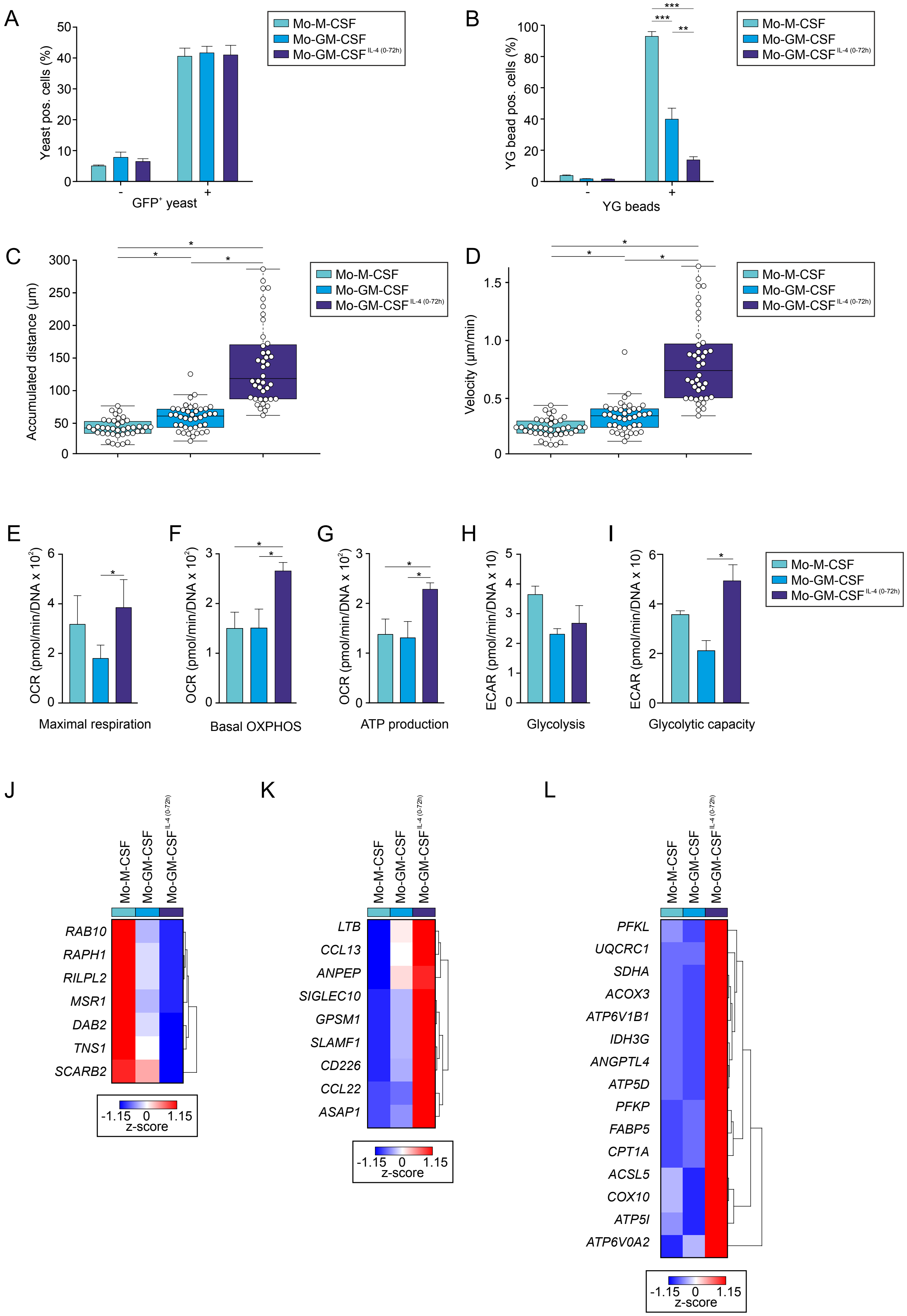

Figure S3 related to Figure 3

Prediction of activated monocyte functionality. (A) Quantification of the flow cytometric analysis of Mo-MCSF, Mo-GMCSF and Mo-GMCSF$^{IL4(0-72h)}$ after 1h of incubation with GFP^{+} yeast or without (n=3, mean + SEM). Statistical significance between cell types after 1h of incubation was determined using one-way RM ANOVA, with $p > 0.05$. **(B)** Quantification of the flow cytometric analysis of Mo-MCSF, Mo-GMCSF and Mo-GMCSF$^{IL4(0-72h)}$ after 4hrs of incubation with YG beads (n=5-6). Statistical significance was determined using one-way RM ANOVA and Tukey's method for multiple test correction, with $^{**}p < 0.01$ and $^{***}p < 0.001$. (**C**) Quantification of accumulated distance and (**D**) average migratory speed (velocity) of motile Mo-MCSF, Mo-GMCSF and Mo-GMCSF$^{IL4(0-72h)}$ corresponding to **Figure 3C**. Results show one representative experiment out of three. Statistical significance was determined using Kruskal-Wallis one-way ANOVA on ranks and Tukey's method for multiple test correction, with $^{*}p < 0.05$. (**E**) Maximal respiration, (**F**) basal OXPHOS, (**G**) ATP synthesis, (**H**) glycolysis and (**I**) glycolytic capacity Mo-MCSF, Mo-GMCSF and Mo-GMCSF$^{IL4(0-72h)}$, respectively. Results are pooled data shown as mean + SEM from n = 3 replicates. Statistical significance was determined using one way ANOVA with $^{*}p < 0.05$. (**J-L**) Heatmaps of genes being directly or indirectly linked (see supplementary information) to (**J**) YG bead uptake, (**K**) motility or (**L**) OCR/ECAR, which have expression patterns on the transcriptional level being analogous to the observed functional outcomes summarized in G. The $\log_2$-expression values were z-transformed and scaled to a minimum of –1.15 (blue) and a maximum of 1.15 (red), respectively.

Supplementary figure 4

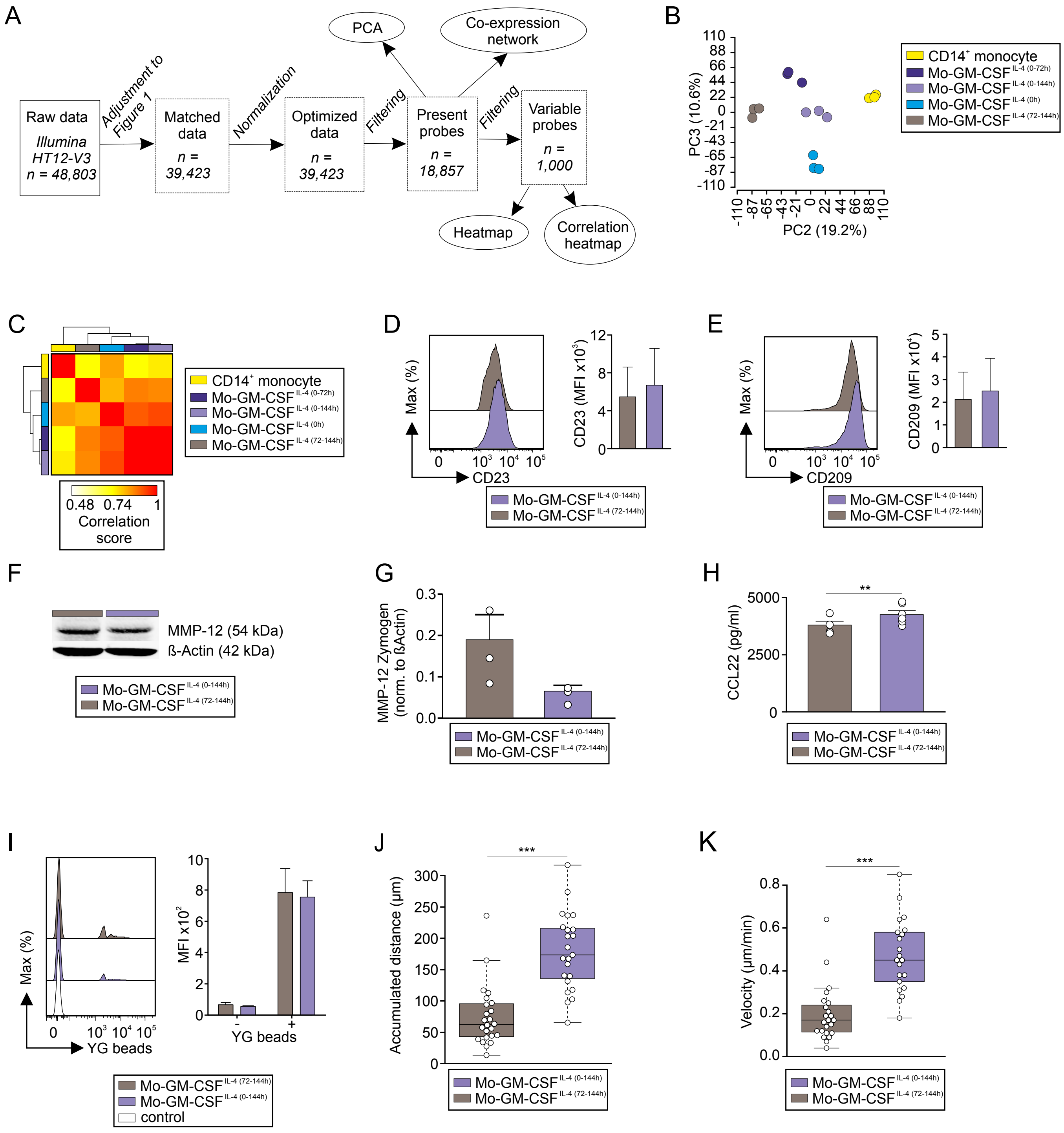

Figure S4 related to Figure 4

Mo-GMCSF$^{IL4(0\text{-}144h)}$ differ from Mo-GMCSF$^{IL4(72\text{-}144h)}$ monocyte-derived cells. (A) Workflow for Figure 4 and S4. (**B**) Principal component analysis (PCAs) based on 18,857 present probes. Principal components (PCs) 2 versus 3 are shown, which account for 19.2% versus 10.6% of the total variance, respectively. (**C**) Heatmap visualizing pearson correlation values (pearson correlation coefficient matrix, PCCM) calculated pairwise between all cell types on the basis of the top 1000 most variable genes. The color scaling ranges from white (low correlation) via yellow to red (high correlation). **(D-E)** Histograms show relative expression of (**D**) CD23 and (**E**) CD209 on Mo-GMCSF$^{(72h\text{-}144h)}$ and Mo-GMCSF$^{IL4(0\text{-}144h)}$ analysed by flow cytometry. Bar plots show quantification of the mean fluorescence intensity (MFI) of the (**D**) CD23 and (**E**) CD209 signal on Mo-GMCSF$^{(72h\text{-}144h)}$ or Mo-GMCSF$^{IL4(0\text{-}144h)}$. Representative data shown (n=3, mean + SEM). Statistical significance was determined using paired t-test, with p = 0.415 and p = 0.399 for (**D**) and (**E**), respectively. **(F)** Representative western blot image of MMP12 and β-Actin protein expression in Mo-GMCSF$^{(72h\text{-}144h)}$ or Mo-GMCSF$^{IL4(0\text{-}144h)}$ (n=3). **(G)** Statistical quantification of MMP12 protein expression normalized to β-Actin in Mo-GMCSF$^{(72h\text{-}144h)}$ or Mo-GMCSF$^{IL4(0\text{-}144h)}$ using western blot (n=3, mean + SEM). Statistical significance was determined using paired t-test with p = 0.229**. (H)** Analysis of culture supernatants of Mo-GMCSF$^{(72h\text{-}144h)}$ or Mo-GMCSF$^{IL4(0\text{-}144h)}$ for CCL22 using ELISA (n=6, 3 independent donors, 2 technical replicates)). Data are shown as mean + SEM. Statistical significance was determined using paired t-test with $^{**}p < 0.01$**. (I)** Flow cytometric analysis of Mo-GMCSF$^{(72h\text{-}144h)}$ or Mo-GMCSF$^{IL4(0\text{-}144h)}$ after 4hrs of incubation with YG beads (n=6, mean + SEM). Statistical significance between cell types after 4h of incubation was determined using paired t-test with p = 0.83. (**J**) Quantification of accumulated distance and (**K**) average migratory speed (velocity) of motile myeloid cells corresponding to Figure 4F. Results show one representative experiment out of three. Statistical significance was determined using Student's t-test in (**J**) and Mann-Whitney Rank Sum test in (**K**) with $^{***}p < 0.001$.

Supplementary figure 5

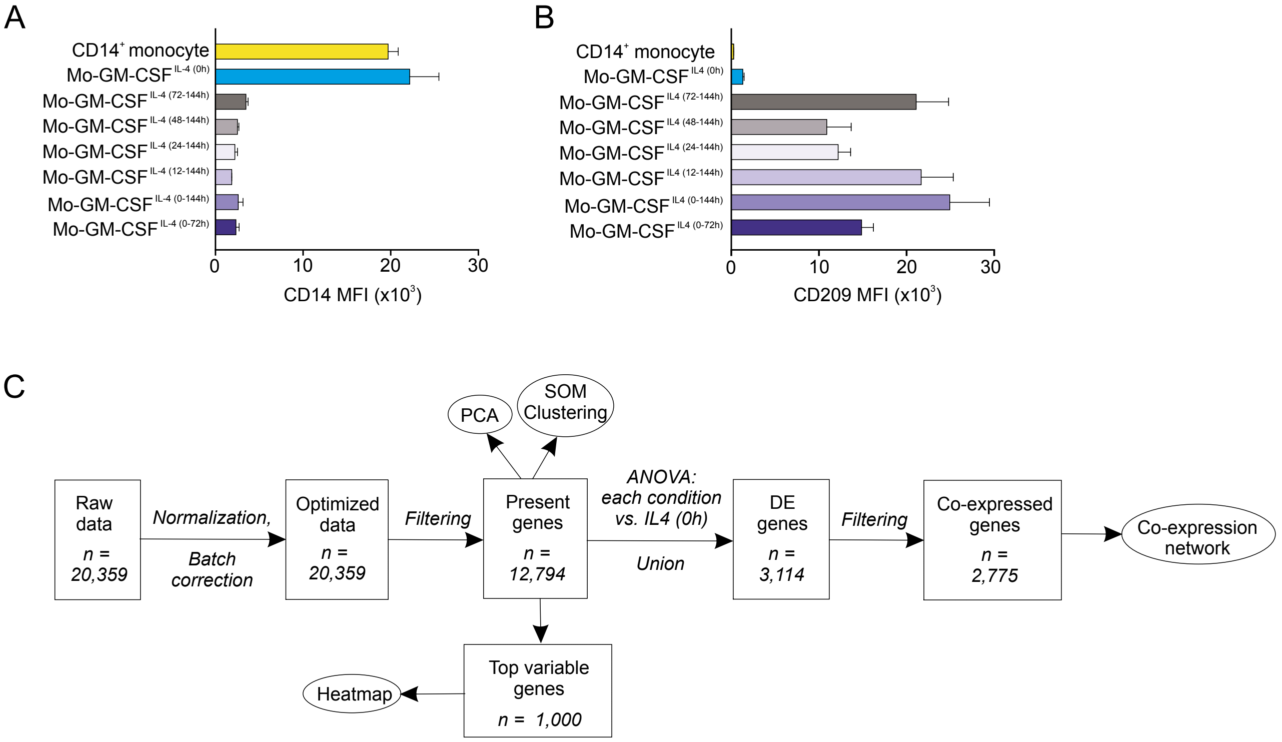

Figure S5 related to Figure 5

Timing of IL4 determines transcriptional regulation in activated monocytes. (A-B) Flow cytometric analysis of the MFI of (**A**) CD14 and (**B**) CD209 on the depicted cell types (n=4). (**C**) Workflow for the RNA-Seq data analysis in Figure 5.

Supplementary figure 6

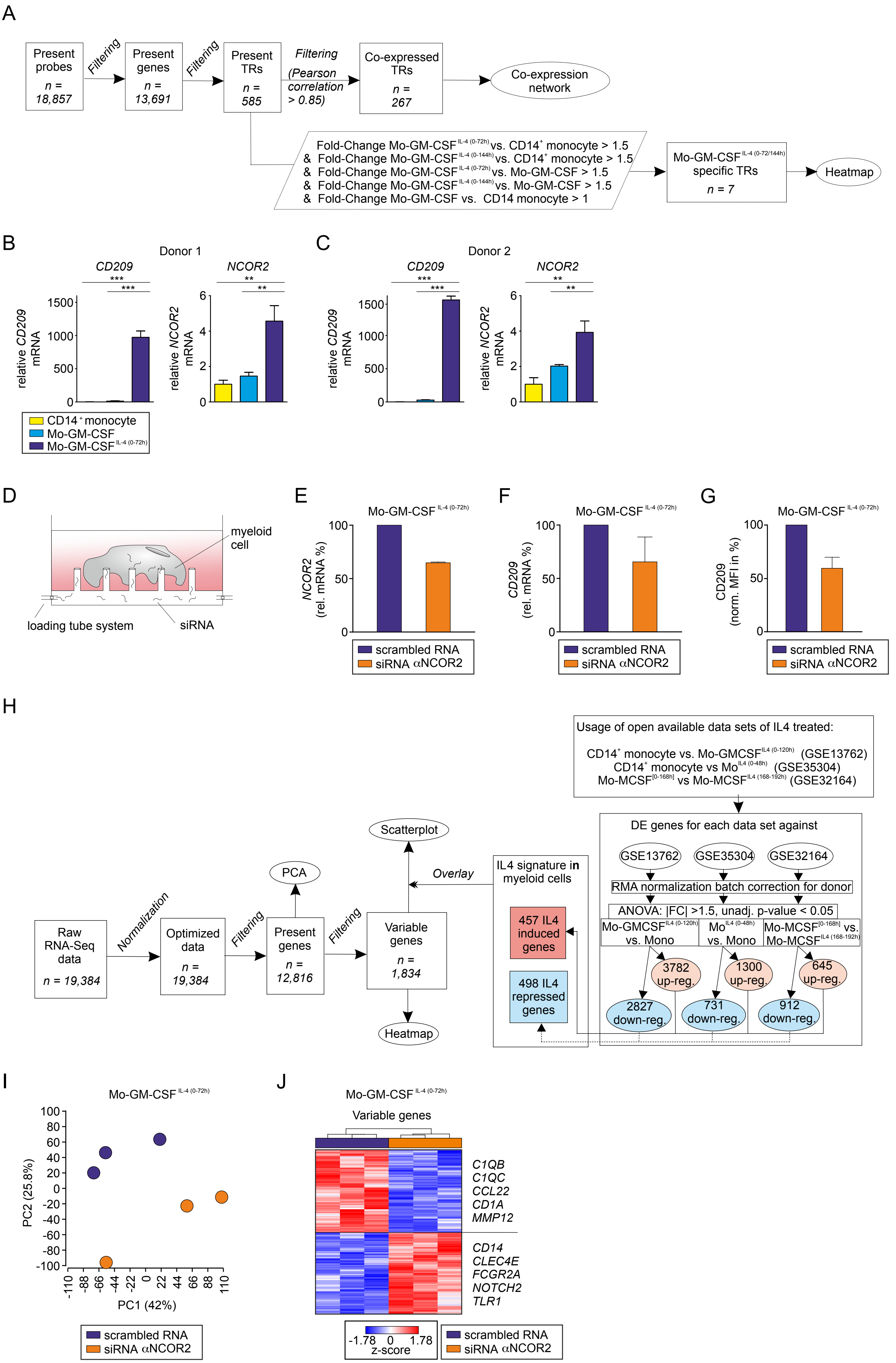

Figure S6 related to Figure 6

NCOR2 is a transcriptional regulator of Mo-GMCSF$^{IL4(0-72/144h)}$. (A) Workflow for Figure 6 and S6. **(B)** *Realtime* PCR measurement of *NCOR2* and *CD209* mRNA levels relative to *GAPDH* mRNA levels in Mo-GMCSF and Mo-GMCSF$^{IL4(0-72h)}$ in donor 1 (mean + standard deviation). **(C)** *Realtime* PCR measurement of *NCOR2* and *CD209* mRNA levels relative to *GAPDH* mRNA levels in Mo-GMCSF and Mo-GMCSF$^{IL4(0-72h)}$ in donor 2 (mean + standard deviation). (**D**) Schema for the experimental setup of the siRNA-based gene knockdown. **(E)** Realtime PCR measurement of *NCOR2* mRNA levels relative to *GAPDH* mRNA levels in Mo-GMCSF$^{IL4(0-72h)}$ (n=2, mean + standard deviation). **(F)** Realtime PCR measurement of *CD209* mRNA levels relative to *β-Actin* mRNA levels in Mo-GMCSF$^{IL4(0-72h)}$ (n = 2, mean + standard deviation). **(G)** Quantification of the normalized MFI of CD209 on Mo-GMCSF$^{IL4(0-72h)}$ (n = 2, mean + standard deviation). (**H**) Workflow for the analysis of the siRNA gene knockdown dataset. (**I**) Principal component analysis (PCA) based on 12,816 present genes. Displayed are principal components (PCs) 1 versus 2, which account for 42% versus 25.8% of the total variance, respectively. (**J**) Heatmap of the top 1000 genes being most variable across the dataset. Rows were ordered based on hierarchical clustering. The expression values were z-transformed and scaled to a minimum of -1.78 (blue) and a maximum of 1.78 (red). A few genes of each group are depicted on the right side next to the heatmap.
