# Supplementary material for: Cellular Differentiation of Human Monocytes Is Regulated by Time-Dependent Interleukin-4 Signaling and the Transcriptional Regulator NCOR2
Source: Immunity. 2017 Dec 19;47(6):1051–1066.e12. doi: 10.1016/j.immuni.2017.11.024 (PMC5772172; doi:10.1016/j.immuni.2017.11.024)
Supplement: Document S2. Article plus Supplemental Information [file mmc10.pdf]

Immunity

Article

Cellular Differentiation of Human Monocytes Is Regulated by Time-Dependent Interleukin-4 Signaling and the Transcriptional Regulator NCOR2

Highlights

- *In vitro* monocyte cultures model *in vivo* inflammatory dendritic cells and macrophages
- Monocyte-derived dendritic cells integrate interleukin-4 signaling time dependently
- NCOR2 controls differentiation of *in vitro* generated monocyte-derived dendritic cells
- *In vitro* generated monocyte-derived cells are phenotypically heterogeneous

Authors

Jil Sander, Susanne V. Schmidt, Branko Cirovic, ..., Florent Ginhoux, Andreas Schlitzer, Joachim L. Schultze

Correspondence

andreas.schlitzer@uni-bonn.de

In Brief

Monocyte-derived cellular derivatives are used clinically and are a crucial tool in basic research. Sander and colleagues now show that they transcriptionally relate to *in vivo* inflammatory monocytes, that they integrate differentiation cues time dependently, and that *in vitro* differentiated monocytes are phenotypically heterogeneous.

Sander et al., 2017, Immunity *47*, 1051–1066
December 19, 2017 © 2018 The Authors. Published by Elsevier Inc.
https://doi.org/10.1016/j.immuni.2017.11.024

Immunity
Article

Cellular Differentiation of Human Monocytes Is Regulated by Time-Dependent Interleukin-4 Signaling and the Transcriptional Regulator NCOR2

Jil Sander,[1,15] Susanne V. Schmidt,[2,15] Branko Cirovic,[3] Naomi McGovern,[4,5] Olympia Papantonopoulou,[1] Anna-Lena Hardt,[1] Anna C. Aschenbrenner,[1] Christoph Kreer,[6] Thomas Quast,[7] Alexander M. Xu,[8] Lisa M. Schmidleithner,[1] Heidi Theis,[1] Lan Do Thi Huong,[1] Hermi Rizal Bin Sumatoh,[4] Mario A.R. Lauterbach,[2] Jonas Schulte-Schrepping,[1] Patrick Günther,[1] Jia Xue,[1] Kevin Baßler,[1] Thomas Ulas,[1] Kathrin Klee,[1] Natalie Katzmarski,[3] Stefanie Herresthal,[1] Wolfgang Krebs,[1] Bianca Martin,[2] Eicke Latz,[2,9,10] Kristian Händler,[1] Michael Kraut,[1] Waldemar Kolanus,[7] Marc Beyer,[1,11] Christine S. Falk,[12] Bettina Wiegmann,[13] Sven Burgdorf,[6] Nicholas A. Melosh,[8] Evan W. Newell,[4] Florent Ginhoux,[4] Andreas Schlitzer,[3,4,15,16,*] and Joachim L. Schultze[1,14,15]

[1]Genomics and Immunoregulation, LIMES-Institute, University of Bonn, 53115 Bonn, Germany
[2]Institute of Innate Immunity, University Hospital Bonn, University of Bonn, Sigmund-Freud-Strasse 25, 53127 Bonn, Germany
[3]Myeloid Cell Biology, LIMES-Institute, University of Bonn, 53115 Bonn, Germany
[4]Agency for Science, Technology and Research (A*STAR), Singapore Immunology Network (SIgN), 138648 Singapore, Singapore
[5]Department of Pathology and Center for Trophoblast Research, University of Cambridge, CB2 1QP Cambridge, UK
[6]Cellular Immunology, LIMES-Institute, University of Bonn, 53115 Bonn, Germany
[7]Molecular Immunology & Cell Biology, LIMES-Institute, University of Bonn, 53115 Bonn, Germany
[8]Department of Materials Science and Engineering, Stanford University, Stanford, CA 94305, USA
[9]Department of Medicine, University of Massachusetts Medical School, Worcester, MA 01605, USA
[10]German Center for Neurodegenerative Diseases, 53127 Bonn, Germany
[11]Molecular Immunology, German Center for Neurodegenerative Diseases (DZNE), Sigmund-Freud-Str. 27, 53127 Bonn, Germany
[12]Institute of Transplant Immunology, Integrated Research and Treatment Center Transplantation, Hannover Medical School, 30625 Hannover, Germany
[13]Department of Cardiothoracic, Transplantation and Vascular Surgery, Hannover Medical School, 30625 Hannover, Germany
[14]Platform for Single Cell Genomics and Epigenomics (PRECISE) at the German Center for Neurodegenerative Diseases and the University of Bonn, 53127 Bonn, Germany
[15]These authors contributed equally
[16]Lead Contact
*Correspondence: andreas.schlitzer@uni-bonn.de
https://doi.org/10.1016/j.immuni.2017.11.024

SUMMARY

Human *in vitro* generated monocyte-derived dendritic cells (moDCs) and macrophages are used clinically, e.g., to induce immunity against cancer. However, their physiological counterparts, ontogeny, transcriptional regulation, and heterogeneity remains largely unknown, hampering their clinical use. High-dimensional techniques were used to elucidate transcriptional, phenotypic, and functional differences between human *in vivo* and *in vitro* generated mononuclear phagocytes to facilitate their full potential in the clinic. We demonstrate that monocytes differentiated by macrophage colony-stimulating factor (M-CSF) or granulocyte macrophage colony-stimulating factor (GM-CSF) resembled *in vivo* inflammatory macrophages, while moDCs resembled *in vivo* inflammatory DCs. Moreover, differentiated monocytes presented with profound transcriptomic, phenotypic, and functional differences. Monocytes integrated GM-CSF and IL-4 stimulation combinatorically and temporally, resulting in a mode- and time-dependent differentiation relying on NCOR2. Finally, moDCs are phenotypically heterogeneous and therefore necessitate the use of high-dimensional phenotyping to open new possibilities for better clinical tailoring of these cellular therapies.

INTRODUCTION

Recently, transcriptomic, epigenetic, functional, and fate-mapping studies established the identity of three mononuclear cell lineages within the myeloid cell network: macrophages (Macs), dendritic cells (DCs), and monocytes (MOs) (Schlitzer et al., 2015a). During murine embryogenesis, progenitors colonize developing tissues differentiating into tissue-resident Macs, which are long lived and self-maintained in most tissues (Ginhoux and Guilliams, 2016). DCs can be separated into plasmacytoid DCs, conventional DC 1 (cDC1s), and cDC2s (Merad et al., 2013). cDC1s and cDC2s arise from specialized precursors within the bone marrow forming their functional specialization during development (Grajales-Reyes et al., 2015; Schlitzer et al., 2015b; See et al., 2017).

The third component of the mononuclear phagocyte (MP) network are MOs. In mice, MOs are divided into Ly6c^{lo} and Ly6c^{hi} MOs (Varol et al., 2015), while in human blood, three

This is an open access article under the CC BY license (http://creativecommons.org/licenses/by/4.0/).

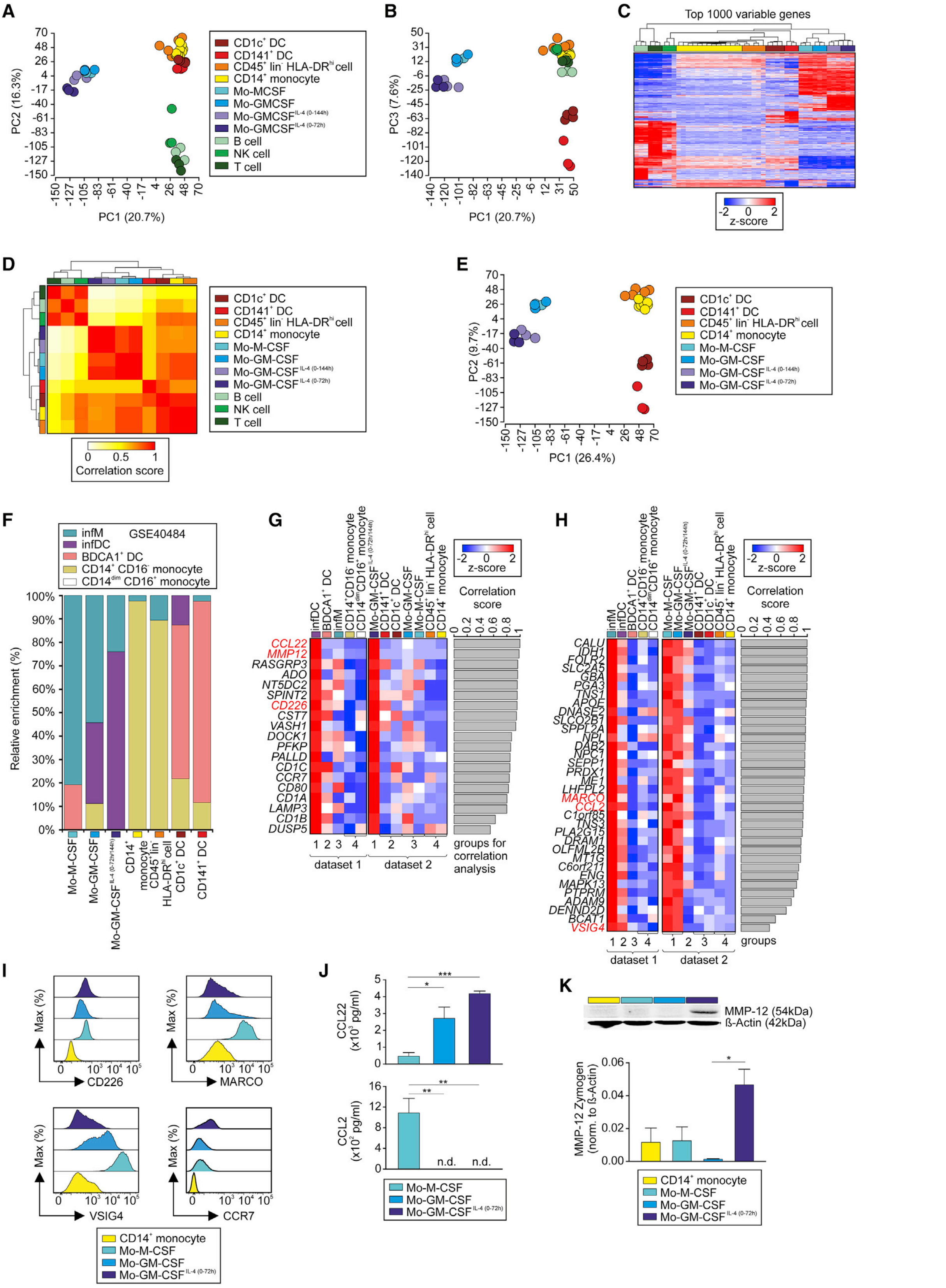

(legend on next page)

different monocyte subsets are identified by expression of CD14 and CD16, in which $CD14^+CD16^-$ MOs correspond to murine $Ly6c^{hi}$ MOs and $CD14^-CD16^+$ MOs to murine $Ly6c^{lo}$ MOs (Auffray et al., 2009). Yet little is known whether this classification relates to functional specialization of distinct subsets.

Prior work suggests functional overlap between DCs, MOs, and Macs, including basic mechanisms of phagocytosis (Fraser et al., 2009), anti-bacterial activity (Fehlings et al., 2012), antigen uptake, processing, and presentation (Cella et al., 1997), the capacity to activate adaptive immune cells (Toujas et al., 1997), cellular motility (Rossi et al., 2011), and activation programs (Frucht et al., 2000). This overlap has proven difficult to parse, but new knowledge concerning the distinct ontogeny of these cells provided the opportunity to reassess the division of labor between DCs, MOs, and tissue Macs.

Currently, assigning functions to subsets of the MP system requires use of simplified *in vitro* systems to focus on basic molecular aspects. Murine granulocyte macrophage colony-stimulating factor (GM-CSF)- or macrophage colony-stimulating factor (M-CSF)-driven bone marrow-derived DCs and Mac cultures are frequently used to elucidate and assign molecular mechanisms of functions to subsets of MPs. However, these *in vitro* cultures create heterogeneous cultures, making attribution of distinct cellular functions difficult (Helft et al., 2015). This conundrum highlights the need for a detailed investigation of cellular identity and the regulation thereof in such *in vitro* cultures (Xue et al., 2014).

Sallusto and Lanzavecchia (1994) described the *in vitro* generation of human MO-derived DCs (moDCs) by culturing peripheral blood MOs with GM-CSF and IL-4. Here, the term moDCs has been attributed to an activated MO population with DC-like function based on morphological and functional criteria. Similar functionally converging phenotypes are observed in human *in vitro* systems of MO-derived M-CSF-driven Macs (moMacs) (Akagawa et al., 2006) or GM-CSF (Xue et al., 2014). Systems biology-based definitions of MP function and nomenclature have been established, yielding insights about identity, regulation, and developmental origin of those cells (Xue et al., 2014). However, studies directly addressing their relationships to MPs observed *in vivo* remain limited (Ohradanova-Repic et al., 2016). Understanding such relationships and linking this knowledge to cellular ontogeny is crucial considering the interest in using *in vitro* generated MPs for immunotherapy (Brower, 2005; Garg et al., 2017). Therefore, the functional convergence, plasticity, and heterogeneity of MO-derived MPs paired with the clinical interest raises several important questions. What are the *in vivo* counterparts of *in vitro* MO derivatives? Do MOs integrate cytokine signaling in a temporal fashion and how is it regulated molecularly? Lastly, how heterogeneous are human *in vitro* MO cultures?

Computational analysis of MP transcriptomes and analysis of cellular phenotype, function, and perturbation experiments elucidated the relationship of human moDCs and moMacs to the *in vivo* MP system. The differentiation of MO *in vitro* culture systems is multifaceted, integrating time-dependent signals delivered by GM-CSF and IL-4 and orchestrated by nuclear receptor corepressor 2 (NCOR2). Finally, mass cytometry (MC) revealed cellular heterogeneity of moDCs with several subsets being identified. These results uncover the *in vivo* counterparts of MO derivatives and identify a novel regulator of MO differentiation and plasticity.

RESULTS

In Vitro Differentiated Human MO-Derived MPs Are Transcriptionally Similar to MO-Derived Inflammatory MPs

Human MOs differentiated with M-CSF are used as models for human Macs (Akagawa et al., 2006), whereas MOs differentiated with GM-CSF and IL-4 are models for human DCs (Sallusto and Lanzavecchia, 1994). For clarity and in light of recent findings concerning DC, MO, and Mac ontogeny (Guilliams and van de Laar, 2015), we term differentiated MOs according to their activation, e.g., MOs activated with M-CSF are named MOs-M-CSF and MOs differentiated for a specified duration (0–72 hr; 0–144 hr) with GM-CSF and IL-4 are MOs-GM-CSF^{IL-4}. To establish transcriptional similarity between *ex vivo* isolated cells and differentiated MOs, we compared blood $CD14^+$ MOs, $CD1c^+$ DCs, $CD141^+$ DCs (Haniffa et al., 2012), and T, B, and NK cells alongside $CD45^+lin^-HLA\text{-}DR^{hi}$ lung derived cells, to MOs-M-CSF, MOs-GM-CSF, MOs-GM-$CSF^{IL\text{-}4(0\text{-}72h)}$, and MOs-GM-$CSF^{IL\text{-}4(0\text{-}144h)}$ (Figure S1A).

Principle component analysis (PCA) revealed that T, B, and NK cells formed one of three clusters (Figures 1A and 1B, green), all

Figure 1. Relationship of *In Vitro* Activated Monocyte-Derived Cells

(A and B) PCA (21,250 present probes); displayed principal components (PCs): (A) 1 versus 2 and (B) 1 versus 3.

(C) Heatmap of 1,000 most variable genes in dataset. Log_2-expression values, z-transformed, scaled (−2 [blue] to 2 [red]).

(D) Heatmap, Pearson correlation values (PCV) calculated pairwise between all cell types (top 1,000 most variable genes).

(E) PCA (23,952 present probes).

(F) Relative fractions of MO, BDCA1+ DC, infM, and infDC gene signatures in $CD14^+$ MOs, different MO-derived cells, DCs.

(G and H) Heatmaps of genes specifically expressed in (G) infDCs compared to infMs, $BDCA1^+$ DCs, MOs (dataset 1), and MOs-GM-$CSF^{IL\text{-}4(0\text{-}72h)}$ versus MO-derived cells, $CD14^+$ MOs, and DCs (dataset 2), or in (H) infMs compared to infDCs, $BDCA1^+$ DCs, and MOs (dataset 1), and in MOs-GM-CSF and MOs-M-CSF versus MOs-GM-$CSF^{IL\text{-}4(0\text{-}72h)}$, $CD14^+$ MOs, and DCs (dataset 2). PCVs between indicated group patterns of dataset 1 versus dataset 2, barplot next to heatmaps (correlation cutoff > 0.4). Genes analyzed in (J) and (K) highlighted in red. Log_2-expression values, z-transformed, scaled (−2 [blue] to 2 [red]).

(I) Histograms (flow cytometry analysis), relative expression CD226, MARCO, VSIG4, and CCR7 (representative data, n = 4).

(J) Analysis of cell culture supernatants of MOs-M-CSF, MOs-GM-CSF, and MOs-GM-$CSF^{IL\text{-}4(0\text{-}72h)}$ for CCL22 and CCL2 using ELISA (n = 3, 2 technical replicates each, mean + SEM, one-way RM [repeated-measures] ANOVA, Tukey's method for multiple test correction, with *$p < 0.05$, **$p < 0.01$, and ***$p < 0.001$; n.d., not detected).

(K) MMP12 quantification (relative) in $CD14^+$ MOs, MOs-M-CSF, MOs-GM-CSF, and MOs-GM-$CSF^{IL\text{-}4(0\text{-}72h)}$ by immunoblot (n = 3, mean + SEM, one-way ANOVA and Tukey's method for multiple test correction, with *$p < 0.05$).

Please also see Figure S1.

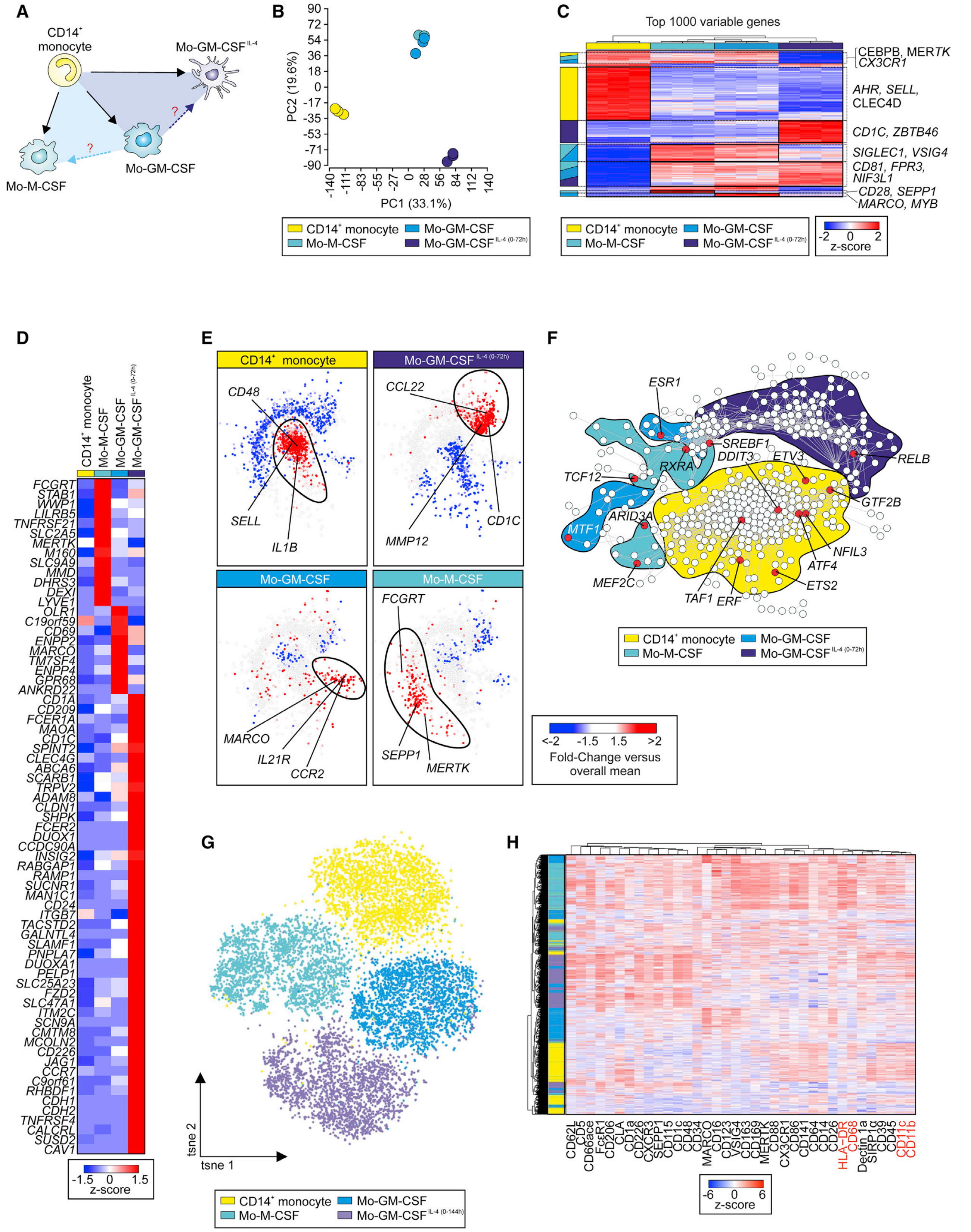

(legend on next page)

ex vivo isolated MPs formed a second cluster (red and yellow), and both these clusters were most distinct from a third cluster containing *ex vivo* polarized MO-derived MPs (blue, purple, cyan, lilac). This three-cluster structure was validated by hierarchical clustering (HC) of the 1,000 most variable genes (Figure 1C; Table S1) and Pearson correlation coefficient matrix (PCCM) analysis (Figure 1D), showing that *in vitro* differentiated MOs are transcriptionally unique compared to *ex vivo* human peripheral blood cells.

To analyze MP subsets, T, B, and NK cells were excluded from the previous dataset and remaining cell types were analyzed by PCA (Figures 1E, S1B, and S1C). *In vitro* generated cells clustered separately compared to *in vivo* MPs, with lung-derived $CD45^{+}lin^{-}HLA\text{-}DR^{hi}$ and $CD14^{+}$ MOs forming a separate cluster away from a DC cluster and a cluster for MOs-M-CSF, MOs-GM-CSF, MOs-GM-$CSF^{IL\text{-}4(0\text{-}144h)}$, and MOs-GM-$CSF^{IL\text{-}4(0\text{-}72h)}$. Additionally, *in vitro* MO-derived MPs shared a common set of differentially expressed genes (DEGs) in comparison to *ex vivo* $CD14^{+}$ MOs (Figures S1D and S1E; Table S1), supporting transcriptional difference to homeostatic cells. These findings raised the question of which cells found *in vivo* represent the MO model systems.

We assessed whether the *in vitro* MO-derived MPs reflected human MO-derived inflammatory DCs (infDCs) and inflammatory Mac (infMs) *in vivo* utilizing a published dataset (Segura et al., 2013). We generated a signature matrix representing infDCs, infMs, $BDCA1^{+}$ (CD1c) DCs, $CD14^{+}CD16^{-}$ MOs, and $CD14^{dim}CD16^{+}$ MOs and assessed the relative signature enrichment in our dataset using linear support vector regression (Figure 1F; Table S1; Newman et al., 2015). MOs-M-CSF showed highest enrichment of infM genes, while infDC genes were highly enriched in MOs-GM-$CSF^{IL\text{-}4}$, suggesting that *in vitro* polarization conditions reflected *in vivo* infDCs and infMs as shown for the mouse (Helft et al., 2015). Control gene sets derived from $CD14^{+}CD16^{-}$ MOs were most highly enriched in *ex vivo* $CD14^{+}$ MOs and lung-derived $CD45^{+}lin^{-}HLA\text{-}DR^{hi}$ cells, while the $BDCA1^{+}$ DC signature enriched in both *ex vivo* myeloid DC subsets. Gene set enrichment analyses (GSEA) confirmed transcriptional similarities between infMs and MOs-M-CSF but also MOs-GM-CSF and between infDCs and MOs-GM-$CSF^{IL\text{-}4}$ (Figure S1F). Collectively, we defined four groups in both datasets, describing comparable cell subsets (see STAR Methods). Then, we performed PCCM by comparing expression patterns in both datasets based on the four groups and visualized genes with highest correlation scores between infDCs and MOs-GM-$CSF^{IL\text{-}4(0\text{-}72h/144h)}$ (Figure 1G; Table S1) and between infMs and both MOs-M-CSF and MOs-GM-CSF (Figure 1H; Table S1), which included several surface markers and secreted molecules (Figures S1G–S1J; Table S1). Many genes associated with activated DCs (*CCL22*, *MMP12*, *CD226*, *CCR7*) were highly elevated in both infDCs and MOs-GM-$CSF^{IL\text{-}4}$ (Figure 1G), while typical Mac genes (*MARCO*, *CCL2*, *VSIG4*) were most highly expressed in infMs, MOs-M-CSF, and MOs-GM-CSF (Figure 1H). Furthermore, differential regulation of the proteins CD226, MARCO, VSIG4, CCR7, CCL2, CCL22, and MMP12 was validated (Figures 1I–1K). Collectively, polarization of MOs *in vivo* (infDCs, infMs) (Segura et al., 2013) and *in vitro* leads to similar transcriptomic identities, including cell surface and effector molecules, allowing us to use these models to understand the role of M-CSF, GM-CSF, and IL-4 for inflammation-associated MO differentiation.

GM-CSF + IL-4 but Not GM-CSF or M-CSF Alone Enforce a Unique Transcriptional Signature in Human $CD14^{+}$ MOs

Next, we wanted to understand the similarities and differences in MO activation induced by M-CSF, GM-CSF, and IL-4. Previous work suggested important differences between MOs-M-CSF and MOs-GM-CSF (Lacey et al., 2012). However, these studies did not answer the overall relationship between all three activation conditions (Figure 2A). Using the well-established surface markers CD14, CD11b, and CD209, we assessed the differences between MOs-M-CSF, MOs-GM-CSF, and MOs-GM-$CSF^{IL\text{-}4(0\text{-}72h)}$ (Figure S2A). This revealed that CD14 marked MOs, MOs-M-CSF, and MOs-GM-CSF, but not MOs-GM-$CSF^{IL\text{-}4(0\text{-}72h)}$ (Sallusto and Lanzavecchia, 1994). CD209 was exclusively expressed by MOs-GM-$CSF^{IL\text{-}4(0\text{-}72h)}$. CD11b did not discriminate between the cell populations. However, when reassessing the overall relationship between *ex vivo* isolated $CD14^{+}$ MOs, MOs-M-CSF, MOs-GM-CSF, and MOs-GM-$CSF^{IL\text{-}4(0\text{-}72h)}$ on transcriptome level (Figure S2B), MOs-M-CSF and MOs-GM-CSF clustered together, while MOs and MOs-GM-$CSF^{IL\text{-}4(0\text{-}72h)}$ clustered separately as demonstrated by PCA (Figure 2B), PCCM (Figure S2C), and HC (Figure 2C;

Figure 2. MOs-GM-$CSF^{IL\text{-}4}$ Are Most Distinct from MOs-M-CSF and MOs-GM-CSF

(A) Schema describing the questions addressed here and in Figure S2.

(B) PCA (18,318 present probes).

(C) Heatmap of 1,000 most variable genes in dataset. Log_2-expression values, z-transformed, scaled (−2 [blue] to 2 [red]).

(D) Heatmap of specifically expressed genes in a single out of the three MO-derived cells versus $CD14^{+}$ MOs and the other two MO-derived cell types. Log_2-expression values, z-transformed, scaled (−1.5 [blue] to 1.5 [red]).

(E) Co-expression networks (union of 2,086 DEG [fold-change > 2 or < −2 and FDR-adjusted p value < 0.05]) between each of three MO-derived cells types versus $CD14^{+}$ MOs. Fold-change of respective cell type versus overall mean mapped onto networks and displayed blue (negative fold-change) over white to red (positive fold-change). Based on fold-change patterns, networks were divided into four clusters, each cluster representing one of four cell types.

(F) Co-expression network (411 TRs expressed in dataset). For each cell type, fold-change of respective cell type versus overall mean mapped onto network. Cell type-specific clusters of upregulated regulators were generated, indicated by color-coded shadings behind network. TRs highlighted in red were predicted as unique master regulators of corresponding cell type. Prediction performed on all genes highlighted in red (fold-change > 1.5 over overall mean) in corresponding cell type-specific cluster in (E).

(G) *t*-SNE display of $CD14^{+}$ MOs, MOs-M-CSF, MOs-GM-CSF, and MOs-GM-$CSF^{IL\text{-}4(0\text{-}144h)}$ analyzed by MC (n = 3).

(H) Heatmap and HC of mean surface marker expression analyzed using MC. Normalized intensity values, z-transformed, scaled (−6 [blue] to 6 [red]). Color code depicts cluster assignment according to culture condition. Color code as in (G).

Please also see Figure S2.

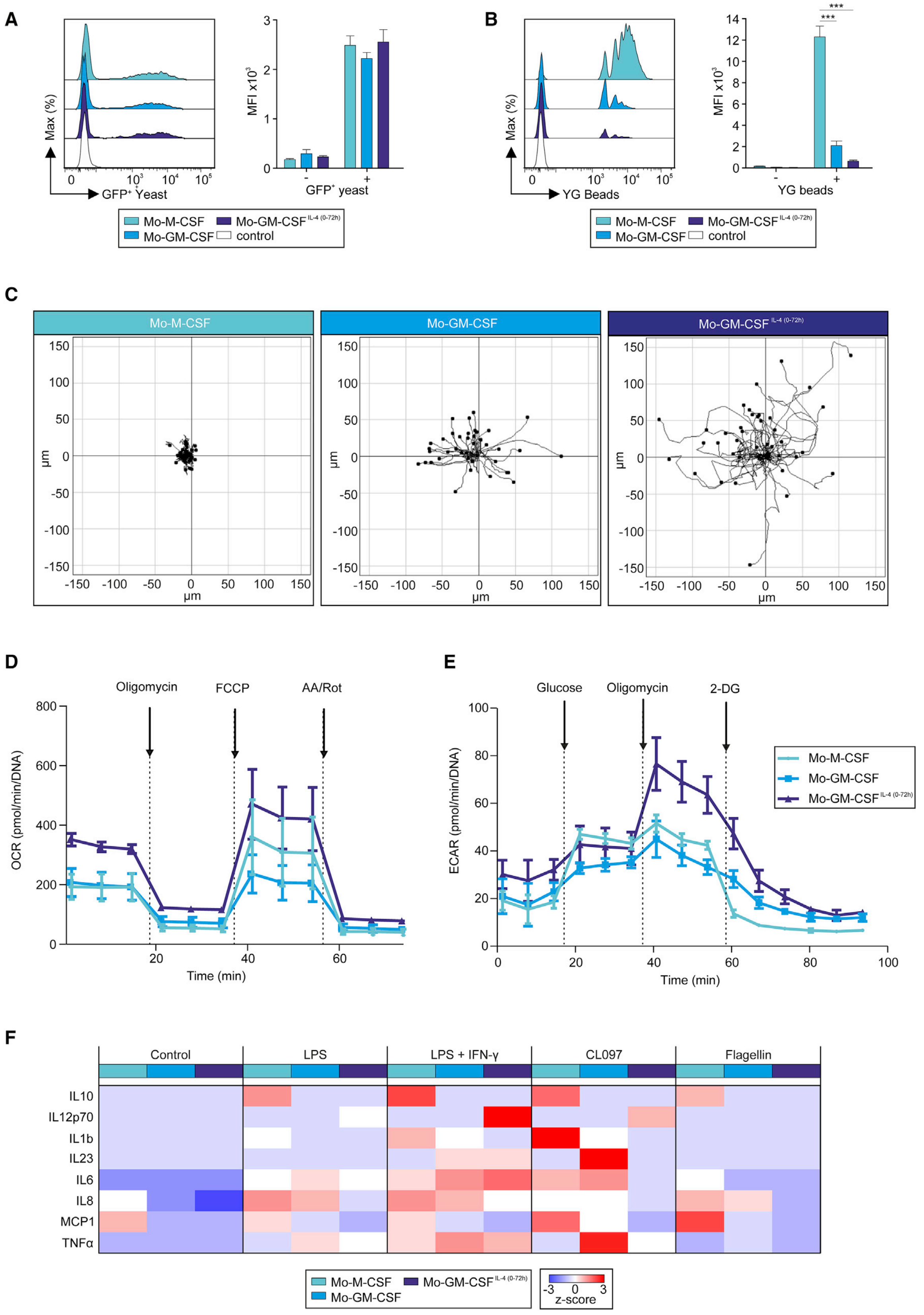

(legend on next page)

Table S2). MOs-M-CSF and MOs-GM-CSF were marked by high expression of Mac genes (*CD81*, *VSIG4*, *SIGLEC1*, *MARCO*, *FPR3*) while MOs and MOs-GM-CSF$^{IL\text{-}4(0\text{-}72h)}$ cell populations formed separated gene clusters marked by expression of MO-associated (*AHR*, *SELL*, *CLEC4D*) or DC-associated (*CD1C*, *ZBTB46*) genes, respectively. Gene-level analysis of the present surfaceome (see STAR Methods) of *ex vivo* isolated CD14$^+$ MOs, MOs-M-CSF, MOs-GM-CSF, and MOs-GM-CSF$^{IL\text{-}4(0\text{-}72h)}$ revealed only a small number of DEGs for MOs-M-CSF and MOs-GM-CSF but a markedly different expression profile of surface markers related to MOs-GM-CSF$^{IL\text{-}4(0\text{-}72h)}$ (Figure 2D). Additionally, profiling the expression of pattern recognition receptors (PRRs) on these cell populations revealed clear differences (Figure S2D). MOs-M-CSF, MOs-GM-CSF, and MOs-GM-CSF$^{IL\text{-}4(0\text{-}72h)}$ decreased expression of components of the inflammasome signaling complex (*CASP1*, *NLRP1*, *2*, and *3*) but increased expression of the intracellular PRR *NOD1*. *NOD2* expression was maintained only by MOs-GM-CSF. MOs-GM-CSF$^{IL\text{-}4\ (0\text{-}72h)}$ displayed a unique set of PRRs (high *CD209* and *CLEC10A*, loss of Toll-like receptor [TLR] 7 and 5). To determine transcriptional differences, we performed co-expression network analysis based on the union of DEGs between MOs and the three differentiation conditions and mapped differential gene expression onto the network (Figure 2E; Table S2). Within the network topology, a central large MO-related gene cluster was surrounded by separate clusters for each of the three differentiation conditions, indicating that despite an overall close relationship, MOs-M-CSF and MOs-GM-CSF are characterized by signal-specific subclusters of regulated genes (Figure 2E). Identification of DEGs between MOs and MOs-M-CSF and MOs-GM-CSF further supported a close overall relationship, but also indicated differently regulated genes in only one or the other condition (Figures S2E and S2F; Table S2). Gene ontology enrichment analysis (GOEA) revealed that enriched terms in MOs-GM-CSF are associated with immune response and regulation of protein metabolism, whereas most GO terms enriched in MOs-M-CSF relate to metabolism and G-protein-coupled receptor signaling (Figure S2G; Table S2).

To identify the transcriptional regulators (TRs) involved in MO differentiation, we predicted upstream transcription factors for each of the four condition-specific clusters identified in Figure 2E and generated a co-expression network of TRs expressed in the dataset to identify specific clusters of upregulated TRs for CD14$^+$ MOs (yellow), MOs-M-CSF (turquoise), MOs-GM-CSF (light blue), and MOs-GM-CSF$^{IL\text{-}4(0\text{-}72h)}$ (dark blue) (Figure 2F; Table S2). Finally, we mapped the predicted master transcription factors onto the co-expression network and identified *NFIL3*, *ATF4*, and *ETS2* among others to be putative regulators of CD14$^+$ MOs. *TCF12*, *MEF2C*, and *ARID3A* were predicted to specifically regulate MOs-M-CSF, whereas *ESR1*, *MTF1*, and *SREBF1* were anticipated to regulate MOs-GM-CSF identity. *RELB*, implicated as important for mouse DC differentiation (Wu et al., 1998), was predicted as a central regulator of the transcriptional identity of MOs-GM-CSF$^{IL\text{-}4(0\text{-}72h)}$, highlighting the uniqueness of the transcriptional identity induced by GM-CSF and IL-4.

Since traditional surface markers (CD14, CD11b, CD209) are not informative to discriminate *in vitro* polarized MO subsets, we designed a comprehensive MC panel incorporating markers defined by our transcriptomic approach and compared *ex vivo* isolated blood CD14$^+$ MOs to *in vitro* M-CSF-, GM-CSF-, and GM-CSF + IL-4-polarized CD14$^+$ MOs. Dimensionality reduction using *t*-distributed neighbor embedding (*t*-SNE) (Maaten and Hinton, 2008) of the CD45$^+$lin$^-$HLA-DR$^+$ cell fraction comparing CD14$^+$ MOs, MOs-M-CSF, MOs-GM-CSF, and MOs-GM-CSF$^{IL\text{-}4(0\text{-}144h)}$ revealed donor-independent separation into four cellular clusters (Figures 2G, 2H, and S2H; Table S7). Overlaying their differentiation history on the *t*-SNE topology shows that the identified clusters corresponded to the four differentiation cues used, validating their transcriptomic differences (Figure 2B). Widely used markers for the delineation of MOs, Macs, and DCs, such as CD11b, CD68, CD11c, and HLA-DR were expressed uniformly across all four clusters, showing that only a high-dimensional phenotyping approach enables robust detection of polarized subsets across all four differentiation conditions (Figure 2H). CD14$^+$ MOs showed a high expression of CLA and CD64, whereas MOs-GM-CSF displayed a high expression of MARCO. VSIG4 was expressed by MOs-GM-CSF and MOs-M-CSF, whereas MOs-M-CSF expressed high amounts of CD163, CD169, and MERTK. The MOs-GM-CSF$^{IL\text{-}4(0\text{-}144h)}$ cluster specifically expressed SEPP1, FcεR1, CD1c, and CD48. Taken together, MC enabled us to identify transcriptionally validated markers, facilitating separation of different transcriptomic entities on the protein level.

In Vitro Differentiated MO Subsets Are Functionally and Metabolically Different

To understand how the transcriptomic and phenotypic differences between the Mo-derived cells influence their ability to phagocytize and to secrete cytokines in response to PRR stimulation, their motility, and their metabolic profile, we first measured receptor-mediated uptake of GFP-labeled yeast or YG beads. MOs-M-CSF, MOs-GM-CSF, and MOs-GM-CSF$^{IL\text{-}4(0\text{-}72h)}$ phagocytosed GFP$^+$ yeast buds after 1 hr of incubation, similarly indicating no differential induction of receptors and signaling pathways involved in yeast uptake (Figures 3A and S3A). MOs-M-CSF displayed an up to 12 times enhanced uptake of YG beads in comparison to MOs-GM-CSF$^{IL\text{-}4(0\text{-}72h)}$ (Figures 3B and S3B), strongly suggesting that M-CSF but not GM-CSF drives phagocytic capacity in MOs. When assessing

Figure 3. Prediction of Differentiated MO Functionality

(A and B) Flow cytometry analysis of MOs-M-CSF, MOs-GM-CSF, and MOs-GM-CSF$^{IL\text{-}4(0\text{-}72h)}$ after incubation with (A) GFP-expressing yeast (1 hr, histogram: representative result [n = 3], bar plot [n = 3], mean + SEM, one-way RM ANOVA, with $p > 0.05$) or (B) YG beads (4 hr, n = 5–6, mean + SEM, one-way RM ANOVA and Tukey's method for multiple test correction, with ***$p < 0.001$).

(C) Migration tracks (3 hr) of MOs-M-CSF, MOs-GM-CSF, and MOs-GM-CSF$^{IL\text{-}4(0\text{-}72h)}$ (representative result, n = 3).

(D and E) OCR (D) and ECAR (E) of MOs-M-CSF, MOs-GM-CSF, and MOs-GM-CSF$^{IL\text{-}4(0\text{-}72h)}$.

(F) Heatmap of mean secreted cytokine concentrations (n = 4). Expression values, z-transformed, scaled (–3 [blue] to 3 [red], raw data, Table S3).

Please also see Figure S3.

cell motility (Figures 3C, S3C, and S3D), MOs-M-CSF, MOs-GM-CSF, and MOs-GM-CSF$^{IL-4(0-72h)}$ showed very little, intermediate, and high motility, respectively, as assessed by distance and velocity analysis suggesting that migratory capacity was linked to GM-CSF and further potentiated by IL-4 signaling (Figures 3C, S3C, and S3D). Metabolically, MOs-M-CSF and MOs-GM-CSF showed a similar rate of oxidative phosphorylation (OXPHOS), extracellular acidification (ECAR), ATP production, and glycolytic capacity (Figures 3D, 3E, and S3E–S3I). MOs-GM-CSF$^{IL-4(0-72h)}$ displayed an increase of OXPHOS, ATP production, and glycolytic capacity alongside an elevated maximal respiration capacity, indicative of increased energetic fitness paired with higher metabolic flexibility induced by IL-4-specific signaling (Figures 3D, 3E, and S3E–S3I).

Linking these phagocytosis, migratory, and metabolic data back to their transcriptomes, we identified key genes involved in the regulation of these processes. *RAB10* (Cardoso et al., 2010), *MSR1* (Bonilla et al., 2013), and *DAB2* (Tumbarello et al., 2013) were implicated as crucial regulators of phagocytosis in immune cells. These genes alongside other regulators of phagocytosis (*RAPH1*, *RILPL2*, *TNS1*, *SCARB2*) were markedly upregulated in MOs-M-CSF (Figure S3J; Table S3). Essential migration and cell motility molecules (Lymphotoxin β [*LTB*] [Yu et al., 2002], *CCL13* [Stellato et al., 1997], *CCL22* [Godiska et al., 1997], *ASAP1* [Curtis et al., 2015]) were upregulated in MOs-GM-CSF$^{IL-4(0-72h)}$ (Figure S3K; Table S3). Genes regulating glycolysis (*PFKL*, *PFKP*) were highly upregulated in MOs-GM-CSF$^{IL-4(0-72h)}$ corresponding to the higher glycolytic capacity shown before (Figure S3L; Table S3). Furthermore, *UQCRC1*, *SDHA*, *ATP5D*, *COX10*, and *ATP5I*, genes of the respiratory chain and *IDH3G*, a molecule involved in the TCA cycle were highly upregulated in MOs-GM-CSF$^{IL-4(0-72h)}$, further linking the transcriptomic and functional level.

Finally, we stimulated Mo-derived cells with LPS, LPS + interferon-γ (IFN-γ), CL097, or Flagellin and measured cytokine release in response to PRR ligation (Figure 3F; Table S3). IL-10, IL-1B, and MCP1 were secreted only by MOs-M-CSF upon activation with either of the four stimuli, demonstrating their similarities to *in vivo* infMs. Conversely, IL-12p70 was secreted only by MOs-GM-CSF$^{IL-4(0-72h)}$ upon LPS, LPS + IFN-γ, and CL097 stimulation, indicating a functional overlap with DCs regarding the induction of T helper 1 (Th1) cell responses. IL-23, a major inflammatory driver essential for Th17 T cell induction was produced only by cells differentiated by GM-CSF. Both MOs-GM-CSF and MOs-GM-CSF$^{IL-4(0-72h)}$ produced IL-23 following LPS + IFN-γ and CL097 stimulation, respectively. This is in line with their similarity to infDCs (Segura et al., 2013).

IL-4 Regulates Transcriptomic and Functional Polarization of moDCs and MO-Derived "M2-like" Macs

Presuming that IL-4 induced a functional and phenotypic convergence of MOs and DCs, we next asked whether MOs-GM-CSF$^{IL-4(0-144h)}$ were distinct from what was previously described as M2 Macs, better described as MO-derived Macs further activated by IL-4, termed here MOs-GM-CSF$^{IL-4(72-144h)}$. To reduce variables to a minimum, we generated MOs-GM-CSF$^{IL-4(72-144h)}$ with GM-CSF, so that the only differences to MOs-GM-CSF$^{IL-4(0-144h)}$ cells were the onset and duration of IL-4 exposure. As controls, MOs polarized for only 3 days with either GM-CSF (MOs-GM-CSF$^{IL-4(0h)}$) or GM-CSF+IL-4 (MOs-GM-CSF$^{IL-4(0-72h)}$) were included (Figure 4A). PCA determined that MOs-GM-CSF$^{IL-4(72-144h)}$ were surprisingly distinct from MOs-GM-CSF$^{IL-4(0-72h),\ (0-144h)}$ (Figures 4B, S4A, and S4B). Co-expression network analysis (Figure 4C), HC using the most variable genes (Figure 4D; Table S4), and PCCM analysis (Figure S4C) supported these results. Surprisingly, CD23 (Figure S4D)—a marker formerly associated with MOs-GM-CSF$^{IL-4(72-144h)}$ (Mantovani et al., 2002)—and CD209 (Figure S4E)—which has been linked to MOs with DC functionality (Geijtenbeek et al., 2000)—were expressed similarly between MOs-GM-CSF$^{IL-4(72-144h)}$ and MOs-GM-CSF$^{IL-4(0-72h),\ (0-144h)}$. No difference in the expression of MMP-12 (Figures S4F and S4G) nor in the release of CCL22 (Figure S4H) were detected. In contrast, MOs-GM-CSF$^{IL-4(0-144h)}$ took up more yeast within 60 min of exposure time (Figure 4E) but phagocytosed similar amounts of YG bead (Figure S4I) when compared to MOs-GM-CSF$^{IL-4(72-144h)}$. MOs-GM-CSF$^{IL-4\ (0-144h)}$ were more motile than MOs-GM-CSF$^{IL-4(72-144h)}$ (Figure 4F), with significantly higher accumulated distance (Figure S4J) and velocity (Figure S4K). Investigation of metabolic parameters of MOs-GM-CSF$^{IL-4(0-144h)}$ and MOs-GM-CSF$^{IL-4(72-144h)}$ revealed no differences in their rate of OXPHOS, ATP production, and glycolysis (data not shown). Collectively, this strongly suggest that the IL-4 signal integrates in a time-dependent manner representing a critical checkpoint for MO differentiation.

Timing of IL-4 Stimulation Determines Transcriptional Regulation MOs-GM-CSF^{IL-4}

Differences between MOs-GM-CSF$^{IL-4(0-144h)}$ and MOs-GM-CSF$^{IL-4(72-144h)}$ could be explained either by a dichotomous model with MOs differentiating into MOs with DC or Mac functionality, or a continuum model that integrates time of exposure suggesting plasticity of MO-derived cells. To determine the best-fitting model, we performed a time kinetics experiment, by adding IL-4 at the start of the culture (Mo-GM-CSF$^{IL-4(0-144h)}$) or at 12 (MOs-GM-CSF$^{IL-4(12-144h)}$), 24 (MOs-GM-CSF$^{IL-4(24-144h)}$), 48 (MOs-GM-CSF$^{IL-4(48-144h)}$), or 72 (MOs-GM-CSF$^{IL-4(72-144h)}$) hr after initiation of differentiation with GM-CSF (Figure 5A). Transcriptomes were assessed after 144 hr and MOs-GM-CSF$^{IL-4(0h)}$ and MOs-GM-CSF$^{IL-4(0-72h)}$ were used as controls. Assessing CD14 (Figures 5B and S5A) and CD209 (Figures 5C and S5B) expression, we observed a dichotomous distribution for cells differentiated with IL-4 being CD14^{lo}CD209^{hi} while cells differentiated only by GM-CSF were CD14^{+}CD209$^{-/lo}$. In contrast, transcriptomic analysis revealed a different model (Figure S5C). PCA (Figure 5D), HC (Figure 5E; Table S5), and self-organizing map (SOM) clustering (Figure 5E) revealed a gradual ordering of samples corresponding to the exposure time to IL-4 indicating a differentiation continuum. Mapping gene expression information for each time point onto a co-expression network (Figure 5F; Table S5) revealed a dense network with two major clusters, one characterized by genes elevated in MOs-GM-CSF$^{IL-4(0)}$ (0 hr, red: upregulated; blue: downregulated), the other one defined by IL-4 exposure (Figure 5G). Adjacent time points showed partially overlapping gene sets suggesting a plastic continuum integrating IL-4 signaling over time, arguing against the dichotomous model of polarization. Collectively, these data suggest that IL-4 signaling differentiates MOs along a

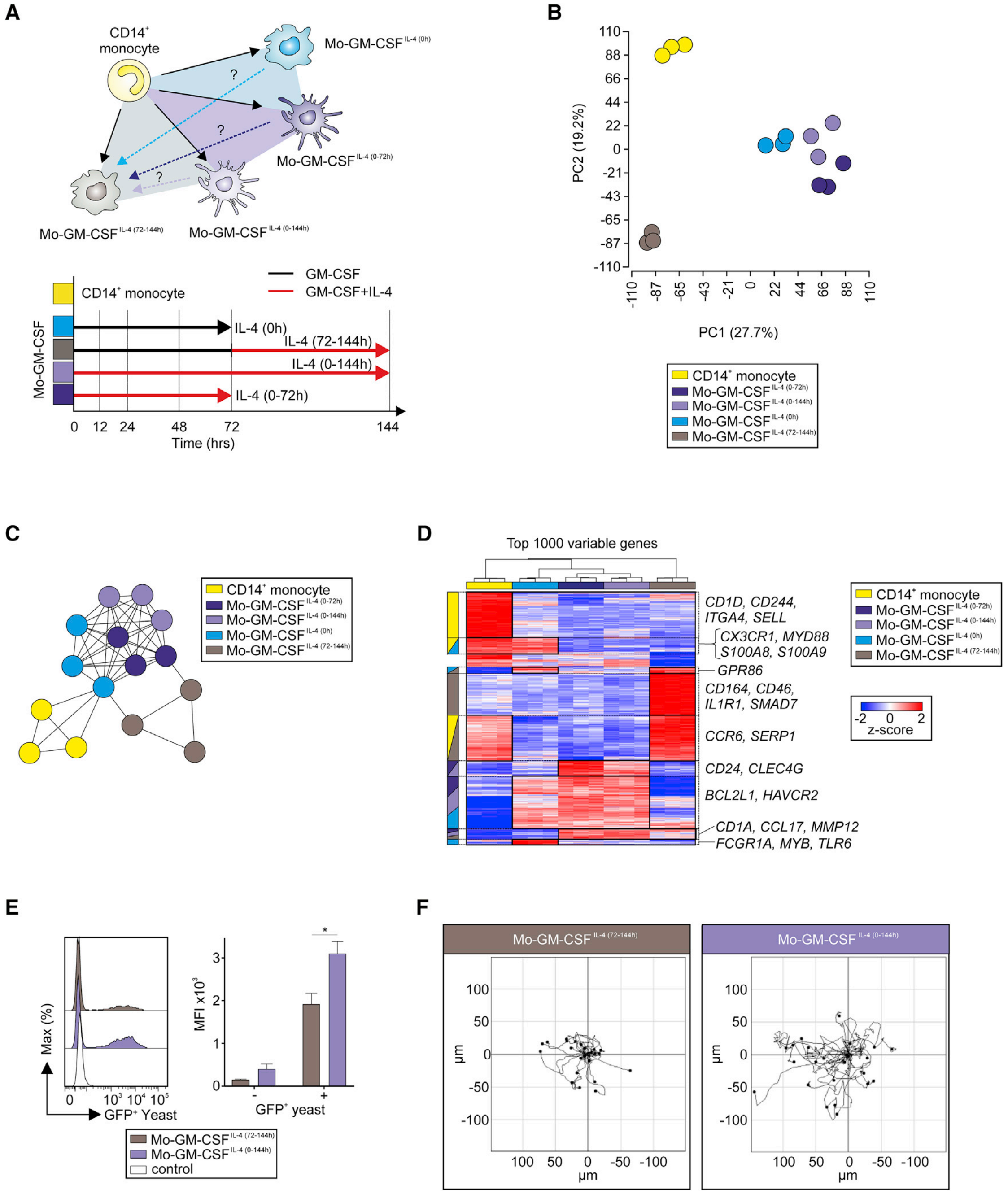

Figure 4. MOs-GM-CSF$^{IL-4(0-144h)}$ Differ from MOs-GM-CSF$^{IL-4(72-144h)}$ MO-Derived Cells

(A) Schema describing questions addressed herein and in Figure S4.

(B) PCA (18,857 present probes).

(C) Co-expression network (13,691 present genes) describing relationships between CD14$^+$ MOs and four types of MO-derived cells.

(D) Heatmap of 1,000 most variable genes in dataset. Log_2-expression values, z-transformed, scaled (−2 [blue] to 2 [red]). Highly expressed genes grouped together (black boxes) according to cell type. Corresponding group-related cell types highlighted, left side of heatmap. Important genes of each cluster depicted, right side of heatmap.

(E) Flow cytometry analysis of MOs-GM-CSF$^{IL-4(72-144h)}$ and MOs-GM-CSF$^{IL-4(0-144h)}$ after incubation with GFP-expressing yeast (1 hr, n = 4–6, mean + SEM, Student's t test with *$p < 0.05$).

(F) Migration tracks (3 hr) of MOs-GM-CSF$^{IL-4(72-144h)}$ and MOs-GM-CSF$^{IL-4(0-144h)}$ (representative result, n = 3).

Please also see Figure S4.

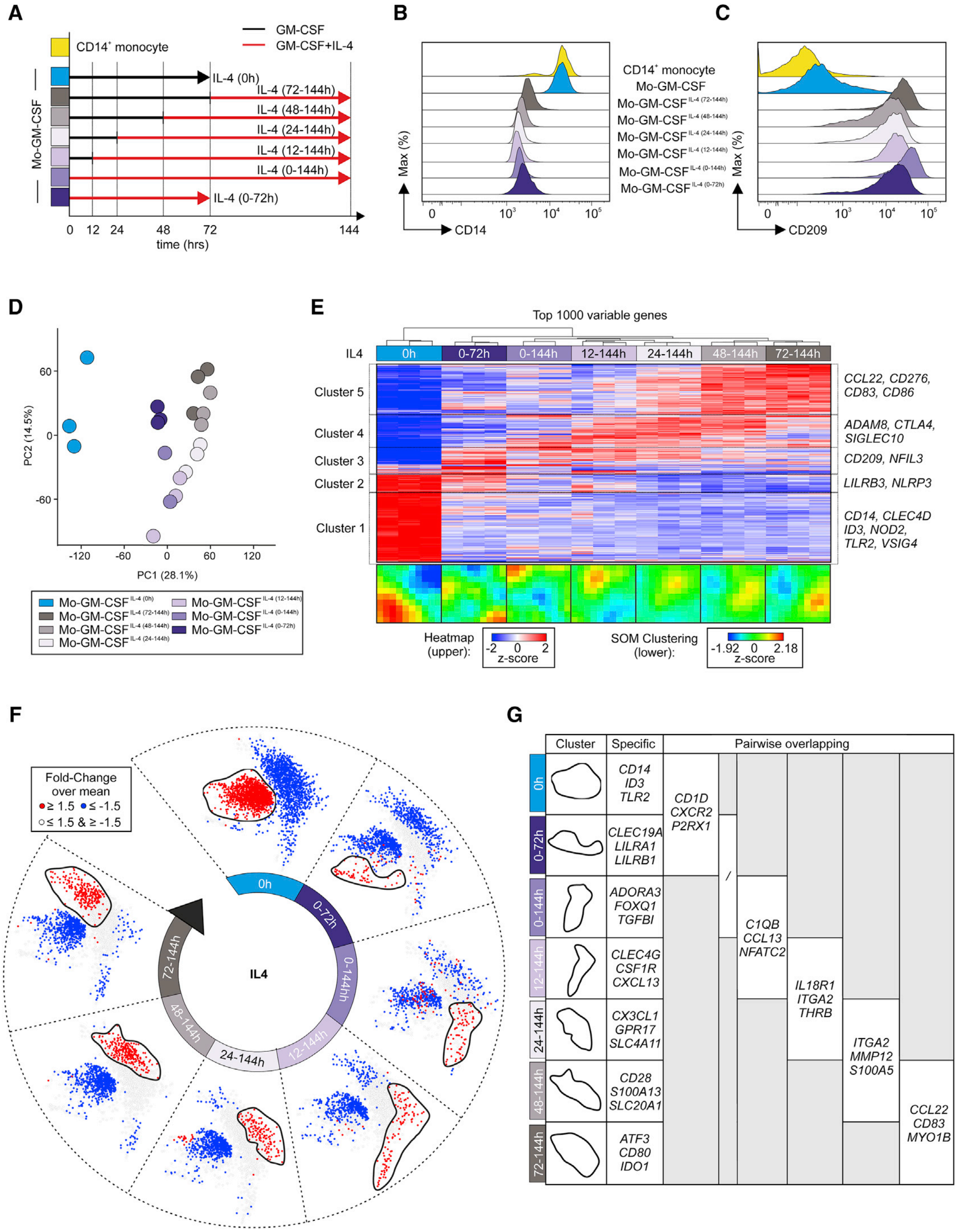

Figure 5. Timing of IL-4 Determines Transcriptional Regulation in Differentiated MOs

(A) Schema describing the IL-4 time kinetic experiment.

(B and C) Histograms, relative expression of CD14 and CD209 analyzed by flow cytometry.

(D) PCA (12,794 present genes).

(E) Heatmap of 1,000 most variable genes across dataset. Log_2-expression values, z-transformed, scaled (−2 [blue] to 2 [red]). Below, SOM-clustering (12,794 present genes across cell types).

(F) Co-expression networks (union of 2,775 DEGs, fold-change > 1.5 or < −1.5 and FDR-corrected p value < 0.05) between MOs-GM-CSF^{IL-4} and MOs-GM-CSF. For each cell type, fold-change of respective cell type versus overall mean mapped onto networks, displayed in blue (fold-change ≤ 1.5) or red (fold-change ≥ 1.5).

(G) Example of genes located in condition-related clusters depicted in (F) and in first column (fold-change ≥ 1.5). First column: condition-specific genes; following columns: genes shared between clusters of two consecutive time points.

Please also see Figure S5.

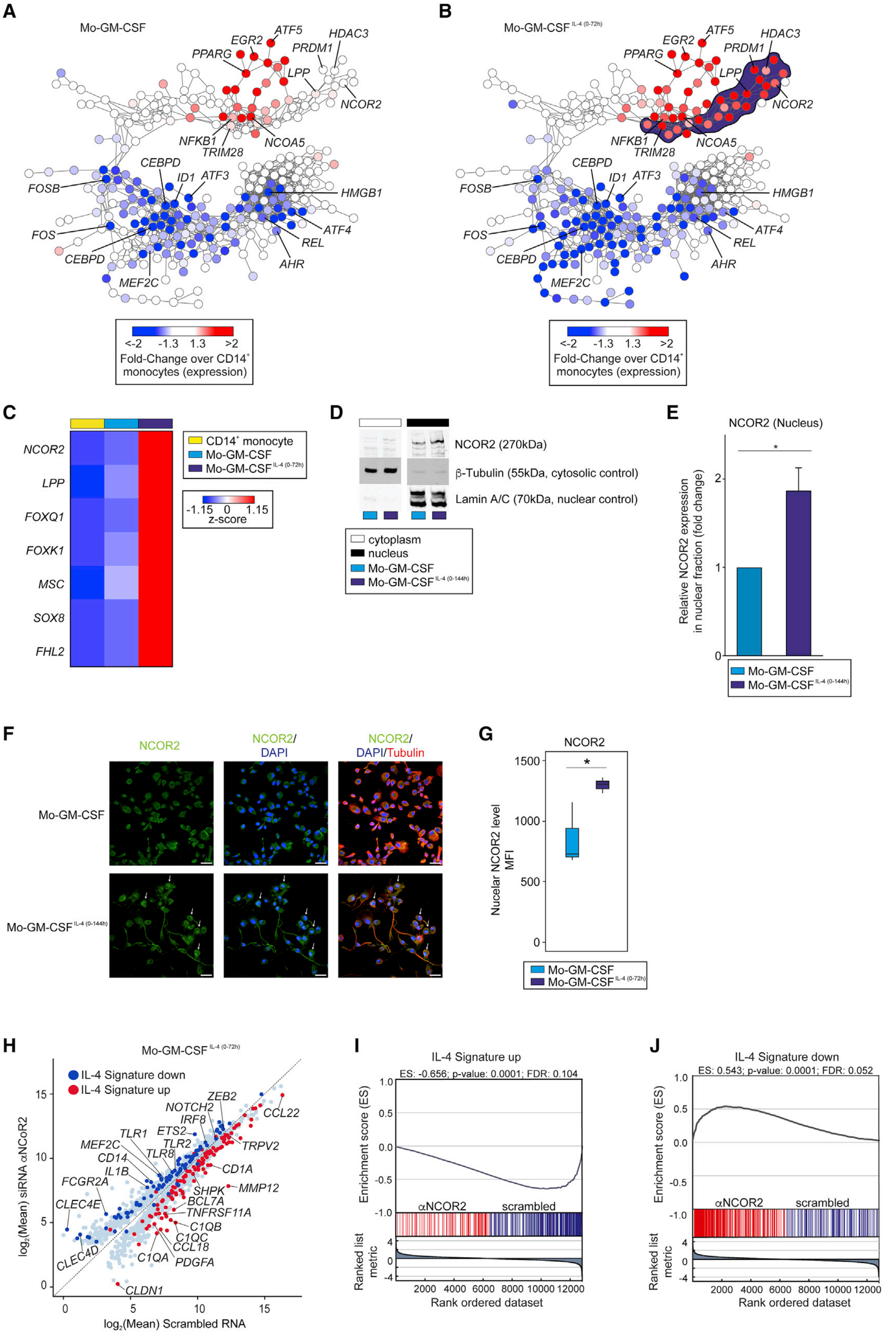

(legend on next page)

transcriptomic continuum with MOs-GM-CSF$^{IL\text{-}4(0h)}$ and MOs-GM-CSF$^{IL\text{-}4(72\text{-}144h)}$ being at the extreme ends.

NCOR2 Is a Transcriptional Regulator of MOs-GM-CSF$^{IL\text{-}4}$

To understand how IL-4 enforces the unique transcriptional program in MOs-GM-CSF$^{IL\text{-}4(0\text{-}72h)}$, a co-expression TR network was generated first using TRs differentially expressed between MOs, MOs-GM-CSF, and MOs-GM-CSF differentiated with different durations of IL-4 (Figures 6A, 6B, and S6A). TRs were then filtered according to differential expression in MOs-GM-CSF$^{IL\text{-}4(0\text{-}72h)}$ and ranked based on absolute expression (Figures 6A and 6B, red: upregulated, blue: downregulated; Table S6). We identified seven eligible TRs with *NCOR2* showing the highest expression (Figure 6C). We confirmed *NCOR2* expression in MOs-GM-CSF and MOs-GM-CSF$^{IL\text{-}4(0\text{-}72h)}$ ($n = 2$) using real-time PCR showing that MOs-GM-CSF$^{IL\text{-}4(0\text{-}72h)}$ expressed significantly more *NCOR2* and *CD209* mRNA (Figures S6B and S6C). Analysis of NCOR2 protein expression by immunoblot analysis (Figures 6D and 6E) or confocal microscopy (Figures 6F and 6G) revealed a significant enrichment of NCOR2 in the nucleus of MOs-GM-CSF$^{IL\text{-}4(0\text{-}144h)}$ but not in MOs-GM-CSF. Using nanostraw technology (Figure S6D), we introduced anti-*NCOR2* siRNAs for the last 24 hr of the MOs-GM-CSF$^{IL\text{-}4(0\text{-}72h)}$ differentiation. After silencing, mean *NCOR2* mRNA expression in MOs-GM-CSF$^{IL\text{-}4(0\text{-}72h)}$ was reduced to 65% relative to the control (Figures S6E and S6F), reflecting effective downregulation of *NCOR2* transcription considering its long half-life of more than 24 hr (Raghavan et al., 2002). siRNA silencing of *NCOR2* in MOs-GM-CSF$^{IL\text{-}4(0\text{-}72h)}$ also reduced *CD209* mRNA (Figures S6E and S6F) and protein (Figure S6G). To understand the impact of NCOR2 on IL-4-mediated transcriptional regulation in MOs-GM-CSF$^{IL\text{-}4(0\text{-}72h)}$, we performed a global transcriptome analysis of anti-*NCOR2* siRNA-treated MOs-GM-CSF$^{IL\text{-}4(0\text{-}72h)}$ versus scrambled siRNA-treated MOs-GM-CSF$^{IL\text{-}4(0\text{-}72h)}$ (Figures S6H and S6I). NCOR2 silencing resulted in 1,834 variable genes (Figure S6J; Table S6). Classification of NCOR2-regulated genes, based on a literature-derived IL-4 signature (GEO: GSE13762, GSE35304, GSE32164; 457 induced, 498 repressed genes) revealed that the large majority of genes regulated by *NCOR2* are IL-4 signature genes (Figures 6H and S6J; Table S6). Additionally, GSEA was performed separately for up- and downregulated genes within the IL-4 signature to identify signature enrichments in the transcriptomes of MOs-GM-CSF$^{IL\text{-}4(0\text{-}72h)}$ treated with scrambled siRNAs or with anti-*NCOR2* siRNAs (Figures 6I and 6J). We found a statistically significant enrichment of genes upregulated in the IL-4 signature in MOs-GM-CSF$^{IL\text{-}4(0\text{-}72h)}$ treated with scrambled siRNAs (Figure 6I) whereas genes downregulated in the IL-4 signature were enriched in MOs-GM-CSF$^{IL\text{-}4(0\text{-}72h)}$ treated with anti-*NCOR2* siRNAs (Figure 6J), establishing *NCOR2* as a key regulator for IL-4-induced MO differentiation.

MC Identifies Heterogeneity in Surface Molecules Expressed by MOs-GM-CSF$^{IL\text{-}4(0\text{-}144h)}$

Within the MC-defined populations (MOs-M-CSF, MOs-GM-CSF, and MOs-GM-CSF$^{IL\text{-}4(0\text{-}144h)}$) (Figure 2G), we recognized a sub-cluster structure indicating further heterogeneity within each *in vitro* subset (Figures 7A and 7B). MOs-M-CSF revealed four subpopulations (clusters 2, 5, 10, 11) characterized by co-expression of tissue Mac markers MERTK, CD64, CD169, and CD163 and by variable expression of L-selectin (CD62L, low in cluster 2, 11) and CD26 (low in cluster 2), indicating different migration and maturation profiles (Figure 7B). MOs-GM-CSF were defined by two subclusters (clusters 7 and 8). Both clusters expressed CD14, CD64, CD68, and CD206, but not Mac markers such as MERTK, CD169, and CD163, further corroborating their difference from MOs-M-CSF. Cluster 7 additionally expressed the molecules FcεR1 and CD1a, supporting an activated status of this subpopulation. MOs-GM-CSF$^{IL\text{-}4(0\text{-}144h)}$ showed a similar heterogeneity as MOs-M-CSF, but with more pronounced phenotypic differences (cluster 1, 3, 4, 9). Therefore, we isolated the MOs-GM-CSF$^{IL\text{-}4(0\text{-}144h)}$ dataset and analyzed it using Rphenograph (Figures 7C–7E). This analysis revealed 11 phenotypically different clusters within MOs-GM-CSF$^{IL\text{-}4(0\text{-}144h)}$ (Figures 7C and 7D). All 11 subpopulations expressed the moDC markers CD1c, CD226, CD48, and CD11c. The biggest differences across all different subpopulations were seen in the expression of activation and antigen presentation-associated molecules, such as CD1a, HLA-DR, FcεR1, CD62L, and CD86. Marker expression differed between the clusters 6, 1, 10, 11 showing high expression of CD1a and CD62L, whereas only cluster 5 and 2 expressed high amounts of the co-stimulatory molecule CD86. Expression of CD11b, a marker used to analyze the purity of moDCs in the clinic, varied across the examined moDC population, exemplifying the urgent need for

Figure 6. NCOR2 Is a Transcriptional Regulator of MOs-GM-CSF$^{IL\text{-}4(0\text{-}72/144h)}$

(A and B) Co-expression networks (267 TRs) for (A) MOs-GM-CSF, (B) MOs-GM-CSF$^{IL\text{-}4(0\text{-}72h)}$, fold-change versus CD14^{+} MOs mapped onto network. MOs-GM-CSF$^{IL\text{-}4(0\text{-}72h)}$-specific cluster of elevated regulators (dark blue).

(C) TR Heatmap, specifically upregulated in MOs-GM-CSF$^{IL\text{-}4(0\text{-}72h)}$, MOs-GM-CSF$^{IL\text{-}4(0\text{-}144h)}$ versus CD14^{+} MOs, MOs-GM-CSF. Log_2-expression values, z-transformed, scaled (-1.15 [blue] to 1.15 [red]).

(D) Immunoblot of NCOR2, Lamin A/C, and β-tubulin in cytoplasm and nucleus of MOs-GM-CSF and MOs-GM-CSF$^{IL\text{-}4(0\text{-}144h)}$ (representative result, $n = 4$).

(E) Quantification of relative enrichment of NCOR2 in nuclear fractions isolated from MOs-GM-CSF and MOs-GM-CSF$^{IL\text{-}4(0\text{-}144h)}$ ($n = 4$, $^{*}p < 0.05$, mean ± SEM).

(F) Representative confocal microscopy images of medial nuclear region (MNR) of MOs-GM-CSF and MOs-GM-CSF$^{IL\text{-}4(0\text{-}144h)}$ ($n = 3$, green: NCOR2; blue: DAPI; red: tubulin).

(G) Quantification of mean fluorescence intensity of NCOR2 in MNR identified by confocal microscopy of GM-CSF and MOs-GM-CSF$^{IL\text{-}4(0\text{-}144h)}$ ($n = 3$, green: NCOR2; blue: DAPI; red: tubulin, $^{*}p < 0.05$, mean ± SEM).

(H) Scatterplot (1,834 variable genes in dataset) containing cells treated with α*NCOR2* (y axis), scrambled siRNA (x axis, log_2-mean expression values). Highlighted genes determined (see Figure S6F) induced (red) or repressed (blue) by IL-4.

(I and J) GSEA of genes upregulated (I), downregulated (J) in IL-4 signature in MOs-GM-CSF$^{IL\text{-}4(0\text{-}72h)}$ treated with scrambled or α*NCOR2* siRNA (nominal p value, empirical phenotype-based permutation test [$p < 0.05$, FDR < 0.25], 1,000 samples permutations).

Please also see Figure S6.

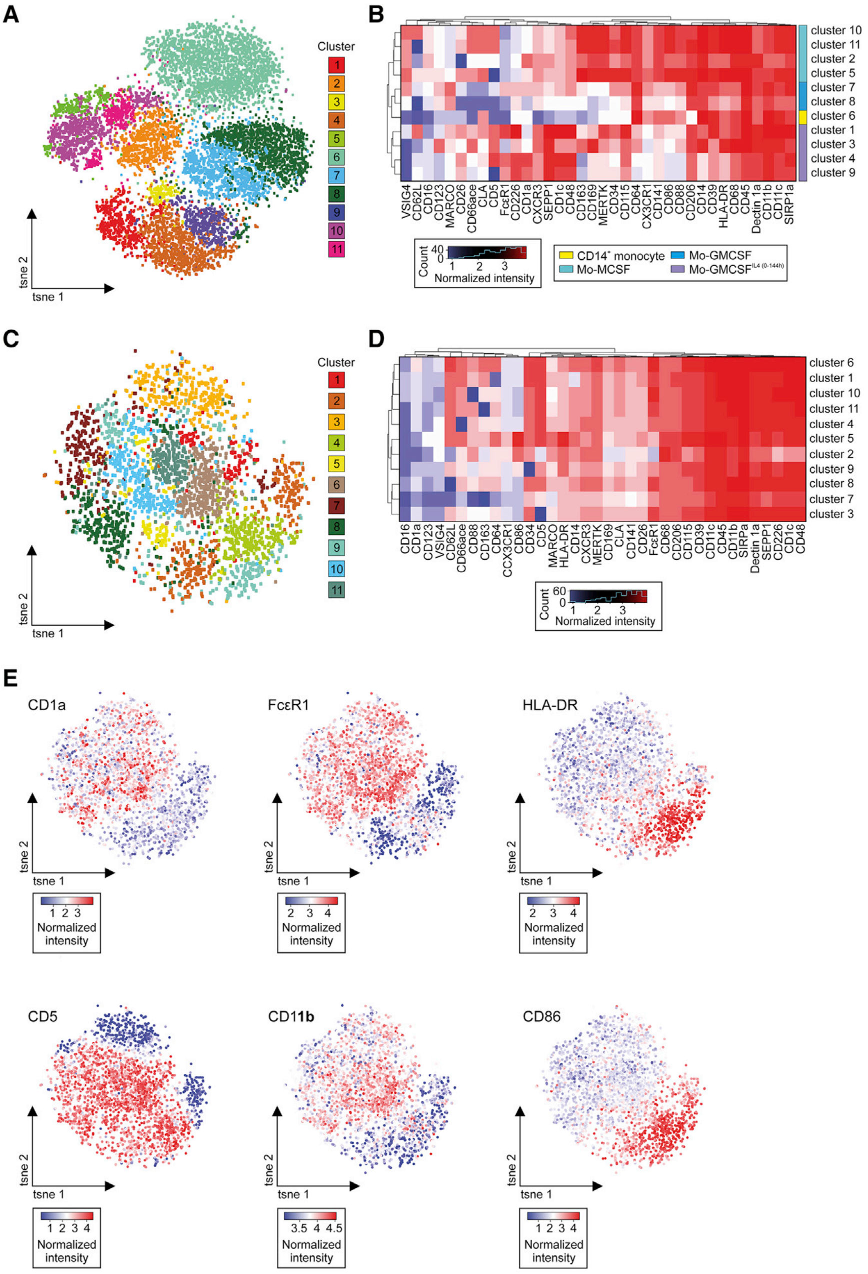

Figure 7. Mass Cytometry Analysis Identifies Unappreciated Phenotypic Heterogeneity in Clinically Relevant Mo-GM-CSF$^{IL-4(0-144h)}$ Cultures

(A) Phenograph of CD14^{+} MOs, MOs-M-CSF, MOs-GM-CSF, and MOs-GM-CSF$^{IL-4(0-144h)}$ based on MC expression data (n = 3, 36 myeloid-related surface markers). Affiliation of cells to the 11 identified clusters indicated by color coding and visualized in *t*-SNE plot.

(B) Heatmap and HC of mean surface marker expression of 11 individual clusters. Right side: Differentiating conditions according to (A) and Figure 2G.

(C) Phenograph of MOs-GM-CSF$^{IL4(0-144h)}$ and visualized in *t*-SNE plot (representative donor; 3,500 cells).

(D) Heatmap and HC of mean surface marker expression of 11 individual clusters.

(E) Expression feature plot of the depicted surface markers in MOs-GM-CSF$^{IL-4(0-144h)}$.

a higher-dimensional phenotyping to improve purity and therapeutic outcome when using these differentiated MOs therapeutically (Figure 7E).

DISCUSSION

Human CD14^{+} MOs differentiated by either M-CSF, GM-CSF, or GM-CSF + IL-4 (formerly moDCs [Sallusto and Lanzavecchia, 1994]) have been extensively studied as *in vitro* models for Macs, "M2-like" Macs, or DCs (Akagawa et al., 2006; Sallusto and Lanzavecchia, 1994). In the past, *in vitro* generated Mac and DC models were justified by morphological, phenotypical, and functional similarities to cells identified *in vivo*. Recently, it became clear that Macs, MOs, and DCs present with very different transcriptomes *in vivo*, reflecting different ontogeny (Guilliams et al., 2014). Therefore, reassessment of the relationships between M-CSF-, GM-CSF-, and GM-CSF + IL-4-differentiated MOs and their alleged *in vivo* counterparts on a transcriptomic, phenotypic, and functional level is imperative.

Using data of ascites-associated infMs and infDCs (Segura et al., 2013) revealed that MOs-M-CSF aligned closely to infMs on the transcriptional level. This was phenotypically supported by low expression of CD1a and FcεR1 while MOs-GM-CSF^{IL-4} aligned closely to infDCs with high expression of CD1a, CD206, and FcεR1. Taken together, the currently available *in vitro* models best resemble inflammatory rather than homeostatic DC and Mac populations, therefore, serving best as reductionist models to study the role of these cells in inflammation. For the future, we encourage researchers to identify culture conditions for MO-derived cells that resemble homeostatic DC or Mac phenotypes guided by transcriptomic analysis, as shown for *in vitro* cultures of microglia (Butovsky et al., 2014), and pursue the identification of dedicated progenitors of DCs in the human blood and bone marrow (Breton et al., 2015).

Transcriptome analysis defined the cellular relationships between these model systems on a much more precise level and revealed a close association of MOs differentiated by M-CSF and GM-CSF, while IL-4 was the major driver for moDC identity. In addition, we found that MOs integrate the GM-CSF and IL-4 signals over time, which necessitates a reassessment of our dichotomous definition of MOs differentiating toward a Mac or DC-like phenotype. The varying time of onset and the variance in overall exposure to IL-4 resulted in gradually

changed transcriptional and functional identities of MO-derived cells. The heterogeneity defined by MCs added an additional layer of individual cell reactivity. These observations challenge a dichotomous differentiation model of MOs toward Macs or DCs induced by a single cytokine, and instead support a dynamic model in which cell identity is a function of the duration of signal input. These findings might also help to better understand IL-4-mediated MO-derived cell expansion during Th2 cell-driven inflammatory conditions, such as parasitic infections and tissue repair (Rückerl and Allen, 2014).

We identified NCOR2 as a key transcriptional hub linked to IL-4-dependent differentiation of MOs. NCOR2 plays an important role during development and homeostatic processes in muscle, adipose, and liver tissues (Mottis et al., 2013). NCOR2 has been shown to be important for cell fate, differentiation, and lineage progression in the nervous system (Jepsen et al., 2007). Elevated NCOR2 expression was observed in tissues with high OXPHOS activity, similar to our observations of elevated expression of NCOR2 in MOs-GM-CSF^{IL-4} (Reilly et al., 2010). Additionally, signaling by nuclear receptors, such as peroxisome proliferator-activated receptor-γ (PPAR-γ) or liver X receptor, NCOR2 was linked to the repression of NF-κB target genes in response to LPS stimulation of Macs (Pascual et al., 2005). It has been speculated that IL-4 activation of human MOs leads to endogenous PPAR-γ ligand production (Czimmerer et al., 2012), but further work is necessary to establish a link to NCOR2-mediated gene repression.

Unexpectedly, we identified significant heterogeneity within MO cultures, which was revealed only by a set of surface markers as applied by MCs. In particular, within MOs-GM-CSF^{IL-4} we identified subsets that either expressed HLA-DR and CD86 or CD1a and FcεR1, the former representing a subpopulation with elevated antigen presenting capacity. While induction of FcεR1 on CD1a$^+$ DCs derived from CD34$^+$ stem cells has been reported (Allam et al., 2004), this has not been studied during MO to MO-GM-CSF^{IL-4} differentiation. Considering findings that CD1a$^+$ and CD1a$^-$ MOs-GM-CSF^{IL-4} differ in their capacity to direct Th cell differentiation (Cernadas et al., 2009), monitoring of these cultures in a clinical setting might be beneficial for optimizing efficiency of cellular vaccines. Furthermore, in studies using bulk MOs instead of CD14$^+$ MOs, the problem of heterogeneity might even be more pronounced and should be addressed when analyzing *in vitro* MO cultures. Consequently, high-dimensional characterization should be used to optimize culture conditions, generate more homogeneous cell populations, and thereby open avenues for optimizing cellular products derived from human MOs for vaccination strategies.

STAR★METHODS

Detailed methods are provided in the online version of this paper and include the following:

- KEY RESOURCES TABLE
- CONTACT FOR REAGENT AND RESOURCE SHARING
- EXPERIMENTAL MODEL AND SUBJECT DETAILS
  - Human primary cell isolation
  - Human primary cell culture
- METHOD DETAILS
  - Flow cytometry
  - Mass cytometry
  - Mass cytometry data analysis
  - Uptake of fluorescent microbeads or yeast
  - Immuno blot
  - Migration assay
  - Confocal microscopy and data analysis
  - NCOR2 siRNA silencing
  - *Real time* PCR
  - Cell stimulation
  - Cytokine measurement
  - Oxygen consumption rate (OCR) and ECAR measurements
  - Microarray data generation
  - RNA-sequencing
  - Primary handling of microarray datasets
  - Handling of publicly available data
  - Probe matching between platforms
  - Assembly of datasets and pre-processing
  - Primary data analysis and visualization
  - Identification of signature genes
  - Linear support vector regression (SVR)
  - GSEA analysis
  - Correlation of gene expression
  - Identification of subset specific genes
  - Co-expression network analysis
  - Prediction of master regulators
  - Transcriptional regulator Co-expression network
  - Expression profiles of pattern recognition receptors
  - Comparison of Mo-M-CSF and Mo-GM-CSF cells
  - Expression of genes linked to YG bead uptake, motility or OCR/ECAR
  - Visualization of cytokine responses
  - Handling of the RNA-Seq time kinetics dataset
  - Analysis and visualization of RNA-Seq time kinetics dataset
  - Co-expression network for the time kinetics dataset
  - IL-4 signature generation
  - Handling of the siRNA αNCoR2 dataset
  - Analysis and visualization of the siRNA αNCoR2 dataset
  - GSEA
- QUANTIFICATION AND STATISTICAL ANALYSIS
- DATA AND SOFTWARE AVAILABILTIY

SUPPLEMENTAL INFORMATION

Supplemental Information includes six figures, seven tables, and one movie and can be found with this article online at https://doi.org/10.1016/j.immuni.2017.11.024.

ACKNOWLEDGMENTS

We thank J. Oldenburg for buffy coats from healthy individuals and M. Ballmaier for cell sorting at MHH. This work was supported by the German Research Foundation (SFB 832, SFB 704, INST 217/577-1, and SFB 738 to C.S.F.), core grants of the Singapore Immunology Network (F.G., E.W.N.), and the Singapore Immunology Network immune-monitoring platform funding (E.W.N.). J.L.S., A.S., W. Kolanus, E.L., S.B., and M.B. are members of the Excellence Cluster ImmunoSensation. The research leading to these results

received funding from the People Program (Marie Curie Actions) of the European Union's Seventh Framework Program FP7/2077-2013 under REA grant agreement no. 317445. A.S. is supported by an Emmy Noether fellowship of the German Research foundation (SCHL2116/1-1). N.McG. is supported by a Royal Society and Wellcome Trust grant (204464/Z/16/Z).

AUTHOR CONTRIBUTIONS

Conceptualization, J.S., S.V.S., A.S., J.L.S.; Methodology, J.S., A.S., J.L.S., F.G., E.L., E.W.N., N.A.M., S.B., W. Kolanus, M.B., C.S.F.; Investigation, J.S., S.V.S., B.C., N.McG., O.P., A.-L.H., A.C.A., C.K., T.Q., A.M.X., L.M.S., H.T., T.H.L.D., H.R.B.S., M.A.R.L., J.S., P.G., J.X., K.B., T.U., K.K., N.K., S.H., W. Krebs, B.M., K.H., M.K., B.W., J.S.-S.; Writing, J.S., A.S., J.L.S.; Funding Acquisition, J.L.S.

Received: March 31, 2017
Revised: September 15, 2017
Accepted: November 28, 2017
Published: December 19, 2017

REFERENCES

Akagawa, K.S., Komuro, I., Kanazawa, H., Yamazaki, T., Mochida, K., and Kishi, F. (2006). Functional heterogeneity of colony-stimulating factor-induced human monocyte-derived macrophages. Respirology *11* (*Suppl*), S32–S36.

Allam, J.-P., Klein, E., Bieber, T., and Novak, N. (2004). Transforming growth factor-beta1 regulates the expression of the high-affinity receptor for IgE on CD34 stem cell-derived CD1a dendritic cells in vitro. J. Invest. Dermatol. *123*, 676–682.

Auffray, C., Sieweke, M.H., and Geissmann, F. (2009). Blood monocytes: development, heterogeneity, and relationship with dendritic cells. Annu. Rev. Immunol. *27*, 669–692.

Bonilla, D.L., Bhattacharya, A., Sha, Y., Xu, Y., Xiang, Q., Kan, A., Jagannath, C., Komatsu, M., and Eissa, N.T. (2013). Autophagy regulates phagocytosis by modulating the expression of scavenger receptors. Immunity *39*, 537–547.

Breton, G., Lee, J., Zhou, Y.J., Schreiber, J.J., Keler, T., Puhr, S., Anandasabapathy, N., Schlesinger, S., Caskey, M., Liu, K., and Nussenzweig, M.C. (2015). Circulating precursors of human CD1c+ and CD141+ dendritic cells. J. Exp. Med. *207*, 1273–1413.

Brower, V. (2005). Cancer vaccine field gets shot of optimism from positive results. Nat. Med. *11*, 360–360.

Butovsky, O., Jedrychowski, M.P., Moore, C.S., Cialic, R., Lanser, A.J., Gabriely, G., Koeglsperger, T., Dake, B., Wu, P.M., Doykan, C.E., et al. (2014). Identification of a unique TGF-β-dependent molecular and functional signature in microglia. Nat. Neurosci. *17*, 131–143.

Cardoso, C.M.P., Jordao, L., and Vieira, O.V. (2010). Rab10 regulates phagosome maturation and its overexpression rescues *Mycobacterium*-containing phagosomes maturation. Traffic *11*, 221–235.

Cella, M., Döhring, C., Samaridis, J., Dessing, M., Brockhaus, M., Lanzavecchia, A., and Colonna, M. (1997). A novel inhibitory receptor (ILT3) expressed on monocytes, macrophages, and dendritic cells involved in antigen processing. J. Exp. Med. *185*, 1743–1751.

Cernadas, M., Lu, J., Watts, G., and Brenner, M.B. (2009). CD1a expression defines an interleukin-12 producing population of human dendritic cells. Clin. Exp. Immunol. *155*, 523–533.

Curtis, J., Luo, Y., Zenner, H.L., Cuchet-Lourenço, D., Wu, C., Lo, K., Maes, M., Alisaac, A., Stebbings, E., Liu, J.Z., et al. (2015). Susceptibility to tuberculosis is associated with variants in the ASAP1 gene encoding a regulator of dendritic cell migration. Nat. Genet. *47*, 523–527.

Czimmerer, Z., Varga, T., Póliska, S., Nemet, I., Szanto, A., and Nagy, L. (2012). Identification of novel markers of alternative activation and potential endogenous PPARγ ligand production mechanisms in human IL-4 stimulated differentiating macrophages. Immunobiology *217*, 1301–1314.

de Souza, J.E.S., Galante, P.A.F., de Almeida, R.V.B., da Cunha, J.P.C., Ohara, D.T., Ohno-Machado, L., Old, L.J., and de Souza, S.J. (2012). SurfaceomeDB: a cancer-orientated database for genes encoding cell surface proteins. Cancer Immun. *12*, 15.

Fehlings, M., Drobbe, L., Moos, V., Renner Viveros, P., Hagen, J., Beigier-Bompadre, M., Pang, E., Belogolova, E., Churin, Y., Schneider, T., et al. (2012). Comparative analysis of the interaction of Helicobacter pylori with human dendritic cells, macrophages, and monocytes. Infect. Immun. *80*, 2724–2734.

Fraser, D.A., Laust, A.K., Nelson, E.L., and Tenner, A.J. (2009). C1q differentially modulates phagocytosis and cytokine responses during ingestion of apoptotic cells by human monocytes, macrophages, and dendritic cells. J. Immunol. *183*, 6175–6185.

Frucht, D.M., Aringer, M., Galon, J., Danning, C., Brown, M., Fan, S., Centola, M., Wu, C.Y., Yamada, N., El Gabalawy, H., and O'Shea, J.J. (2000). Stat4 is expressed in activated peripheral blood monocytes, dendritic cells, and macrophages at sites of Th1-mediated inflammation. J. Immunol. *164*, 4659–4664.

Fulton, D.L., Sundararajan, S., Badis, G., Hughes, T.R., Wasserman, W.W., Roach, J.C., and Sladek, R. (2009). TFCat: the curated catalog of mouse and human transcription factors. Genome Biol. *10*, R29.

Garg, A.D., Coulie, P.G., Van den Eynde, B.J., and Agostinis, P. (2017). Integrating next-generation dendritic cell vaccines into the current cancer immunotherapy landscape. Trends Immunol. *38*, 577–593.

Geijtenbeek, T.B., Torensma, R., van Vliet, S.J., van Duijnhoven, G.C., Adema, G.J., van Kooyk, Y., and Figdor, C.G. (2000). Identification of DC-SIGN, a novel dendritic cell-specific ICAM-3 receptor that supports primary immune responses. Cell *100*, 575–585.

Ginhoux, F., and Guilliams, M. (2016). Tissue-resident macrophage ontogeny and homeostasis. Immunity *44*, 439–449.

Godiska, R., Chantry, D., Raport, C.J., Sozzani, S., Allavena, P., Leviten, D., Mantovani, A., and Gray, P.W. (1997). Human macrophage-derived chemokine (MDC), a novel chemoattractant for monocytes, monocyte-derived dendritic cells, and natural killer cells. J. Exp. Med. *185*, 1595–1604.

Grajales-Reyes, G.E., Iwata, A., Albring, J., Wu, X., Tussiwand, R., Kc, W., Kretzer, N.M., Briseño, C.G., Durai, V., Bagadia, P., et al. (2015). Batf3 maintains autoactivation of Irf8 for commitment of a CD8α(+) conventional DC clonogenic progenitor. Nat. Immunol. *16*, 708–717.

Guilliams, M., and van de Laar, L. (2015). A hitchhiker's guide to myeloid cell subsets: practical implementation of a novel mononuclear phagocyte classification system. Front. Immunol. *6*, 406.

Guilliams, M., Ginhoux, F., Jakubzick, C., Naik, S.H., Onai, N., Schraml, B.U., Segura, E., Tussiwand, R., and Yona, S. (2014). Dendritic cells, monocytes and macrophages: a unified nomenclature based on ontogeny. Nat. Rev. Immunol. *14*, 571–578.

Haniffa, M., Shin, A., Bigley, V., McGovern, N., Teo, P., See, P., Wasan, P.S., Wang, X.N., Malinarich, F., Malleret, B., et al. (2012). Human tissues contain CD141hi cross-presenting dendritic cells with functional homology to mouse CD103+ nonlymphoid dendritic cells. Immunity *37*, 60–73.

Helft, J., Böttcher, J., Chakravarty, P., Zelenay, S., Huotari, J., Schraml, B.U., Goubau, D., and Reis e Sousa, C. (2015). GM-CSF mouse bone marrow cultures comprise a heterogeneous population of CD11c(+)MHCII(+) macrophages and dendritic cells. Immunity *42*, 1197–1211.

Jepsen, K., Solum, D., Zhou, T., McEvilly, R.J., Kim, H.-J., Glass, C.K., Hermanson, O., and Rosenfeld, M.G. (2007). SMRT-mediated repression of an H3K27 demethylase in progression from neural stem cell to neuron. Nature *450*, 415–419.

Lacey, D.C., Achuthan, A., Fleetwood, A.J., Dinh, H., Roiniotis, J., Scholz, G.M., Chang, M.W., Beckman, S.K., Cook, A.D., and Hamilton, J.A. (2012). Defining GM-CSF- and macrophage-CSF-dependent macrophage responses by in vitro models. J. Immunol. *188*, 5752–5765.

Maaten, L.V.D., and Hinton, G. (2008). Visualizing Data using t-SNE. J. Mach. Learn. Res. *9*, 2579–2605.

Mantovani, A., Sozzani, S., Locati, M., Allavena, P., and Sica, A. (2002). Macrophage polarization: tumor-associated macrophages as a paradigm for polarized M2 mononuclear phagocytes. Trends Immunol. *23*, 549–555.

Merad, M., Sathe, P., Helft, J., Miller, J., and Mortha, A. (2013). The dendritic cell lineage: ontogeny and function of dendritic cells and their subsets in the steady state and the inflamed setting. Annu. Rev. Immunol. *31*, 563–604.

Mottis, A., Mouchiroud, L., and Auwerx, J. (2013). Emerging roles of the corepressors NCoR1 and SMRT in homeostasis. Genes Dev. *27*, 819–835.

Newman, A.M., Liu, C.L., Green, M.R., Gentles, A.J., Feng, W., Xu, Y., Hoang, C.D., Diehn, M., and Alizadeh, A.A. (2015). Robust enumeration of cell subsets from tissue expression profiles. Nat. Methods *12*, 453–457.

Ohradanova-Repic, A., Machacek, C., Fischer, M.B., and Stockinger, H. (2016). Differentiation of human monocytes and derived subsets of macrophages and dendritic cells by the HLDA10 monoclonal antibody panel. Clin. Transl. Immunology *5*, e55.

Pascual, G., Fong, A.L., Ogawa, S., Gamliel, A., Li, A.C., Perissi, V., Rose, D.W., Willson, T.M., Rosenfeld, M.G., and Glass, C.K. (2005). A SUMOylation-dependent pathway mediates transrepression of inflammatory response genes by PPAR-gamma. Nature *437*, 759–763.

Raghavan, A., Ogilvie, R.L., Reilly, C., Abelson, M.L., Raghavan, S., Vasdewani, J., Krathwohl, M., and Bohjanen, P.R. (2002). Genome-wide analysis of mRNA decay in resting and activated primary human T lymphocytes. Nucleic Acids Res. *30*, 5529–5538.

Reilly, S.M., Bhargava, P., Liu, S., Gangl, M.R., Gorgun, C., Nofsinger, R.R., Evans, R.M., Qi, L., Hu, F.B., and Lee, C.-H. (2010). Nuclear receptor corepressor SMRT regulates mitochondrial oxidative metabolism and mediates aging-related metabolic deterioration. Cell Metab. *12*, 643–653.

Rossi, R., Lichtner, M., De Rosa, A., Sauzullo, I., Mengoni, F., Massetti, A.P., Mastroianni, C.M., and Vullo, V. (2011). In vitro effect of anti-human immunodeficiency virus CCR5 antagonist maraviroc on chemotactic activity of monocytes, macrophages and dendritic cells. Clin. Exp. Immunol. *166*, 184–190.

Rückerl, D., and Allen, J.E. (2014). Macrophage proliferation, provenance, and plasticity in macroparasite infection. Immunol. Rev. *262*, 113–133.

Sallusto, F., and Lanzavecchia, A. (1994). Efficient presentation of soluble antigen by cultured human dendritic cells is maintained by granulocyte/macrophage colony-stimulating factor plus interleukin 4 and downregulated by tumor necrosis factor alpha. J. Exp. Med. *179*, 1109–1118.

Schlitzer, A., McGovern, N., and Ginhoux, F. (2015a). Dendritic cells and monocyte-derived cells: Two complementary and integrated functional systems. Semin. Cell Dev. Biol. *41*, 9–22.

Schlitzer, A., Sivakamasundari, V., Chen, J., Sumatoh, H.R.B., Schreuder, J., Lum, J., Malleret, B., Zhang, S., Larbi, A., Zolezzi, F., et al. (2015b). Identification of cDC1- and cDC2-committed DC progenitors reveals early lineage priming at the common DC progenitor stage in the bone marrow. Nat. Immunol. *16*, 718–728.

See, P., Dutertre, C.-A., Chen, J., Günther, P., McGovern, N., Irac, S.E., Gunawan, M., Beyer, M., Händler, K., Duan, K., et al. (2017). Mapping the human DC lineage through the integration of high-dimensional techniques. Science *356*, 356.

Segura, E., Touzot, M., Bohineust, A., Cappuccio, A., Chiocchia, G., Hosmalin, A., Dalod, M., Soumelis, V., and Amigorena, S. (2013). Human inflammatory dendritic cells induce Th17 cell differentiation. Immunity *38*, 336–348.

Stellato, C., Collins, P., Ponath, P.D., Soler, D., Newman, W., La Rosa, G., Li, H., White, J., Schwiebert, L.M., Bickel, C., et al. (1997). Production of the novel C-C chemokine MCP-4 by airway cells and comparison of its biological activity to other C-C chemokines. J. Clin. Invest. *99*, 926–936.

Toujas, L., Delcros, J.G., Diez, E., Gervois, N., Semana, G., Corradin, G., and Jotereau, F. (1997). Human monocyte-derived macrophages and dendritic cells are comparably effective in vitro in presenting HLA class I-restricted exogenous peptides. Immunology *91*, 635–642.

Tumbarello, D.A., Kendrick-Jones, J., and Buss, F. (2013). Myosin VI and its cargo adaptors - linking endocytosis and autophagy. J. Cell Sci. *126*, 2561–2570.

Varol, C., Mildner, A., and Jung, S. (2015). Macrophages: development and tissue specialization. Annu. Rev. Immunol. *33*, 643–675.

Wu, L., D'Amico, A., Winkel, K.D., Suter, M., Lo, D., and Shortman, K. (1998). RelB is essential for the development of myeloid-related CD8alpha- dendritic cells but not of lymphoid-related CD8alpha+ dendritic cells. Immunity *9*, 839–847.

Xue, J., Schmidt, S.V., Sander, J., Draffehn, A., Krebs, W., Quester, I., De Nardo, D., Gohel, T.D., Emde, M., Schmidleithner, L., et al. (2014). Transcriptome-based network analysis reveals a spectrum model of human macrophage activation. Immunity *40*, 274–288.

Yu, P., Wang, Y., Chin, R.K., Martinez-Pomares, L., Gordon, S., Kosco-Vibois, M.H., Cyster, J., and Fu, Y.X. (2002). B cells control the migration of a subset of dendritic cells into B cell follicles via CXC chemokine ligand 13 in a lymphotoxin-dependent fashion. J. Immunol. *168*, 5117–5123.

STAR★METHODS

KEY RESOURCES TABLE

REAGENT or RESOURCE	SOURCE	IDENTIFIER
Antibodies		
APC/Cy7 anti-human CD19	BioLegend	Cat# 302218, RRID:AB_314248
Alexa Fluor 647 anti-human CD56	BD Biosciences	Cat# 557711, RRID:AB_396820
APC anti-human CD11b	BioLegend	Cat# 301410, RRID:AB_2280647
APC/Cy7 anti-human CD23	BioLegend	Cat# 338520, RRID:AB_10708699
PerCP anti-human HLA-DR	BD Biosciences	Cat# 347402
Pacific Blue anti-human CD14 antibody	BioLegend	Cat# 301828, RRID:AB_2275670
Anti-Human Vsig4	R and D Systems	Cat# AF4646, RRID:AB_2257239
Anti-Human MARCO	R and D Systems	Cat# AF7586
Anti-human MMP-12	R and D Systems	Cat# AF914
FITC anti-human CD209	BD Biosciences	Cat# 551264, RRID:AB_394122
FITC anti-human CD226	BD Biosciences	Cat# 559788, RRID:AB_397329
PE anti-human CCR7	R and D Systems	Cat# FAB197P, RRID:AB_2244252
Anti-human NCOR2	Abcam	Cat# ab24551
Anti-human Lamin A/C	Active Motif	Cat# 39287
Anti-human beta-Tubulin	Cell Signaling	Cat# 15115
Anti-human Tubulin	Chemicon	Cat# MAB1864, RRID:AB_2210391
Anti-Actin	Millipore	Cat# MAB1501, RRID:AB_2223041
Goat anti-Rabbit IgG (H+L), Alexa Fluor 488 conjugate	Thermo Fisher Scientific	Cat# A-11034, RRID:AB_2576217
Goat anti-Rat IgG (H+L), Alexa Fluor 555 conjugate	Thermo Fisher Scientific	Cat# A-21434, RRID:AB_2535855
Qdot 605 anti-human CD14	Invitrogen	Cat# Q10013, RRID:AB_2556439
Y89 anti-human CD45	Fluidigm	Cat# 3089003, RRID:AB_2661851
Anti-human CD15	BioLegend	Cat# 301907, RRID:AB_314194
Anti-human CD3	BioLegend	Cat# 300414, RRID:AB_314068
Anti-human CD7	BioLegend	Cat# 343102, RRID:AB_1659214
Anti-human CD5	BioLegend	Cat# 300602, RRID:AB_314088
Anti-human CD62L	BD Biosciences	Cat# 555541, RRID:AB_395925
Anti-human CD48	BioLegend	Cat# 336702, RRID:AB_1227561
Anti-human CD68	Thermo Fisher Scientific	Cat# 14-0688-82, RRID:AB_11151139
Anti-human CD20	BioLegend	Cat# 302302 RRID:AB_314250
Anti-human CD19	BioLegend	Cat# 302214, RRID:AB_314244
Anti-human CD66a/c/e	BioLegend	Cat# 342302, RRID:AB_1626265
Anti-human CLA	BioLegend	Cat# 321302, RRID:AB_492894
Anti-human HLA-DR	BioLegend	Cat# 307602, RRID:AB_314680
Anti-human CD115 (c-fms)	Thermo Fisher Scientific	Cat# 14-1159-82, RRID:AB_493929
Anti-human CD64	BD Biosciences	Cat# 555525, RRID:AB_395911
Anti-human CD1c	BioLegend	Cat# 331502, RRID:AB_1088995
Anti-human FceR1 alpha	Thermo Fisher Scientific	Cat# 16-5899-82, RRID:AB_657792
Anti-human SEPP1	Abcam	Cat# ab109514, RRID:AB_10862662
Anti-human CD123	BD Biosciences	Cat# 554527, RRID:AB_395455
Anti-human CD163	BioLegend	Cat# 333602, RRID:AB_1088991
Anti-human CXCR3 (CD183)	BD Biosciences	Cat# 557183, RRID:AB_396594
Anti-human CD56	BD Biosciences	Cat# 559043, RRID:AB_397180
Anti-human CD226	BioLegend	Cat# 338302 RRID:AB_1279155
Anti-human CD169	BioLegend	Cat# 346002 RRID:AB_2189031

(*Continued on next page*)

Continued

REAGENT or RESOURCE	SOURCE	IDENTIFIER
Anti-human SIRP1a (CD172a/b)	BioLegend	Cat# 323802, RRID:AB_830701
Anti-human CD369 (Dectin-1/CLEC7A)	BioLegend	Cat# 355402, RRID:AB_2561530
Anti-human CD1a	BioLegend	Cat# 300102, RRID:AB_314016
Anti-human CD141	BD Biosciences	Cat# 559780, RRID:AB_397321
Anti-human CD86	BD Biosciences	Cat# 555663, RRID:AB_396017
Anti-human CX3CR1	BioLegend	Cat# 355702, RRID:AB_2561726
Anti-human CD26	BioLegend	Cat# 302702, RRID:AB_314286
Purified anti-phycoerythrin (PE)	BioLegend	Cat# 408102, RRID:AB_2168924
Anti-human CD88 (C5aR)	BioLegend	Cat# 344304, RRID:AB_2067175
Anti-human CD34	BioLegend	Cat# 343502, RRID:AB_1731898
Anti-Human Mer	R and D Systems	Cat# MAB8912, RRID:AB_2143588
Anti-human CD39	BioLegend	Cat# 328202, RRID:AB_940438
Anti-human CD206	BioLegend	Cat# 321102, RRID:AB_571923
Anti-human CD11c	BD Biosciences	Cat# 555390, RRID:AB_395791
Anti-human CD11b	BioLegend	Cat# 301312, RRID:AB_314164
Anti-human CD16	Fluidigm	Cat# 3209002B
Biological Samples		
Buffy coats	University Hospital Bonn	N/A
Human Perfusates of lung transplant recipients	Hannover Medical School	N/A
Chemicals, Peptides, and Recombinant Proteins		
VLE-RPMI	Biochrom	Cat# FG1415
GlutaMAX	GIBCO	Cat# 35050-061
Penicillin-Streptomycin	GIBCO	Cat# 15140-122
Sodium Pyruvate	GIBCO	Cat# 11360-039
Fibronectin, human	Alfa Aesar	Cat# J64560
Fibronectin	Sigma-Aldrich	Cat# F1141
Pancoll	PAN-Biotech	Cat# P04-60500
FcR Blocking Reagent, human	Miltenyi Biotec	Cat# 130-059-901
rhMCSF	Immunotools	Cat# 11343115
rhGMCSF	Immunotools	Cat# 11343125
rhIL4	Immunotools	Cat# 11340045
Cisplatin	Sigma-Aldrich	Cat# 1134357
DNA (Iridium interchelator)	Fluidigm	Cat# 201192A
Paraformaldehyde	Electron Microscopy Sciences	Cat# 30525-89-4
Perm Buffer	BioLegend	Cat# 425401
EQ Four Element Calibration Beads	Fluidigm	Cat# 201078
Fluoresbrite YG Beads	Polysciences	Cat# 17154
DAPI	Sigma-Aldrich	Cat# 10236276001
Fluoroshield	Sigma-Aldrich	Cat# F6182-20ML
PEG 3500	Sigma-Aldrich	Cat# P3640
siRNA Buffer	Dharmacon	Cat # B-002000-UB-100
LPS	InvivoGen	Cat# tlrl-3pelps
IFN-γ	Immunotools	Cat# 11343536
CL097	InvivoGen	Cat# tlrl-c97
Flagellin	InvivoGen	Cat# tlrl-epstfla
Oligomycin A	Sigma-Aldrich	Cat# 75351
Fluoro-carbonyl cyanide phenylhydrazone	Tocris	Cat# 453
Rotenone	Sigma-Aldrich	Cat# R8875
Antimycin	Sigma-Aldrich	Cat # A8674

(*Continued on next page*)

Continued

REAGENT or RESOURCE	SOURCE	IDENTIFIER
Glucose	Sigma-Aldrich	Cat# G7021
Glutamine	Sigma-Aldrich	Cat# 59202C
2-Deoxyglucose	Sigma-Aldrich	Cat# D8375
Bicarbonate-free RPMI	Sigma-Aldrich	Cat# D5030
TRIzol Reagent	Thermo Fisher Scientific	Cat# 15596026
Critical Commercial Assays		
CD14 Microbeads, human	Miltenyi Biotec	Cat# 130-042-401
Nuclear Extract Kit	Active Motif	Cat# 40010
miRNeasy Micro kit	QIAGEN	Cat# 217084
Transcriptor First Strand cDNA Synthesis Kit	Roche	Cat# 04379012001
LEGENDplex	BioLegend	N/A
CyQuant Cell Proliferation Assay kit	ThermoFisher	Cat# C7026
TargetAmp-Nano Labeling Kit	Epicenter	Cat# TAN091096
TruSeq RNA Sample Preparation Kit v2	Illumina	Cat# RS-122-2001
Kapa library quantification kit	Kapa Biosystems	Cat# 07-KK4852-01
SPRIselect reagent kit	Beckman Coulter	Cat# B23319
Human CCL2/MCP-1 DuoSet ELISA	R and D Systems	Cat# DY279
Human CCL22/MDC DuoSet ELISA	R and D Systems	Cat# DY336
LYNX Rapid RPE Antibody Conjugation Kit	Bio-Rad Laboratories	Cat# LNK021RPE
Deposited Data		
Microarray and RNA-Seq data	This Paper	GEO: GSE96719
Experimental Models: Primary Cells		
Human primary cell isolates	This paper	N/A
Human primary cell culture	This paper	N/A
Experimental Models: Organisms/Strains		
GFP-expressing *Pichia Pastoris*	This Paper	N/A
Oligonucleotides		
siRNA targeting sequence: hNCOR2 #1 UGGUUUACAU GUCGACUAA	This Paper	N/A
siRNA targeting sequence: hNCOR2 #2 UGGUUUACAU GUUGUGUGA	This Paper	N/A
siRNA targeting sequence: hNCOR2 #3 UGGUUUACAU GUUUUCUGA	This Paper	N/A
siRNA targeting sequence: hNCOR2 #4 UGGUUUACAU GUUUUCCUA	This Paper	N/A
siRNA non-targeting sequence: control #1 UGGUUUACA UGUCGACUAA	This Paper	N/A
siRNA non-targeting sequence: control #2 UGGUUUACA UGUUGUGUGA	This Paper	N/A
siRNA non-targeting sequence: control #3 UGGUUUACA UGUUUUCUGA	This Paper	N/A
siRNA non-targeting sequence: control #4 UGGUUUACA UGUUUUCCUA	This Paper	N/A
Primer: NCOR2 Forward: GCGAGGTCTCCCTGAGTCTT	This Paper	N/A
Primer: NCOR2 Reverse: CCAGTCCTCGTCATCAGCTC	This Paper	N/A
NCOR2 Probe	Roche	Cat# Universal Taqman ProbeLibrary #16
Primer: CD209 Forward: CCAGGTGAAGCGGTTACTTC	This Paper	N/A
Primer: CD209 Reverse: GCTCGTCGTAATCAAAAGTGC	This Paper	N/A
CD209 Probe	Roche	Cat# Universal Taqman ProbeLibrary #68
Primer: GAPDH Forward: AGCCACATCGCTCAGACAC	This Paper	N/A

(*Continued on next page*)

Continued		
REAGENT or RESOURCE	SOURCE	IDENTIFIER
Primer: GAPDH Reverse: GCCCAATACGACCAAATCC	This Paper	N/A
GAPDH Probe	Roche	Cat# Universal Taqman ProbeLibrary #60
Primer: ACTB Forward 1: TGGTGGGCATGGGTCAGA	This Paper	N/A
Primer: ACTB Reverse 1: GTACATGGCTGGGGTGTTGA	This Paper	N/A
Primer: ACTB Forward 2: AACAAGATGAGATTGGCA	This Paper	N/A
Primer: ACTB Reverse 2: GACCAAAAGCCTTCATACAT	This Paper	N/A
Software and Algorithms		
FlowJo	Tree Star	RRID:SCR_008520
Partek Genomics Suite	Partek Inc.	N/A
Cytoscape	Cytoscape	N/A
Cytofkit	Bioconductor	N/A
Gene Set Enrichment Analyze	Broad Institute	N/A

CONTACT FOR REAGENT AND RESOURCE SHARING

Further information and requests for resources and reagents should be directed and will be fulfilled by the Lead Contact, Andreas Schlitzer (andreas.schlitzer@uni-bonn.de).

EXPERIMENTAL MODEL AND SUBJECT DETAILS

Human primary cell isolation

Buffy coats were obtained from healthy donors (University hospital Bonn, local ethics vote 203/09) after written consent was given according to the Declaration of Helsinki. Peripheral blood mononuclear cells (PBMC) were isolated by Pancoll (PAN-Biotech) density centrifugation from buffy coats. CD14-, CD56-, CD4- and CD19-specific MACS beads (Miltenyi Biotec) were used for the enrichment of $CD14^+$ MO, $CD56^+$ NK cells, $CD4^+$ T cells and $CD19^+$ B cells, respectively. Lung-derived myeloid cells were isolated from human perfusates of lung transplant recipients with informed consent and immediately sorted for $CD45^+Lin^-HLA\text{-}DR^{hi}$ cells using the FACS Fusion cell sorter (BD, USA).

Human primary cell culture

$CD14^+$ MO were cultured in RPMI1640 medium supplemented with 10% FCS, 1% Penicillin-Streptomycin, 1% Sodium pyruvate and 1% Glutamax (GIBCO) for 3 days. $CD14^+$ MO were differentiated into Mo-M-CSF or Mo-GM-CSF in the presence of 100 IU/ml rhM-CSF or 800 IU/ml rhGM-CSF, respectively. Mo-GM-$CSF^{IL\text{-}4}$ were generated by the addition of 800 IU/ml rhGM-CSF and 500 IU/ml rhIL4 and were incubated for up to 6 days. All cytokines were purchased from Immunotools.

METHOD DETAILS

Flow cytometry

Cells were washed with ice cold PBS. After FcR blockage (Miltenyi, Germany), cells were stained with the respective antibodies in PBS supplemented with 0.5% FCS, 2.5 mM EDTA for 20min at 4°C. Following antibodies were purchased from Biolegend (USA): CD19 (HIB19), CD11b (CBRM1/5), CD23 (EBVCS-5), CD14 (M5E2); R&D: VSIG4 (polyclonal), MARCO (polyclonal), CCR7 (150503); Becton Dickinson (BD, USA): CD56 (B159), HLA-DR (L243), CD209 (DCN46), CD226 (DX11); Data acquisition was performed with a LSR II (BD). Analyses were performed with FlowJo software (Tree Star).

Mass cytometry

Following culture, cells were washed with PBS (GIBCO, Life Technologies, Carlsbad, CA) and stained with cisplatin (Sigma-Aldrich, St Louis, MO). Then, cells were washed with PBS containing 4% FBS and 0.05% Sodium azide and fixed with 2% paraformaldehyde (PFA; Electron Microscopy Sciences, Hatfield, PA) overnight. Cells were permeabilized (1X perm buffer (Biolegend, San Diego, CA)) and stained with metal-conjugated antibodies (Table S7) intracellularly. Then cells were washed, metal barcoded and resuspended in water. EQ Four Element Calibration Beads (Fluidigm Corporation, South San Francisco, CA) were added at a concentration of 1% prior to acquisition. Cells were acquired and analyzed using a CyTOF1 Mass cytometer. The data was normalized and events with parameters having zero values were replaced with a randomized value between 0 and −1.

Mass cytometry data analysis

Normalized MC data was exported in .fcs format and pre-processed in FlowJo Version 9.9.4 (Tree Star Inc). Pre-processing included removal of cell debris (Iridium-191/193 DNA intercalating fraction) and dead cells (cisplatin^{+}). Myeloid CD45^{+}lin(CD3+CD7+CD15+CD19+CD20+CD56)$^{-}$ cells were exported and used for analysis. Downstream analysis was performed using the Cytofkit R package. For comparison of the different differentiating conditions data from 1000 myeloid cells was randomly sampled per donor and condition (3 donors; 12,000 cells in total) and autoLgcl-transformed including expression values for 36 surface markers (CD45, CD14, CD5, CD62L, CD48, CD68, CD66ace, CLA, HLA-DR, CD115, CD64, CD1c, FceR1, SEPP1, CD123, CD163, CXCR3, CD226, CD169, SIRP1a, Dectin1a, CD1a, CD141, MARCO, CD86, CX3CR1, CD206, VSIG4, CD88, CD34, MerTK, CD39, CD26, CD11c, CD11b, CD16). Detailed analysis on the Mo-GM-CSF^{IL-4} condition was based on 3500 cells from one individual. To define clusters of cell subpopulations, PhenoGraph was used. Points representing individual cells in the *t*-SNE plots were color-coded to illustrate amount of protein expression or affiliations to clusters, treatment conditions or donors, respectively. Alternatively, the gplots R package was used to generate heatmaps of marker expression of individual cells or mean values over identified cell clusters. Dendrograms represent hierarchical clustering results based on the Euclidean distance measure.

Uptake of fluorescent microbeads or yeast

Cells were incubated either with fluorescent monodispersed polystyrene microspheres (1 μm diameter, Fluoresbrite YG Beads, Polysciences) or yeast (GFP-expressing *Pichia Pastoris*) in a cell-to-bead ratio of 1/10 for 4h or 60 min at 37°C, respectively. Afterward, cells were harvested, washed and bead/yeast uptake was analyzed by flow cytometry using an LSR II (BD). Negative control samples were kept at 4°C. Data analysis was performed using FlowJo software (Tree Star).

Immuno blot

The Nuclear Extract Kit (Active Motif) was used to fractionate the cytosolic and nuclear proteins, fractions were separated by SDS-PAGE and transferred onto a nitrocellulose membrane (Amersham) by wet blotting. Probing was performed using hNCOR2 (Abcam), β-Tubulin (Cell Signaling) and Lamin A/C (Active Motif) antibodies. For MMP12 protein detection, the cytosolic whole protein fractions (Dignam extraction) were separated by SDS-PAGE and transferred onto a nitrocellulose membrane (Amersham) by semi-dry blotting. Probing was performed by using MMP-12 (R&D) and β-actin antibodies. Signal detection and analysis was performed on the LI-COR Odyssey system. Cell compartment separation efficiency was validated by enrichment of cytosolic proteins, such as β-Tubulin or nuclear proteins such as Lamin A/C. Signal expression values of hNCOR2 and MMP-12 were calculated in semiquantitative relation to the signal expression values of β-Tubulin, Lamin A/C and β-actin following the equation target/reference.

Migration assay

Migration was analyzed in μ-Slide 8 well chambered coverslips (Ibidi) coated with 50μg/ml human fibronectin (Alfa Aesar). 0.7×10^5 cells in 300μl VLE-RPMI (Biochrom) were seeded in each well. Live cell imaging of adherent cells was performed at 37°C and 5% CO_2 using a fully automated inverted Olympus Fluoview 1000 confocal microscope equipped with motorized xyz stage. Cell motility was monitored over a period of 3h by capture of differential interference contrast images every 5min with a 0.40 UPLAPO 10x Objective (Olympus). Migration parameters were calculated using the Manual Tracking and Chemotaxis Tool plugins in ImageJ.

Confocal microscopy and data analysis

Cover slides were coated with fibronectin (Sigma Aldrich) prior to the seeding of Mo-GM-CSF or Mo-GM-CSF$^{IL-4(0-144h)}$. After an incubation time of 3h at 37°C, adherent cells were washed with PBS and fixed in pre-cooled methanol at −20°C for 10min. Subsequently slides were washed twice in PBS and blocked with 5% BSA in PBS for 30min. Staining for NCOR2 and tubulin was done using αNCOR2 antibody (ab24551, Abcam) at 4°C overnight or using tubulin antibody (MAB1864 (Chemicon)) at room temperature for 45min. For visualization slides were stained with anti-rabbit-Alexa488 (A11034, Life Technologies) or anti-rat Alexa555 (A21434, Invitrogen) and DAPI (Sigma Aldrich) at room temperature for 45min. Slides were mounted with fluoroshield (ImmunoBioScience) plus 1% DABCO and confocal image acquisition of the medial nuclear region was performed using an inverted Olympus Fluoview 1000 confocal microscope equipped with a Plan Apochromat 60x, NA1.4 oil immersion objective (Olympus) and 405nm/488nm/543nm laser lines. Quantification of mean intensity of the green fluorescence in the nuclear region was performed using Imaris 7.6.5 (Bitplane).

NCOR2 siRNA silencing

24h prior to the experiment, the nanostraw cargo chamber was washed 3x with 10μl of 0.5% PEG 3500 (P3640, sigma) in PBS and equilibrated; chambers were equilibrated with 100μl RPMI 1640 media. CD14^{+} human MO were resuspended in RPMI 1640 with supplements (10% FCS, 1% Pen/Step, 1% GlutaMax and 1% NaPyruvat) and activated. siRNA solutions were prepared in 1x siRNA Buffer (Dharmacon). hNCOR2 siRNA (Dharmacon) ON Target Plus pool was used for silencing (siRNA sequences targeting hNCOR2: 1. UGGUUUACAUGUCGACUAA, 2. UGGUUUACAUGUUGUGUGA, 3. UGGUUUACAUGUUUUCUGA, 4. UGGUUUACAUGUUUUCCUA; non-targeting control siRNA sequences: 1. UGGUUUACAUGUCGACUAA, 2. UGGUUUACAUGUUGUGUGA, 3. UGGUUUACAUGUUUUCUGA, 4. UGGUUUACAUGUUUUCCUA). After removing the equilibration media the tubing system was filled with the siRNA solution or PBS. Subsequently, the cell suspension was filled into the chamber and incubated for 72h at 37°C and 5%CO_2. After 72h, cells were directly lysed within the chambers by adding Trizol. qRT-PCR was performed to check silencing efficiency.

Real time PCR

Total RNA was isolated with the miRNeasy Micro kit (QIAGEN) and analyzed with the High Sensitivity RNA Tapestation system (Agilent). cDNA synthesis was prepared using the Transcriptor kit (Roche). qRT-PCR was performed using a Lightcycler 480 system (Roche). Primer sequences: NCOR2 (F, GCGAGGTCTCCCTGAGTCTT; R, CCAGTCCTCGTCATCAGCTC); CD209 (F, CCAGGTGAA GCGGTTACTTC; R, GCTCGTCGTAATCAAAAGTGC); GAPDH (F, AGCCACATCGCTCAGACAC; R, GCCCAATACGACCAAATCC). Expression values were calculated by the comparative C_T method. GAPDH served as internal control gene. FC is presented relative to amount of expression in MO

Cell stimulation

Mo-M-CSF, Mo-GM-CSF, Mo-GM-CSF$^{IL-4(0-72h)}$ or Mo-GM-CSF$^{IL-4(0-144h)}$ cells were stimulated overnight under the following conditions: Media, 100ng/ml LPS ultrapure, 100ng/ml LPS ultrapure + 1000U/ml IFNγ, 1μg/ml CL097, 100ng/ml flagellin. Supernatants were harvested 19h after stimulation and stored at -80°C.

Cytokine measurement

Cytokines were measured using LEGENDplex (Biolegend, USA). Diluted cell culture supernatants were incubated for 2 hours with the beads and detection antibodies, followed by 30min incubation with SA-PE. After washing, beads were resuspended in washing buffer and acquired using a LSRII flow cytometer (BD). Data were analyzed with the LEGENDplex Data Analysis Software; concentration values were exported to Excel and visualized in R.

Oxygen consumption rate (OCR) and ECAR measurements

OCR and ECAR were determined using a XF-96 Extracellular Flux Analyzer (Seahorse Bioscience). For ECAR analysis the media was changed to bicarbonate-free RPMI supplemented 10mM glucose, 1mM pyruvate & 2 mM glutamine 1h prior to the assay and the plate was kept in a non-carbonated incubator. Measurements were performed under basal conditions and after the sequential addition of final 1μM oligomycin A, 1.5μM FCCP (fluoro-carbonyl cyanide phenylhydrazone) and 0.5μM rotenone & antimycin each. For ECAR analysis the media was changed to bicarbonate free RPMI supplemented 2 mM glutamine 1h prior to the assay and the plate was kept in a non-carbonated incubator. Measurements were performed under basal conditions and after the sequential addition of final 10 mM glucose, 1μM oligomycin and 100 mM 2-Deoxyglucose. OXPHOS was calculated as (basal OCR – OCR after rotenone & antimycin treatment), ATP production was calculated as (basal OCR – OCR after oligomycin A treatment), maximal respiration was calculated as (OCR after FCCP treatment – OCR after rotenone & antimycin treatment), glycolysis was calculated as (basal ECAR – ECAR after 2-Deoxyglucose treatment), glycolytic capacity was calculated as (ECAR after oligomycin A treatment – ECAR after 2-Deoxyglucose treatment. All reagents were purchased from Sigma, except FCCP was purchased from Tocris. ECAR and OCR raw data was normalized to DNA content using the CyQuant Assay kit (Thermo Fisher).

Microarray data generation

Up to 5 × 10^6 cells were harvested and lysed in TRIzol (Invitrogen) and RNA was isolated and concentration and purity was assessed using a NanoDrop 1000 UV-Vis Spectrophotometer (Thermo Scientific). Subsequently, the TargetAmp-Nano Labeling Kit for Illumina Expression BeadChip (Epicenter) was utilized to generate biotin labeled anti-sense RNA (cRNA) according to the manufacturer's protocol. As a quality control, 100 ng cRNA were reverse transcribed to cDNA and a PCR specific for *ACTB* amplification was performed. For expression profiling, 750 ng cRNA were hybridized to Human HT-12v3 BeadChip arrays (Illumina), stained and imaged on an Illumina iScan system.

RNA-sequencing

Total RNA was converted into libraries of double stranded cDNA molecules as a template for high throughput sequencing following the manufacturer's recommendations using the Illumina TruSeq RNA Sample Preparation Kit v2. Shortly, mRNA was purified from 100 ng of total RNA using poly-T oligo-attached magnetic beads. Fragmentation was carried out using divalent cations under elevated temperature in Illumina proprietary fragmentation buffer. First strand cDNA was synthesized using random oligonucleotides and SuperScript II. Second strand cDNA synthesis was subsequently performed using DNA Polymerase I and RNase H. Remaining overhangs were converted into blunt ends via exonuclease and polymerase activities and enzymes were removed. After adenylation of 3′ ends of DNA fragments, Illumina PE adaptor oligonucleotides were ligated to prepare for hybridization. DNA fragments with ligated adaptor molecules were selectively enriched using Illumina PCR primer PE1.0 and PE2.0 in a 15 cycle PCR reaction. Size-selection and purification of cDNA fragments with preferentially 200 bp in length was performed using SPRIBeads (Beckman-Coulter). Size-distribution of cDNA libraries was measured using the Agilent high sensitivity DNA assay on a Bioanalyzer 2100 system (Agilent). cDNA libraries were quantified using KAPA Library Quantification Kits (Kapa Biosystems). After cluster generation on a cBot, a 75 bp single-read run was performed on a HiSeq1500.

Primary handling of microarray datasets

Three major microarray datasets were generated and pre-processed, which were later on combined with each other and/or with publicly available data. The first dataset (pre-dataset 1) contains $CD14^+$ MO, B, T and NK cells, as well as different types of MO-derived cells (Mo-M-CSF, Mo-GM-CSF, Mo-GM-CSF$^{IL-4(0-72h)}$ and Mo-GM-CSF$^{IL-4(0-144h)}$) from the blood of healthy human donors.

The second dataset (pre-dataset 2) is composed of the same $CD14^+$ monocyte and MO-derived cell samples (Mo-GM-CSF, Mo-GM-$CSF^{IL\text{-}4(0\text{-}72h)}$ and Mo-GM-$CSF^{IL\text{-}4(0\text{-}144h)}$), but Mo-GM-$CSF^{IL\text{-}4(72\text{-}144h)}$ in addition. All corresponding samples were hybridized to Human HT12-V3 Beadchips (Illumina) and scanned on an Illumina iScan system. The third dataset (pre-dataset 3) contains $CD14^+$ MO and $CD45^+lin^-MHCII^{hi}$ cells, which were sorted from blood and lungs of healthy human donors, respectively. The corresponding samples were hybridized to Human HT12-V4 Beadchips (Illumina) and scanned on an Illumina HiScanSQ system. The raw intensity data of all three datasets was pre-processed by the Gene Expression tool v1.9.0 in GenomeStudio V2011.1 (Illumina) independently. Values for missing bead types were imputed, but no prior normalization was performed at this step. This revealed 48,803 probesets for the HT12-V3 datasets, and 47,323 probesets for the HT12-V4 dataset. All datasets were exported using the Partek Report Plugin 2.16 (Partek) and imported into Partek® Genomics Suite software®, version 6.6©; 2017 (PGS), where they were further processed.

Handling of publicly available data

From a publicly available dataset (GEO: GSE35457, (Haniffa et al., 2012)) the raw data of $CD14^+$ and $CD14^+CD16^+$ monocyte samples, as well as of $CD1c^+$ and $CD141^+$ DC samples from blood of up to four donors (I, II, V and VI) were used. The data was generated on Human HT12-V4 BeadChips and contained 47,323 probesets, where values for missing bead types were imputed. Since all other in-house datasets contained $CD14^+$ MO only, the raw expression values for the $CD14^+$ and $CD14^+CD16^+$ monocyte samples were averaged always for the same donor. In the end, the designation "$CD14^+$ monocyte" was kept. The resulting raw expression text file was imported into PGS as well.

To compare the MO-derived cells to inflammatory conditions, a dataset containing inflammatory Mac and dendritic cells, isolated from the ascites of ovarian cancer patients, as well as primary $BDCA1^+$ dendritic cells and $CD14^+CD16^-$ and $CD14^{dim}CD16^+$ MO was downloaded (GEO: GSE40484, (Segura et al., 2013)). The raw Affymetrix Human Gene 1.1 ST Array data was imported into PGS using RMA background correction, quantile normalization and log_2-transformation. For the inflammatory cells, only donors 1 to 4 were considered. Afterward, 12,067 present probes were determined requiring at least one group mean to be larger than 7 on log_2 scale. Of those, only 9,358 probes being annotated with a gene symbol were further considered. Finally, the probes were reduced to 8,612 present genes, by considering only the probe with the highest expression for a given gene. In order to link the Affymetrix data to the Illumina datasets of MO-derived cells, three different approaches were applied.

Probe matching between platforms

Using the Illumina Probe ID ("ILMN"), 39,423 common probesets were identified on both HT12-V3 and HT12-V4 arrays. All in-house datasets (pre-datasets 1-3) and the dataset from Haniffa et al. were filtered down to those probes to enable dataset assembly and comparability. Since for some of the probes different gene name synonyms were used in the two different platform files, the annotation presented in the newer file (HT12-V4) was used for all filtered datasets later on.

Assembly of datasets and pre-processing

Based on the identified common probes, four different datasets were generated. The first one (dataset 1) was assembled by combining the complete pre-datasets 1 and 2, as well as the dataset from Haniffa et al. (GEO: GSE35457, (Haniffa et al., 2012)). The second (dataset 2) contains the pre-dataset 1 without the B, T and NK cells, plus pre-dataset 2 and the dataset from Haniffa et al.. The raw expression values of both datasets were first quantile normalized and log_2-transformed independently. Afterward, using the $CD14^+$ blood MO as bridging samples, the batch effect introduced by the combination of the three different datasets was removed, again separately for datasets 1 and 2. The third dataset (dataset 3) was composed of $CD14^+$ MO, Mo-M-CSF, Mo-GM-CSF and Mo-GM-$CSF^{IL\text{-}4(0\text{-}72h)}$ from pre-dataset 1. The fourth (dataset 4) equals the filtered pre-dataset 3. Both datasets were quantile-normalized and log_2-transformed independently.

Primary data analysis and visualization

Each of the four datasets was filtered down to those probes being expressed across the corresponding dataset. To do so, the maximum of the group mean expression values was determined. Probes with a maximum expression lower than 7, again 7, 6.95 and 6.75 (all on log_2 scale) were excluded as not expressed from datasets 1, 2, 3 and 4, resulting in 21,250, 23,592, 18,318 and 18,857 present probes, respectively. The samples of each dataset were always visualized via principal component analysis (PCA) using the determined present probes as input. For all datasets, principal components (PCs) 1 (x axis) versus 2 (y axis) were displayed, and for datasets 1, 2 and 4 also other combinations of PCs are shown. Furthermore, the top 1000 most variable probes of datasets 1, 3 and 4 were visualized in the form of a heatmap, where rows and columns were ordered based on hierarchical clustering using Euclidean distance and average linkage. Based on the revealed row-wise clusters, the genes were grouped together for datasets 3 and 4, dependent on the cell type(s) they appeared as highly expressed. A color code was added to indicate the corresponding group-related cell types. Additionally, a few genes of each cluster were highlighted next to the heatmap. For datasets 1, 3 and 4, the top 1000 most variable probes were also used as input to calculate a pearson correlation coefficient matrix (PCCM), which were displayed as heatmaps as well. Also there, rows and columns were ordered based on hierarchical clustering using Euclidean distance and average linkage.

Identification of signature genes

Using the normalized and batch corrected dataset 2, differentially expressed probes (Fold-Change ≤ -2 or $> = 2$ and FDR-adjusted p value < 0.05 using a two-way ANOVA including the batch as a random effect) were determined for the three comparisons Mo-GM-CSF$^{IL-4(0-72h)}$ and Mo-GM-CSF$^{IL-4(0-144h)}$ versus CD14^{+} MO, Mo-GM-CSF versus CD14^{+} MO and Mo-M-CSF versus CD14^{+} MO. The three lists of probes being upregulated against CD14^{+} MO were intersected, and the same was done for the probes being downregulated compared to CD14^{+} MO. The probes were then reduced to single genes, by keeping the probe for a corresponding gene with the highest mean expression across the dataset. This resulted in 287 up- and 361 downregulated genes in MO-derived cells compared to CD14^{+} MO. Both lists were further filtered by keeping only those genes, for which the mean expression values of the four MO-derived cell populations did not differ by a Fold-Change > 1.7 for all pairwise comparisons. This ensured similar expression values in the MO-derived cells, and resulted in 184 up- and 279 downregulated genes compared to CD14^{+} MO. The expression patterns of those genes were displayed in the form of a heatmap, where rows and columns were ordered based on hierarchical clustering using Euclidean distance and average linkage. A few gene names are depicted next to the heatmap.

Linear support vector regression (SVR)

The 8,612 present genes were used as input for CIBERSORT (Newman et al., 2015) to create a signature matrix, which contained 230 genes representing the five different cell types of the inflammatory Affymetrix dataset. After excluding all genes, which did not appear within the Illumina dataset 2, 197 genes remained. In addition, the 23,592 present probes of dataset 2 were reduced to 16,618 present genes by keeping the probe with the highest mean expression for a given gene. Then, SVR in CIBERSORT was applied to predict the fractions of the five cell types (in the form of the generated and filtered signature matrix composed of 197 genes) within the gene expression profiles (present genes) of dataset 2. To reduce complexity, the Mo-GM-CSF$^{IL-4(0-72h)}$ and Mo-GM-CSF$^{IL-4(0-144h)}$ were considered as a single group (named Mo-GM-CSF$^{IL-4(0-72h/144h)}$) by calculating the mean expression across all replicates of the two groups. The resulting fractions were finally visualized in the form of stacked bar plots.

GSEA analysis

Using again the 8,612 present genes, DEG were determined between inflammatory DCs and CD14^{+}CD16^{-} MO, inflammatory DCs and CD14^{dim}CD16^{+} MO, inflammatory Mac and CD14^{+}CD16^{-} MO, as well as between inflammatory Mac and CD14^{dim}CD16^{+} MO by requiring a Fold-Change > 4 and an FDR-adjusted p value < 0.05 for all four comparisons using one way ANOVA. For each of the two comparisons inflammatory DCs versus CD14^{+}CD16^{-} MO and inflammatory Mac versus CD14^{+}CD16^{-} MO, specific genes were determined by excluding all genes appearing as differentially expressed in at least one of the other three comparisons. This resulted in 27 specific (not overlapping) genes for each of the two comparisons, respectively. Those two gene sets were then used as input for Gene Set Enrichment Analysis within PGS, using 1000 permutations and restricting the analysis to functional groups with more than 2 and fewer than 5000 genes. The enrichments were performed on the 16,618 present genes of dataset 2, for the pairwise comparisons CD141^{+} DC versus CD1c^{+} DC, Mo-GM-CSF$^{IL-4(0-72h/144h)}$ versus CD14^{+} monocyte, Mo-GM-CSF versus CD14^{+} monocyte, Mo-M-CSF versus CD14^{+} monocyte and CD45^{+}lin^{-}MHCII^{hi} versus CD14^{+} monocyte. Again here, all replicates from Mo-GM-CSF$^{IL-4(0-72h)}$ and Mo-GM-CSF$^{IL-4(0-144h)}$ were considered as a single group Mo-GM-CSF$^{IL-4(0-72h/144h)}$. The enrichment results were visualized within a scatterplot, with the normalized enrichment score (NES) on the x axis versus the FDR-corrected p value on the y axis.

Correlation of gene expression

As a third approach, the expression patterns of genes in the dataset of MO-derived cells were directly correlated to those in the dataset of inflammatory DCs and Mac. In both datasets, 6,959 common present genes (according to the gene symbols) were identified. By considering the results observed after the application of linear SVR, both datasets were divided into four comparable groups. Within the Illumina dataset 2 containing MO-derived cells, the four generated groups were: 1) Mo-GM-CSF$^{IL-4(0-72h/144h)}$, 2) CD1c^{+} and CD141^{+} DCs, 3) Mo-GM-CSF and Mo-M-CSF as well as 4) CD14^{+} MO and CD45^{+}lin^{-}MHCII^{hi} cells. The corresponding four groups in the Affymetrix inflammatory cell dataset were: 1) inflammatory DCs, 2) BDCA1^{+} DCs, 3) inflammatory Mac as well as 4) CD14^{+}CD16^{-} and CD14^{dim}CD16^{+} MO. Finally, for each of the 6,959 genes a Pearson correlation value was calculated between the profile consisting of the four group mean expression values of Illumina dataset 2 and the corresponding profile of the Affymetrix dataset. As a first filtering step, only those genes were kept, which had a correlation value > 0.4. Within a second filtering step, certain Fold-Changes were required. For the Illumina MO-derived cell dataset, Fold-Changes were calculated for Mo-GM-CSF$^{IL-4(0-72h/144h)}$ compared to all other cell types of this dataset, and for the Affymetrix inflammatory cell dataset, Fold-Changes were determined for inflammatory dendritic cells compared to all other cell types within the dataset. For both datasets, only those genes were kept, for which all Fold-Changes were > 1.3. This resulted in 25 genes, which were specifically expressed in both Mo-GM-CSF$^{IL-4(0-72h/144h)}$ and inflammatory dendritic cells, but overall lower expressed in all other investigated cell types. The same approach was applied to find commonly expressed genes in inflammatory Mac as well as in Mo-GM-CSF and Mo-M-CSF. Fold-Changes were calculated for inflammatory Mac compared to all other cell types of the Affymetrix dataset. For each gene of the Illumina dataset, Fold-Changes were determined between the minimum of the two group mean expressions of Mo-GM-CSF and Mo-M-CSF and all other cell types (Mo-GM-CSF$^{IL-4(0-72h/144h)}$, CD1c^{+} and CD141^{+} DCs, CD14^{+} MO) of this dataset. Of the genes having a correlation score > 0.4, only 44 specific ones remained after excluding those, which did not have a Fold-Change > 1.3 for all comparisons within both datasets. Additionally, genes appearing in the final annotation file of 39,423 common probes of dataset 2 with multiple transcript variants - and

therefore with potentially different expression patterns depending on the variant, of which only one was chosen based on the highest expression - were removed. The expression patterns of the remaining genes of both lists were visualized in the form of heatmaps, and the corresponding Pearson correlation values were displayed within bar plots next to the heatmaps.

Identification of subset specific genes

Using dataset 3, the 18,318 present probes were further reduced to 13,452 present genes by keeping the probe with the highest expression across the dataset for a given gene. To enable the identification of important surface markers, a comprehensive collection of important surface marker genes was assembled. For this, corresponding lists were downloaded from two different sources. First, a list of genes encoding membrane proteins was downloaded from the Human Protein Atlas, available at http://www.proteinatlas.org/search/protein_class:Predicted+membrane+proteins+AND+NOT+protein_class:Predicted+secreted+proteins. Second, a list of genes encoding surface proteins was obtained from SurfaceDB (de Souza et al., 2012), available at http://www.bioinformatics-brazil.org/surfaceome/home-. The union of both lists was generated and contained 5,582 gene symbols. Of those, 2,726 genes were found among the 13,452 present genes, which were then considered to represent the present surfaceome of dataset 3. To find surface markers being specifically expressed in one of the three MO-derived cell types (Mo-M-CSF, Mo-GM-CSF and Mo-GM-CSF$^{IL\text{-}4(0\text{-}72h)}$), Fold-Changes for all pairwise comparisons including the ones against CD14^{+} MO were calculated. A marker was defined as condition specific, if all Fold-Changes against the three other cell types were larger than 2. This resulted in 19, 19 and 54 specific genes for Mo-M-CSF, Mo-GM-CSF and Mo-GM-CSF$^{IL\text{-}4(0\text{-}72h)}$, respectively. For visualization, those lists were further filtered. Genes belonging to the MHCI and II complexes (HLA-) were excluded since their specificity might be different in other individuals. Additionally, again here genes appearing in the final annotation file of 39,423 common probes with multiple transcript variants were removed. The remaining genes were displayed in the form of a heatmap, where the rows were ordered decreasingly based on the expression patterns in the three-different MO-derived cell types.

Co-expression network analysis

The union of genes being differentially expressed (Fold-Change > 2 or < −2 and FDR-adjusted p value < 0.05 using one way ANOVA) between each of the three types of MO-derived cells compared to CD14^{+} MO of dataset 3 were imported into BioLayout Express 3D version 3.3. Requiring a correlation of at least 0.93 to at least one other gene (based on the anti-log$_2$-expression profiles), a co-expression network was generated. Smaller networks containing less than 5 genes were excluded, which resulted in a single network containing 2,086 genes. For each of the four cell types, the Fold-Change of the respective cell type compared to the overall mean was mapped onto the network and displayed in colors ranging from blue (negative Fold-Change) over white to red (positive Fold-Change) using the App Enrichment Map v2.0.1 in Cytoscape v3.2.0. Based on the Fold-Change patterns, the networks were divided into four main clusters, where each cluster represents one of the four cell types, respectively. Based on dataset 4, another co-expression network was generated to investigate the relationship of Mo-GM-CSF$^{IL\text{-}4(72\text{-}144h)}$ to the other MO-derived cell types and CD14^{+} MO. As input, 13,691 present genes were used, which were obtained by filtering the 18,857 probes in the same ways as previously described. The network was generated in BioLayout and finally visualized using Cytoscape. Two samples (nodes) are connected, if the correlation value calculated between their expression profiles was at least 0.9.

Prediction of master regulators

To predict master regulators for each of the four cell types, the Cytoscape App iRegulon v1.3 was applied on those genes of the cell type specific clusters identified within the co-expression network, which were upregulated in the corresponding cell type compared to the overall mean with a Fold-Change > 1.5. The prediction was performed by considering "20kb centered around TSS" as "Putative regulatory region," by using" No motif collection" at "Motif collection," and by setting the "Enrichment Score threshold" to 1.5. The resulting lists were filtered afterward to keep only those predicted regulators, which reached a normalized enrichment score (NES) > = 3 and which were identified in only one of the four cell types after the previous filtering step.

Transcriptional regulator Co-expression network

Among the 13,452 present genes of dataset 3, 587 transcriptional regulators including transcription factors, co-factors and histone modifiers were identified (Fulton et al., 2009). Those were imported into BioLayout, and by requiring a correlation of at least 0.83 to at least one other regulator, a co-expression network was generated. After excluding smaller networks containing less than 4 regulators, a single network composed of 411 transcriptional regulators remained. For each of the four cell types, the Fold-Change of the respective cell type compared to the overall mean was mapped onto the network using Enrichment Map in Cytoscape. According to the resulting patterns, cell type specific clusters of upregulated regulators were generated. Additionally, those of the previously predicted master regulators were marked in these clusters, which were part of the network. Among the 13,691 present genes of dataset 4, 585 transcriptional regulators were found (Fulton et al., 2009). The expression profiles of those were imported into BioLayout, and the final co-expression network contained 267 regulators after requiring a correlation of at least 0.85 between a gene and at least one other gene and excluding resulting smaller networks containing less than 3 regulators. Within Cytoscape using EnrichmentMap, the Fold-Changes comparing Mo-GM-CSF versus CD14^{+} MO as well as Mo-GM-CSF$^{IL\text{-}4(0\text{-}72h)}$ compared to CD14^{+} MO were mapped onto the network, ranging from blue (Fold-Change < -2) over white to red (Fold-Change > 2). According to the resulting patterns, a Mo-GM-CSF$^{IL\text{-}4(0\text{-}72h)}$ specific cluster of upregulated regulators was identified. Additionally, regulators among the 585 present ones being specifically upregulated in Mo-GM-CSF$^{IL\text{-}4(0\text{-}72/144h)}$ were determined by requiring all of the following conditions: Fold-Change

Mo-GM-CSF$^{IL-4(0-72h)}$ versus CD14^{+} Monocyte > 1.5, Fold-Change Mo-GM-CSF$^{IL-4(0-144h)}$ versus CD14^{+} Monocyte > 1.5, Fold-Change Mo-GM-CSF$^{IL-4(0-72h)}$ versus Mo-GM-CSF > 1.5, Fold-Change Mo-GM-CSF$^{IL-4(0-144h)}$ versus Mo-GM-CSF > 1.5 and Fold-Change Mo-GM-CSF versus CD14^{+} Monocyte > 1. The expression profiles of the remaining 7 transcriptional regulators were visualized in the form of a heatmap, were the rows were ordered decreasingly according to expression values in Mo-GM-CSF$^{IL-4(0-72h)}$. Those of the seven, which were also part of the co-expression network of transcriptional regulators, were highlighted within the network.

Expression profiles of pattern recognition receptors

Lists were collected of human gene symbols encoding Toll-like, NOD-like, RIG-I-like and C-type lectin receptors, as well as proteins belonging to the inflammasome. Only those of them were displayed in the form of barplots, for which the Fold-Change for at least one of the three MO-derived cell types compared to CD14^{+} MO was > = 2 or ≤ -2. The bars represent the Fold-Change between the mean values of each MO-derived cell type compared to the mean of CD14^{+} MO. Additionally, the Fold-Changes between the single replicate values compared to the mean of CD14^{+} MO are added as dots into the corresponding bar.

Comparison of Mo-M-CSF and Mo-GM-CSF cells

Mo-M-CSF and Mo-GM-CSF cells were compared both directly and indirectly. First, differentially expressed probes (Fold-Change > 2 or < -2 and FDR-adjusted p value < 0.05 using one way ANOVA) were determined between Mo-M-CSF and CD14^{+} MO as well as between Mo-GM-CSF and CD14^{+} MO. Probes were reduced to single genes by keeping the probe with the highest expression. The remaining lists of genes were intersected, which identified 348 or 379 genes being commonly up- or downregulated in Mo-M-CSF and Mo-GM-CSF compared to CD14^{+} MO, respectively. The intersections were displayed in the form of Venn Diagrams. Additionally, the Fold-Changes against CD14^{+} MO for the union of the 348 and 379 genes (y-axes) were displayed versus the ranks of the corresponding Fold-Changes (x-axes), once ranked according to the values for Mo-M-CSF and once according to the values for Mo-GM-CSF. Second, DEG were determined directly between Mo-M-CSF and Mo-GM-CSF (Fold-Change > 2 or < -2 and FDR-adjusted p value < 0.05 using one way ANOVA). After reducing the results to a single probe per gene as described before, 124 genes being upregulated in Mo-GM-CSF compared to Mo-M-CSF, and 73 genes being upregulated in Mo-M-CSF compared to Mo-GM-CSF, were identified. Both lists were used as input to perform Gene Ontology Enrichment Analysis (GOEA) in PGS, where 646 as well as 444 significantly enriched GO terms (Enrichment p value < 0.05) were identified for the Mo-GM-CSF as well as Mo-M-CSF specific genes, respectively. Both lists of significant GO terms were used as input for REVIGO, which clusters semantically similar GO terms together and generates a representative GO term name to facilitate the interpretation of long GO term lists. The identified representative GO term names were then visualized as Treemaps, where each rectangle represents one of the representative GO term names. Those rectangles were further joined into "superclusters" by REVIGO, indicated by different colors and a common super-representative GO term name. For simplicity, only the super-names were displayed.

Expression of genes linked to YG bead uptake, motility or OCR/ECAR

To link the experimental data back to the transcriptome data, lists of genes were generated, which had expression patterns being analogous to the observed functional outcomes between Mo-M-CSF, Mo-GM-CSF and Mo-GM-CSF$^{IL-4(0-72h)}$. For YG bead uptake, the pattern was generated by requiring a Fold-Change > 1.3 between Mo-M-CSF and Mo-GM-CSF > 1.3 as well as between Mo-GM-CSF and Mo-GM-CSF$^{IL-4(0-72h)}$. For migration, Fold-Changes > 1.3 were required between Mo-GM-CSF$^{IL-4(0-72h)}$ and Mo-GM-CSF, as well as between Mo-GM-CSF and Mo-M-CSF. For OCR/ECAR, Fold-Changes > 1.3 were required between Mo-GM-CSF$^{IL-4(0-72h)}$ and Mo-GM-CSF, and also between Mo-GM-CSF$^{IL-4(0-72h)}$ and Mo-M-CSF > 1.3, as well as a Fold-Change < 1.3 between Mo-GM-CSF and Mo-M-CSF. Using literature (references see manuscript), those lists were screened for genes, which were previously linked to either YG bead uptake, motility or OCR/ECAR, respectively. The resulting gene sets were displayed in the form of heatmaps, where rows were ordered based on hierarchical clustering using Euclidean distance and average linkage.

Visualization of cytokine responses

Protein expression values of 4 representative donors were averaged and displayed in the form of a heatmap using the function heatmap.2 of the R package gplots. Not detected values were set to 0. Rows were ordered alphabetically.

Handling of the RNA-Seq time kinetics dataset

The raw fastq-files were aligned against the human genome hg19 using TopHat v2.1.0 with default options. The resulting BAM files were imported into PGS, and mRNA quantification was performed against the hg19 RefSeq Transcript database version 2015-08-04 to obtain read counts for each individual RefSeq gene. This resulted in a read count table containing 20,359 genes. The dataset was then normalized using DESeq2 and the table of normalized read counts was imported back into PGS. Then, the technical variation introduced by the different donors was removed as batch effect. Finally, only 12,794 expressed genes were kept, which had a normalized mean read count of at least 10 in at least one of the eight investigated groups.

Analysis and visualization of RNA-Seq time kinetics dataset

Based on the 12,974 present genes, a PCA (PC1 versus PC2) as well as a self-organizing map (SOM) clustering were generated. Additionally, the top 1000 most variable genes across the dataset were visualized in the form of a heatmap, where rows and columns

were ordered based on hierarchical clustering using Euclidean distance and average linkage. Based on the row-wise structure, the genes were grouped together into 5 clusters, of which a few genes were highlighted next to the heatmap.

Co-expression network for the time kinetics dataset

At first, all normalized read counts < 1 were set to 1 to avoid spurious Fold-Changes. Then, DEG (Fold-Change > 1.5 or < −1.5 and FDR-corrected p value < 0.05 using a two-way ANOVA including the donor as a random effect) were determined for each of the 7 conditions compared to the mean over all 7 conditions. The union of the resulting 7 lists comprised 3,114 genes, whose expression profiles were imported into BioLayout. A co-expression network was generated by requiring for each gene a correlation of at least 0.87 to at least one other gene. The network was finally composed of 2,775 genes, since smaller networks containing less than 4 genes were excluded. Using Cytoscape, for each cell type the Fold-Change of the respective cell type compared to the overall mean was mapped onto the network and displayed in blue (Fold-Change $\leq$ 1.5) or red (Fold-Change > = 1.5). Based on the Fold-Change patterns, for each condition a condition-specific cluster was generated by hand. Considering only those genes of the clusters, which had a Fold-Change > 1.5 for the corresponding condition compared to the overall mean, examples of genes being solely present in only one of the groups, or of genes being shared between the clusters of two consecutive time points, were highlighted.

IL-4 signature generation

To generate an IL-4 signature, three different publicly available datasets containing myeloid cells treated with IL-4 were downloaded and processed. From the first, $CD14^+$ MO (Mo1-3) and Mo-GM-$CSF^{IL4(0\text{-}120h)}$ cells (Sample: 5d veh DC1-3) were used (GEO: GSE13762). The raw Affymetrix data of those samples was imported into PGS using RMA background correction, quantile normalization and log_2-transformation. From the second, $CD14^+$ MO (MO at time point 0h of donors # D1 - # D3) and $Mo^{IL4\ (0\text{-}48h)}$ (MO at time point 48h cultured in presence of IL-4 of donor # D1 - #D3) were considered (GEO: GSE35304). The raw Agilent data of those samples was imported into PGS using quantile normalization and log_2-transformation. From the third, Mo-M-$CSF^{(0\text{-}168h)}$ (resting fully differentiated MAC (7 days of culture), rep 1-3) and Mo-M-$CSF^{IL4(168\text{-}192h)}$ (alternative activated MAC (M2) rep 1-3) were used (GEO: GSE32164). The raw Affymetrix data of those samples were imported into PGS using RMA background correction, quantile normalization and log_2-transformation. Within each of the three datasets, the technical variation introduced by the donor was removed as batch effect. Then, differentially expressed probes (Fold-Change > 1.5 or < −1.5 and p value < 0.05 using a two-way ANOVA including the donor as a random effect) were determined within each dataset independently. Finally, after identifying genes being commonly upregulated in the IL-4 conditions of at least two datasets, and similarly being commonly upregulated in the untreated conditions of at least two datasets, 457 genes being induced by IL-4, and 498 genes being repressed by IL-4 were determined, respectively.

Handling of the siRNA αNCoR2 dataset

The raw fastq-files were also aligned against the human genome hg19 using TopHat v2.1.0 with default options. The resulting BAM files were imported into PGS, and mRNA quantification was performed against the hg19 RefSeq Transcript database version 2015-08-04 to obtain read counts for each individual RefSeq gene. This resulted in a read count table containing 19,384 genes. The dataset was then normalized using DESeq2 and the table of normalized read counts was imported back into PGS. Finally, only 12,816 expressed genes were kept, which had a normalized mean read count of at least 10 in at least one of the two investigated groups. All normalized read counts < 1 were set to 1 to avoid spurious Fold-Changes.

Analysis and visualization of the siRNA αNCoR2 dataset

Based on the 12,816 present genes, a PCA (PC1 versus PC2) was generated. Additionally, the expression profiles of the 1,834 genes being variable (p value < 0.05) across the dataset were visualized in the form of heatmap, where both rows and columns were ordered based on hierarchical clustering using Euclidean distance and average linkage. Based on the resulting row-wise structure, the genes were grouped into clusters, of which a few genes were highlighted next to the heatmap. The variable genes were also displayed within a scatterplot, displaying the log_2-mean expression of the scrambled RNA samples (x axis) versus the log_2-mean expression of the siRNA αNCoR2 samples. In red and blue were specifically highlighted, which of the genes were also part of the previously established signatures of IL-4 induced and repressed genes, respectively.

GSEA

To test for human IL-4 signature enrichment in samples treated with either anti-NCOR2 or scrambled shRNA we performed GSEA. GSEA is a computational tool that determines whether a set of genes show statistically significant, concordant differences between two conditions (http://www.broadinstitute.org/gsea/index.jsp). We have used the normalized data table as input and either genes up- or downregulated in the IL-4 signature as gene sets for the GSEA. The 'Signal2Noise' ranking metric was used and the gene sets were chosen showing significant change at FDRc < c0.25, nominal P valuec < c0.05 and 1,000 gene set permutations.

QUANTIFICATION AND STATISTICAL ANALYSIS

Statistical analysis was performed using SigmaPlot (Systat Software Inc.). Statistical tests used are described in the according figure legend. Mean (±SEM) was indicated as horizontal lines. If not otherwise specified n represents number of biological replicates.

DATA AND SOFTWARE AVAILABILTIY

All expression data related to this manuscript can be found at Gene Expression Omnibus under the accession number GSE96719.

Immunity, Volume *47*

Supplemental Information

Cellular Differentiation of Human Monocytes Is Regulated by Time-Dependent Interleukin-4 Signaling and the Transcriptional Regulator NCOR2

Jil Sander, Susanne V. Schmidt, Branko Cirovic, Naomi McGovern, Olympia Papantonopoulou, Anna-Lena Hardt, Anna C. Aschenbrenner, Christoph Kreer, Thomas Quast, Alexander M. Xu, Lisa M. Schmidleithner, Heidi Theis, Lan Do Thi Huong, Hermi Rizal Bin Sumatoh, Mario A.R. Lauterbach, Jonas Schulte-Schrepping, Patrick Günther, Jia Xue, Kevin Baßler, Thomas Ulas, Kathrin Klee, Natalie Katzmarski, Stefanie Herresthal, Wolfgang Krebs, Bianca Martin, Eicke Latz, Kristian Händler, Michael Kraut, Waldemar Kolanus, Marc Beyer, Christine S. Falk, Bettina Wiegmann, Sven Burgdorf, Nicholas A. Melosh, Evan W. Newell, Florent Ginhoux, Andreas Schlitzer, and Joachim L. Schultze

Supplementary figure 1

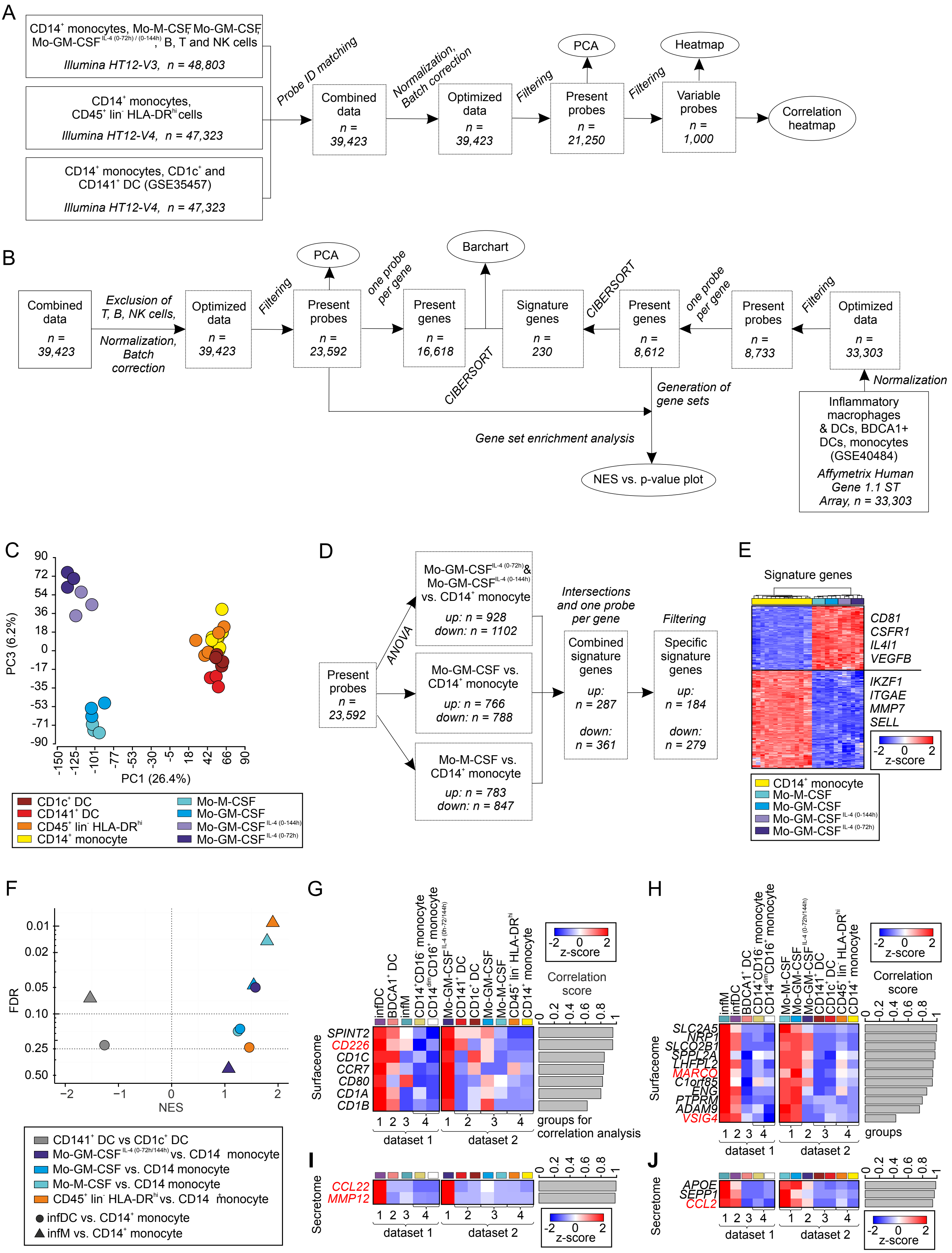

Figure S1 related to Figure 1

Relationship of *in vitro* activated monocyte-derived cells. (A-B) Analysis workflows for Figure 1 and S1 for datasets (**A**) with T, B and NK cells or (**B**) without. (**C**) PCA based on 23,952 present probes. Displayed are PCs 1 versus 3. (**D**) Workflow to identify specifically expressed genes in monocyte-derived cells compared to monocytes, or vice versa. (**E**) Heatmap of specifically expressed genes in monocyte-derived cells compared to monocytes, or vice versa. Z-transformed log_2-expression values scaled to a minimum of -2 (blue) and a maximum of 2 (red). Important genes of each group are depicted next to the heatmap. (**F**) Scatterplot showing the normalized enrichment score (NES, x-axis) versus the false discovery rate (FDR, y-axis) obtained after GSEA. Gene sets were comprised of specifically expressed genes in infM or infDCs compared to $CD14^+$ monocytes, respectively. Enrichments were estimated for pairwise comparisons of monocyte-derived cells versus $CD14^+$ Monocytes, and for the comparison of $CD141^+$ versus $CD1c^+$ DCs. (**G-J**) Heatmaps of genes encoding (**G, H**) surface markers or (**I, J**) secreted molecules specifically expressed in (**G, I**) infDCs compared to infM, $BDCA1^+$ DCs and monocytes (left, dataset 1), as well as in Mo-$GMCSF^{IL4 (0-72h)}$ cells compared to all other investigated monocyte-derived cells, $CD14^+$ monocytes and DCs (right, dataset 2), or in (**H, J**) infM compared to infDCs, $BDCA1^+$ DCs and monocytes (left, dataset 1), as well as in Mo-GMCSF and Mo-MCSF cells compared to Mo-$GMCSF^{IL4(0-72h)}$ cells, $CD14^+$ monocytes and DCs (right, dataset 2). Pearson correlation values were calculated between the indicated group patterns of dataset 1 versus dataset 2 and displayed as a barplot next to the heatmaps. Genes encoding (**G, H**) surface markers or (**I, J**) secreted molecules are displayed, with a correlation of at least 0.4 between the corresponding two datasets. Genes further analyzed in Figure 1 **I-K** are highlighted in red. Z-transformed log_2-expression values were and scaled to a minimum of -2 (blue) and a maximum of 2 (red).

Supplementary figure 2

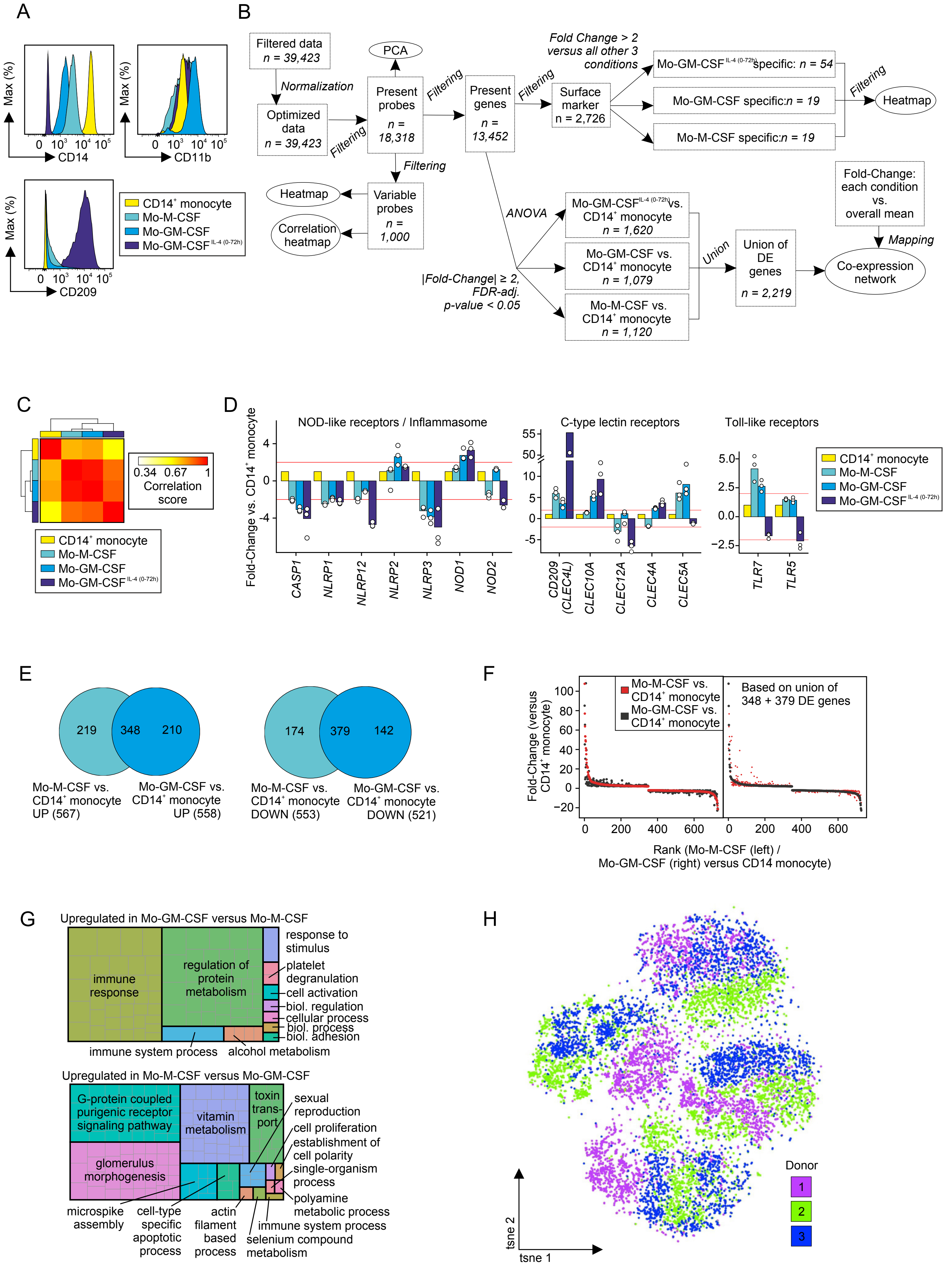

Figure S2 related to Figure 2

Mo-GMCSF$^{IL4(0-72h)}$ are most distinct from Mo-MCSF and Mo-GMCSF. (A) Histograms show relative expression of CD14, CD11b and CD209 on CD14^{+} monocytes, Mo-MCSF, Mo-GMCSF and Mo-GMCSF$^{IL4(0-72h)}$ analysed by flow cytometry. Representative data from 4 different donors is shown. (**B**) Workflow for the major bioinformatic analyses in Figure 2 and S2. (**C**) Heatmap visualizing pearson correlation values (pearson correlation coefficient matrix, PCCM) calculated pairwise between all cell types on the basis of the top 1000 most variable probes. The color scaling ranges from white (low correlation) via yellow to red (high correlation). (**D**) Barplots showing the Fold-Changes for all three monocyte-derived cell types compared to CD14^{+} monocytes for genes encoding NOD-like receptors or being linked to the inflammasome (top), as well as for genes encoding C-type lectin (bottom left) or Toll-like (bottom right) receptors. Additionally, the Fold-Changes for all replicates of a single cell type compared to the mean expression of the CD14^{+} monocyte replicates are displayed as dots. (**E**) Venn Diagrams of genes being significantly up- (Fold-Change > 2 and FDR-adjusted p-value < 0.05, top) or down-regulated (Fold-Change < -2 and FDR-adjusted p-value < 0.05, bottom) in whether Mo-MCSF (left) or Mo-GMCSF (right) compared to CD14^{+} monocytes. (**F**) Rank plots of genes being significantly up- or downregulated for both Mo-GMCSF and Mo-MCSF compared to CD14^{+} monocytes (union of both overlapping regions demonstrated in **E**). Displayed are the ranks (x-axis) of the Fold-Changes compared to CD14^{+} monocytes (y-axis), whether sorted according to Mo-MCSF (top) or Mo-GMCSF (bottom). (**G**) Tree plot summarizing significantly enriched (Enrichment p-value < 0.05) biological processes identified by Gene Ontology Enrichment Analysis (GOEA) for genes being significantly up- or downregulated (Fold-Change > 2 or < -2 and FDR-adjusted p-value < 0.05) directly between Mo-GMCSF and Mo-MCSF. (**H**) *t*-SNE composite dimensions (tsne1 and 2) of CD14^{+} monocytes, Mo-MCSF, Mo-GMCSF and Mo-GMCSF$^{IL4(0-144h)}$ analysed by mass cytometry and colored according to donor identity (n=3).

Supplementary figure 3

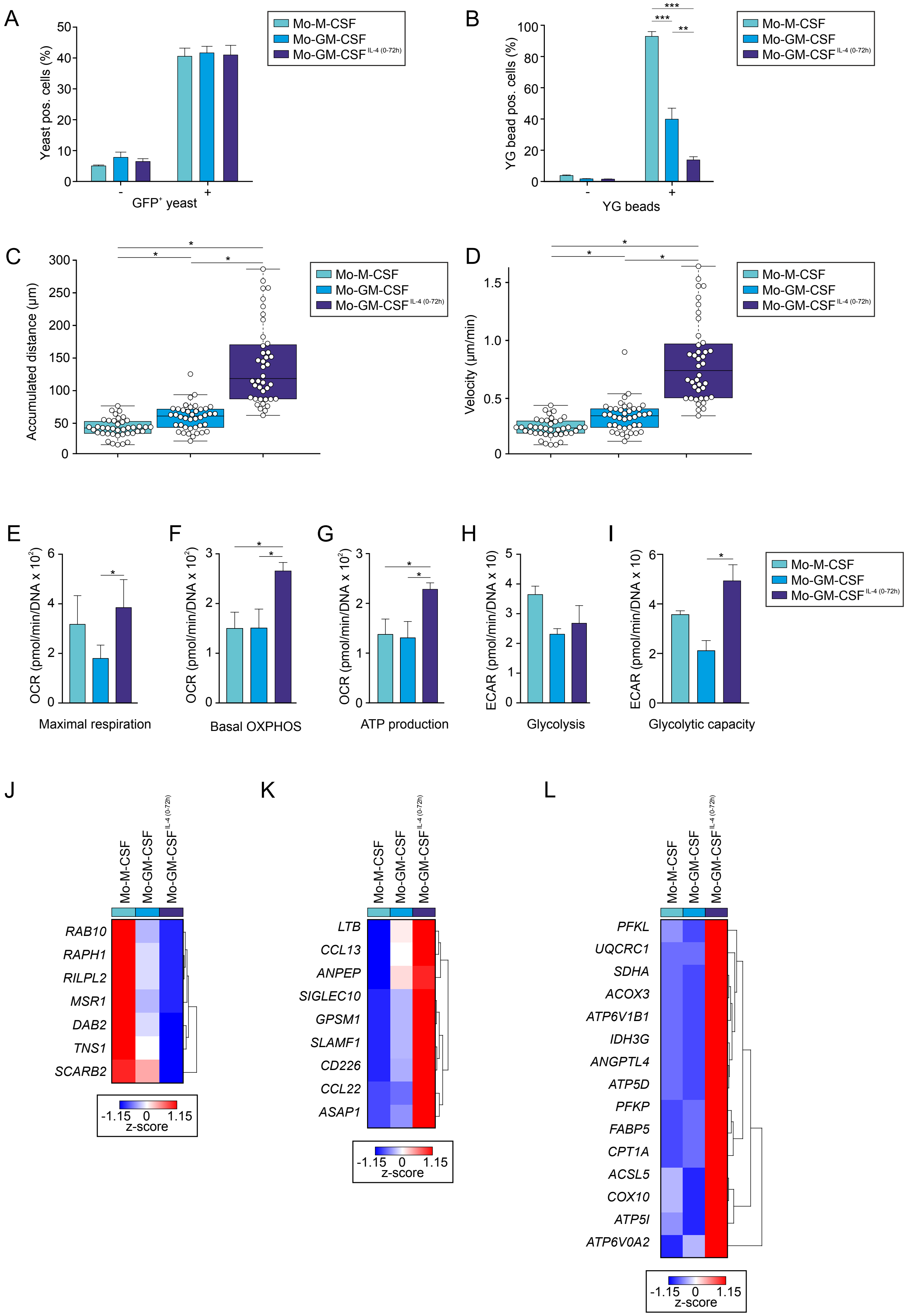

Figure S3 related to Figure 3

Prediction of activated monocyte functionality. (A) Quantification of the flow cytometric analysis of Mo-MCSF, Mo-GMCSF and Mo-GMCSF$^{IL4(0-72h)}$ after 1h of incubation with GFP^{+} yeast or without (n=3, mean + SEM). Statistical significance between cell types after 1h of incubation was determined using one-way RM ANOVA, with $p > 0.05$. **(B)** Quantification of the flow cytometric analysis of Mo-MCSF, Mo-GMCSF and Mo-GMCSF$^{IL4(0-72h)}$ after 4hrs of incubation with YG beads (n=5-6). Statistical significance was determined using one-way RM ANOVA and Tukey's method for multiple test correction, with $^{**}p < 0.01$ and $^{***}p < 0.001$. (**C**) Quantification of accumulated distance and (**D**) average migratory speed (velocity) of motile Mo-MCSF, Mo-GMCSF and Mo-GMCSF$^{IL4(0-72h)}$ corresponding to **Figure 3C**. Results show one representative experiment out of three. Statistical significance was determined using Kruskal-Wallis one-way ANOVA on ranks and Tukey's method for multiple test correction, with $^{*}p < 0.05$. (**E**) Maximal respiration, (**F**) basal OXPHOS, (**G**) ATP synthesis, (**H**) glycolysis and (**I**) glycolytic capacity Mo-MCSF, Mo-GMCSF and Mo-GMCSF$^{IL4(0-72h)}$, respectively. Results are pooled data shown as mean + SEM from n = 3 replicates. Statistical significance was determined using one way ANOVA with $^{*}p < 0.05$. (**J-L**) Heatmaps of genes being directly or indirectly linked (see supplementary information) to (**J**) YG bead uptake, (**K**) motility or (**L**) OCR/ECAR, which have expression patterns on the transcriptional level being analogous to the observed functional outcomes summarized in G. The $\log_2$-expression values were z-transformed and scaled to a minimum of –1.15 (blue) and a maximum of 1.15 (red), respectively.

Supplementary figure 4

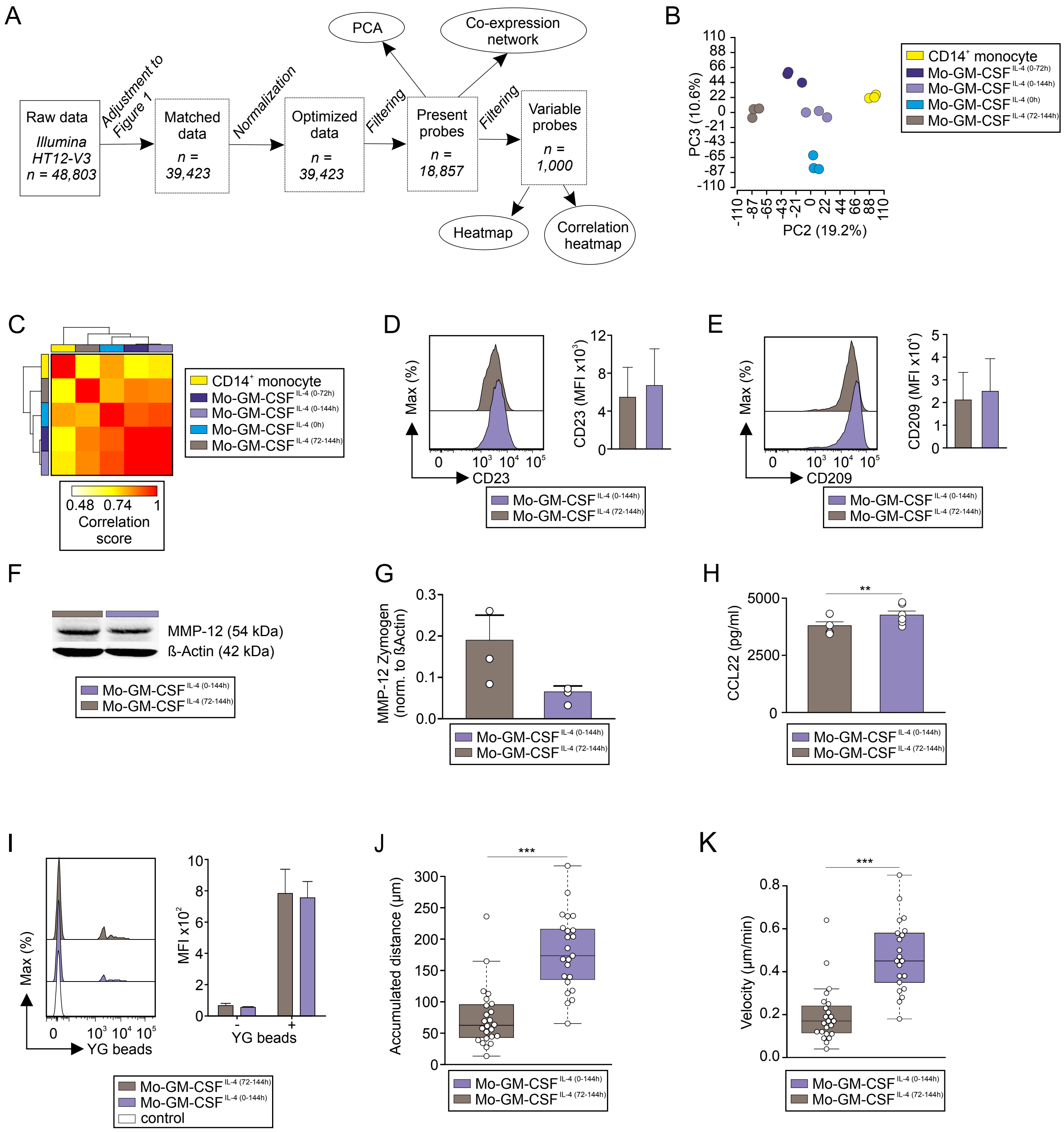

Figure S4 related to Figure 4

Mo-GMCSF$^{IL4(0\text{-}144h)}$ differ from Mo-GMCSF$^{IL4(72\text{-}144h)}$ monocyte-derived cells. (A) Workflow for Figure 4 and S4. (**B**) Principal component analysis (PCAs) based on 18,857 present probes. Principal components (PCs) 2 versus 3 are shown, which account for 19.2% versus 10.6% of the total variance, respectively. (**C**) Heatmap visualizing pearson correlation values (pearson correlation coefficient matrix, PCCM) calculated pairwise between all cell types on the basis of the top 1000 most variable genes. The color scaling ranges from white (low correlation) via yellow to red (high correlation). **(D-E)** Histograms show relative expression of (**D**) CD23 and (**E**) CD209 on Mo-GMCSF$^{(72h\text{-}144h)}$ and Mo-GMCSF$^{IL4(0\text{-}144h)}$ analysed by flow cytometry. Bar plots show quantification of the mean fluorescence intensity (MFI) of the (**D**) CD23 and (**E**) CD209 signal on Mo-GMCSF$^{(72h\text{-}144h)}$ or Mo-GMCSF$^{IL4(0\text{-}144h)}$. Representative data shown (n=3, mean + SEM). Statistical significance was determined using paired t-test, with p = 0.415 and p = 0.399 for (**D**) and (**E**), respectively. **(F)** Representative western blot image of MMP12 and β-Actin protein expression in Mo-GMCSF$^{(72h\text{-}144h)}$ or Mo-GMCSF$^{IL4(0\text{-}144h)}$ (n=3). **(G)** Statistical quantification of MMP12 protein expression normalized to β-Actin in Mo-GMCSF$^{(72h\text{-}144h)}$ or Mo-GMCSF$^{IL4(0\text{-}144h)}$ using western blot (n=3, mean + SEM). Statistical significance was determined using paired t-test with p = 0.229**. (H)** Analysis of culture supernatants of Mo-GMCSF$^{(72h\text{-}144h)}$ or Mo-GMCSF$^{IL4(0\text{-}144h)}$ for CCL22 using ELISA (n=6, 3 independent donors, 2 technical replicates)). Data are shown as mean + SEM. Statistical significance was determined using paired t-test with $**p < 0.01$**. (I)** Flow cytometric analysis of Mo-GMCSF$^{(72h\text{-}144h)}$ or Mo-GMCSF$^{IL4(0\text{-}144h)}$ after 4hrs of incubation with YG beads (n=6, mean + SEM). Statistical significance between cell types after 4h of incubation was determined using paired t-test with p = 0.83. (**J**) Quantification of accumulated distance and (**K**) average migratory speed (velocity) of motile myeloid cells corresponding to Figure 4F. Results show one representative experiment out of three. Statistical significance was determined using Student's t-test in (**J**) and Mann-Whitney Rank Sum test in (**K**) with $***p < 0.001$.

Supplementary figure 5

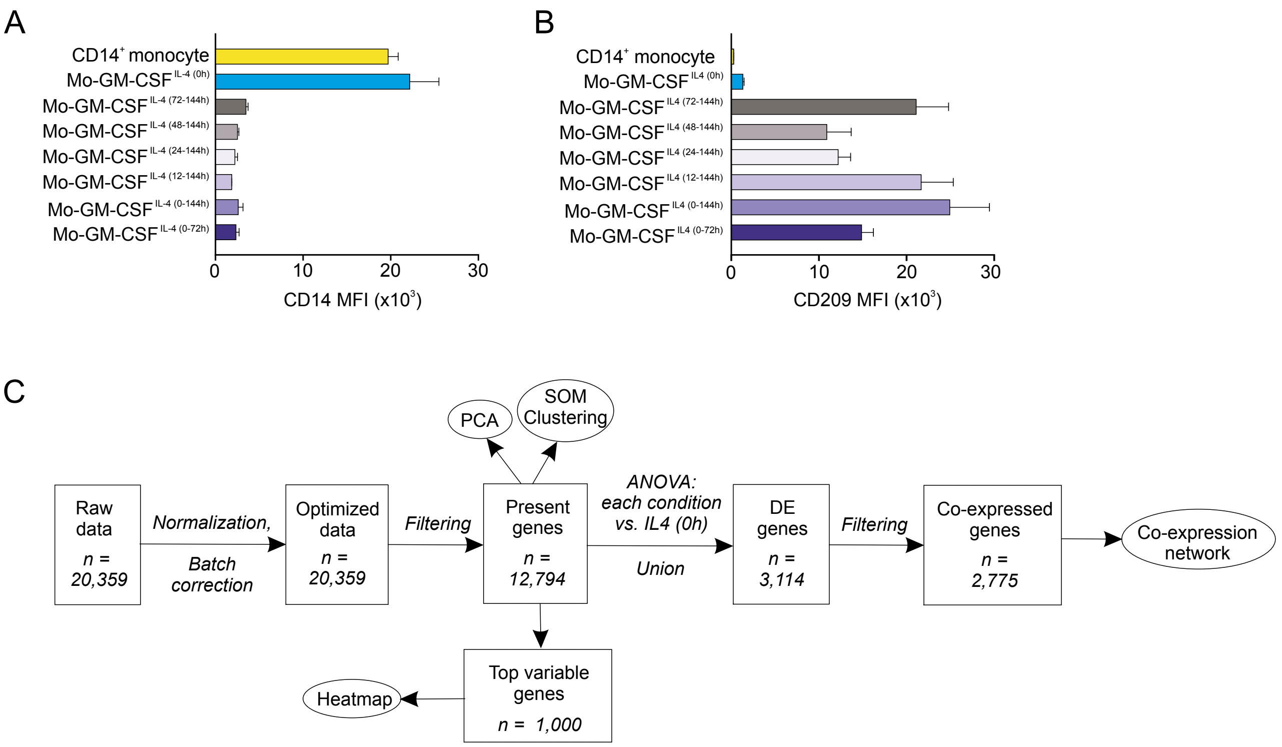

Figure S5 related to Figure 5

Timing of IL4 determines transcriptional regulation in activated monocytes. (A-B) Flow cytometric analysis of the MFI of (**A**) CD14 and (**B**) CD209 on the depicted cell types (n=4). (**C**) Workflow for the RNA-Seq data analysis in Figure 5.

Supplementary figure 6

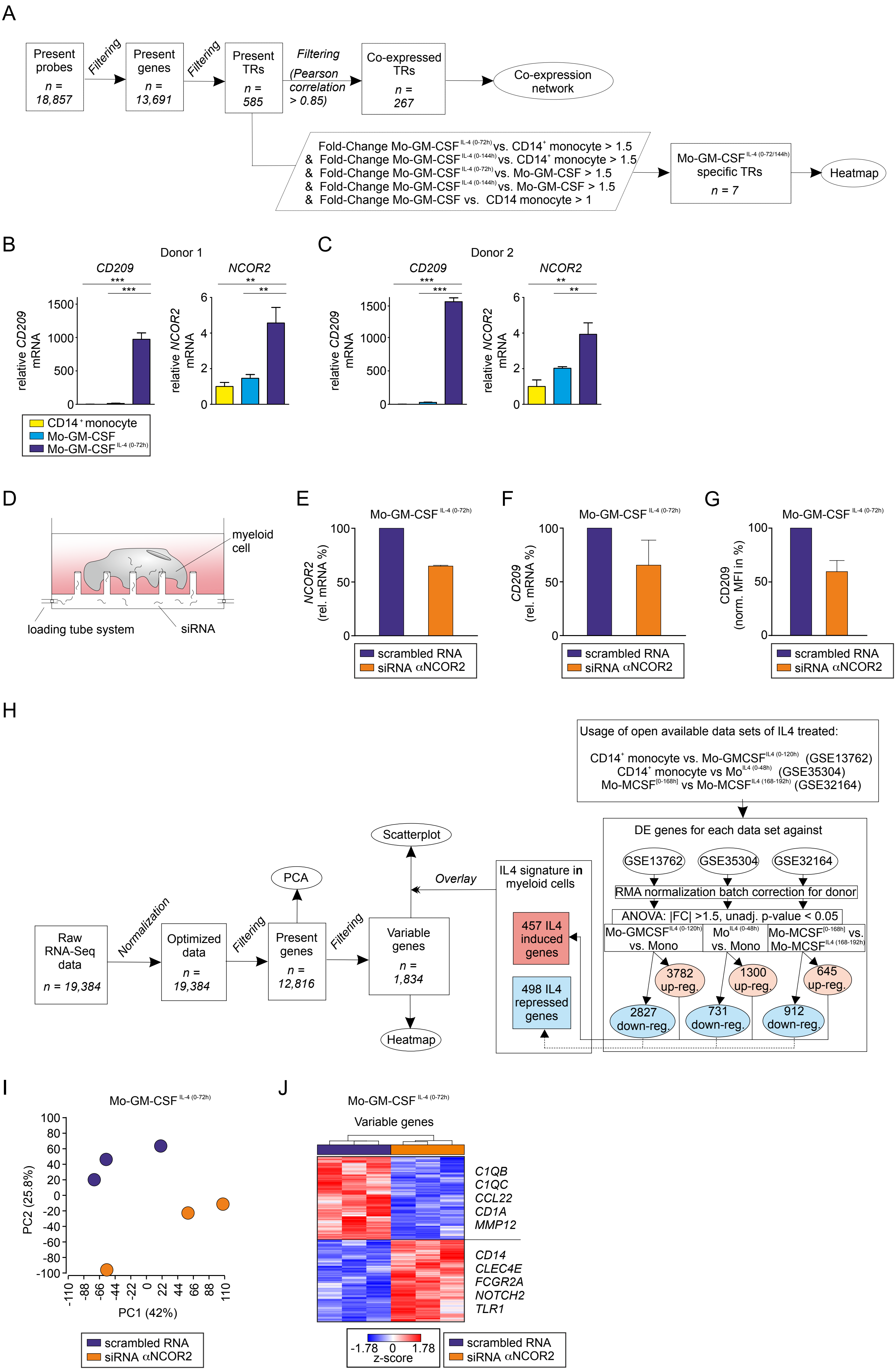

Figure S6 related to Figure 6

NCOR2 is a transcriptional regulator of Mo-GMCSF$^{IL4(0-72/144h)}$. (A) Workflow for Figure 6 and S6. **(B)** *Realtime* PCR measurement of *NCOR2* and *CD209* mRNA levels relative to *GAPDH* mRNA levels in Mo-GMCSF and Mo-GMCSF$^{IL4(0-72h)}$ in donor 1 (mean + standard deviation). **(C)** *Realtime* PCR measurement of *NCOR2* and *CD209* mRNA levels relative to *GAPDH* mRNA levels in Mo-GMCSF and Mo-GMCSF$^{IL4(0-72h)}$ in donor 2 (mean + standard deviation). (**D**) Schema for the experimental setup of the siRNA-based gene knockdown. **(E)** Realtime PCR measurement of *NCOR2* mRNA levels relative to *GAPDH* mRNA levels in Mo-GMCSF$^{IL4(0-72h)}$ (n=2, mean + standard deviation). **(F)** Realtime PCR measurement of *CD209* mRNA levels relative to *β-Actin* mRNA levels in Mo-GMCSF$^{IL4(0-72h)}$ (n = 2, mean + standard deviation). **(G)** Quantification of the normalized MFI of CD209 on Mo-GMCSF$^{IL4(0-72h)}$ (n = 2, mean + standard deviation). (**H**) Workflow for the analysis of the siRNA gene knockdown dataset. (**I**) Principal component analysis (PCA) based on 12,816 present genes. Displayed are principal components (PCs) 1 versus 2, which account for 42% versus 25.8% of the total variance, respectively. (**J**) Heatmap of the top 1000 genes being most variable across the dataset. Rows were ordered based on hierarchical clustering. The expression values were z-transformed and scaled to a minimum of -1.78 (blue) and a maximum of 1.78 (red). A few genes of each group are depicted on the right side next to the heatmap.
